# Progress in the Application of Food-Grade Emulsions

**DOI:** 10.3390/foods11182883

**Published:** 2022-09-17

**Authors:** Yilin Jie, Fusheng Chen

**Affiliations:** College of Food Science and Engineering, Henan University of Technology, Zhengzhou 450001, China

**Keywords:** emulsion dispersion system, dispersed phase, interface structure, macroscopic scale, soft matter

## Abstract

The detailed investigation of food-grade emulsions, which possess considerable structural and functional advantages, remains ongoing to enhance our understanding of these dispersion systems and to expand their application scope. This work reviews the applications of food-grade emulsions on the dispersed phase, interface structure, and macroscopic scales; further, it discusses the corresponding factors of influence, the selection and design of food dispersion systems, and the expansion of their application scope. Specifically, applications on the dispersed-phase scale mainly include delivery by soft matter carriers and auxiliary extraction/separation, while applications on the scale of the interface structure involve biphasic systems for enzymatic catalysis and systems that can influence substance digestion/absorption, washing, and disinfection. Future research on these scales should therefore focus on surface-active substances, real interface structure compositions, and the design of interface layers with antioxidant properties. By contrast, applications on the macroscopic scale mainly include the design of soft materials for structured food, in addition to various material applications and other emerging uses. In this case, future research should focus on the interactions between emulsion systems and food ingredients, the effects of food process engineering, safety, nutrition, and metabolism. Considering the ongoing research in this field, we believe that this review will be useful for researchers aiming to explore the applications of food-grade emulsions.

## 1. Introduction

Dispersion systems integrating different phases (e.g., solids, liquids, liquid crystals, and gases) and hydrophilic/hydrophobic substances are widely found in nature and industry. Examples of such systems include paints, inks, pesticides, bitumen, toothpaste, soap, detergents, foam, glue, smoke, dust, soil, pearls, cells, egg yolk membranes, oil bodies, milk, ice cream, cakes, butter, beer, mayonnaise, jam, juice, and soup. Among these systems, interface-dominated systems contain small structural units and large interface areas, and they can be classified into traditional emulsions, nanoemulsions, Pickering emulsions, multiple emulsions, high internal phase emulsions (HIPEs), emulsion gels, suspensions, and foams, among others.

Briefly, an emulsion is simply a mixture of two (or more) liquids that are otherwise immiscible. The mixing is made possible when one liquid is forced into the droplet form inside the other liquid, with the droplets being stabilized by emulsifying agent. The most common are the traditional emulsions, generally consist of small spherical droplets of two liquids stabilized through surfactants. Pickering emulsions are designed by replacing traditional surfactants with solid particles as emulsifiers. Multilayer emulsions emphasize that consist of multiple layers of the emulsifier that cooperatively stabilize the emulsion. Nanoemulsions, as the name implies, consist of dispersed-phase droplets on the nanometer scale. HIPEs feature a high-volume fraction of the internal phase (*ϕ* ≥ 0.74), exhibiting advantageous structural and functional properties, such as excellent stability and oxidation resistance. Double emulsions generally contain three (or more) immiscible liquids (W/O/W, O/W/O, etc.) [1]. Blended emulsions usually refer to the mixing of two or more emulsions from different sources (such as soy–cow-blended milk) [2]. Apart from the aforementioned emulsions, there are two other types of emulsions, as follows. First, the natural oil–body emulsion, an example of which is found in the seeds of oil crops such as soybeans, which store cellular triglycerides in the form of droplets. Second, the self-emulsifying formulations are composed of oil, surfactant, and cosolvent.

The eco-friendliness, safety, favorable structural characteristics, and functional abundance/efficiency of food-grade emulsions enable their diverse applications and provide a foundational material structure for further development. Consequently, many researchers have studied the structures and functions of these emulsions, in addition to their related emulsification and stabilization mechanisms, to develop novel emulsifiers and applications. Moreover, further development of emulsion preparation technology, real-time process visualization of accurate structural characterization, and precision analysis tools will enable better control of the design, development, and evaluation of the functional applications of food-grade emulsions [3]. Despite their diversity, all food-grade emulsions are based on continuous and dispersed phases and the interfaces between them. Importantly, the structures and functions of these three constituents are different but interrelated, and so they play different roles in the formation of a functional and interconnected overall structure. This structure can be regulated and influenced by many environmental factors, and it can be tuned to fit desirable emerging applications.

As the functional applications of food emulsion systems are closely related to the structures and functions of their components and of the whole assembly, it is necessary to reach a deep understanding of the influencing factors at all levels to facilitate the development of superior emulsion systems and expand their application scope. The systematic study of variable-scale emulsions is a highly interdisciplinary field requiring knowledge of basic science; specific disciplines (e.g., soft matter physics, supramolecular chemistry, and advanced characterization methods); and the engineering technologies used in food production processes. Moreover, safety and nutritional values should be considered during the preparation of practical materials, especially in the context of food-grade applications.

**Figure 1:**
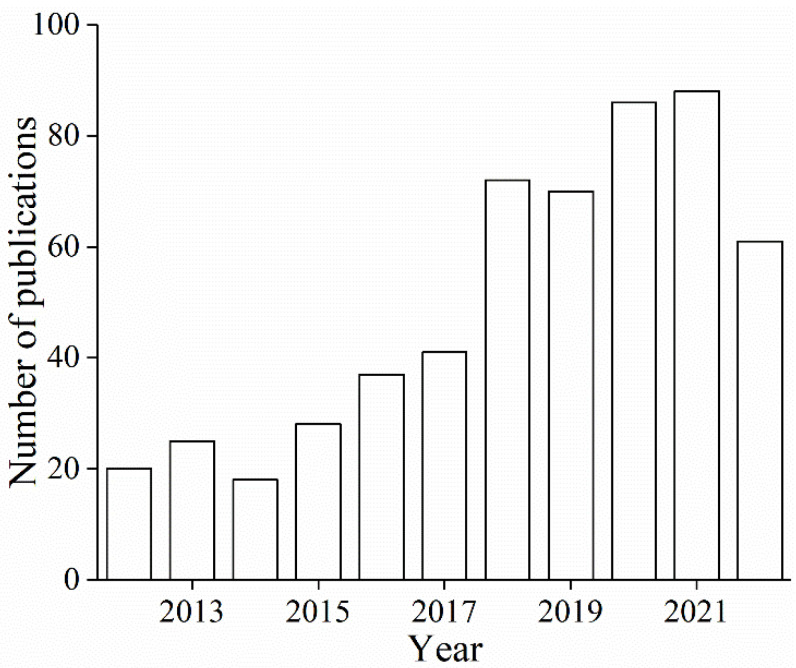
Number of publications related to the “application of food-grade emulsions” from a search of the Web of Science database (with the most recent data downloaded on 5 September 2022).

On the basis of the theme of “application of food-grade emulsions”, 635 search results were retrieved on the Web of Science, of which 546 were published in the last 10 years. Figure 1 present the number of publications related to the theme, and substantial interests have been devoted to the theme since 2012. In retrospect, the application of food-grade emulsions, from versatile active ingredient delivery vehicles to the design of structured food materials, has attracted wide interests in the field of food science technology, chemistry, nutrition dietetics, science technology of other topics, agriculture, materials science, engineering, and pharmacology pharmacy. Recently, these efforts mainly focused on texture design and modification of edible products; lipid oxidation reduction (protecting easily oxidized functional ingredients); systems that influence substance digestion and absorption in the body; and various material applications (such as 3D/4D printed food, porous materials, foams, and other functional materials templates). In conclusion, food-grade emulsions show good application potential and development value in the areas of food, cosmetics, chemical engineering, and bioengineering.

**Figure 2:**
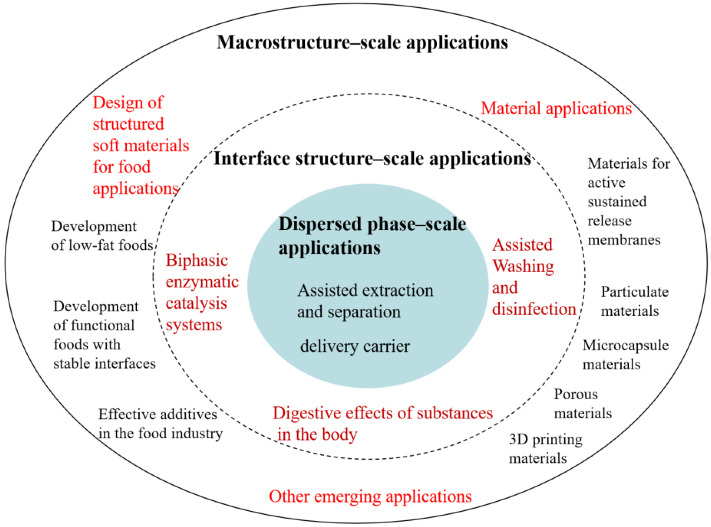
Applications of food-grade emulsions organized in terms of the dispersed phase, interface structure, and macrostructure scales.

To date, no comparative summary of applied research based on the essential characteristics of emulsion systems at variable-scale hierarchies has been published. Therefore, to account for the existing issues and further promote efficient utilization of food-grade emulsions, i.e., those containing bio-based interfacial active substances as emulsifiers, this review classifies and summarizes their applications on three scales (dispersed phase, interface structure, and macroscopic scales) (Figure 2), in addition to providing future research directions and highlighting the corresponding control methods and factors of influence. Potential research trends are presented on different scales from the viewpoints of basic science theory and technology, production process engineering, materials, and nutrition safety, among others.

## 2. Applications on the Dispersed-Phase Scale

### 2.1. Delivery Carriers for Active Substances

Dispersed-phase droplets have been reported to act as capsules that protect bioactive soft substances in the inner phase. Specifically, they can act as carriers for the packaging; protection; and delivery of active ingredients (e.g., curcumin, capsaicin, probiotics, and phytochemicals) to improve product stability, solubility, flavor, and taste. Therefore, the development of emulsion delivery systems, such as natural oil–body emulsions, conventional emulsions, nanoemulsions, Pickering emulsions, double emulsion [1], blended emulsions [2], HIPEs, and self-emulsifying formulations, promotes the innovative application of functionally active substances and is currently an emerging area in the research on foods, pharmaceuticals, materials, and fine chemicals. For example, soft materials exhibiting a range of properties have been designed and delivered using various emulsion carrier technologies to take advantage of the different dispersed-phase properties. As previously reported [4], the dispersed phase of an emulsion system can encapsulate the same soft materials despite differences in the carrier structure, and this is achieved throughout diversified selection and a design based on the nature of the internal packaging. 

For example, Liu et al. [5] demonstrated that a natural soybean oil–body emulsion can be used as a carrier for the delivery of curcumin. Self-emulsifying formulation, composed of an oil, a surfactant, and a cosolvent, was easily produced on a large scale and exhibited a high carrying capacity, which resulted in an enhanced bioavailability and efficacy for the active substances. As an example, Wang et al. [6] prepared a solid self-emulsifying system for the delivery of dihydromyricetin, which allowed them to overcome the poor water solubility and short biological half-life of this substance, thereby improving its antioxidant performance and bioavailability and providing a feasible solution for its application in foods and beverages. In addition, Pickering emulsions have been used to encapsulate flavor compounds to hinder their volatilization and oxidative degradation whilst also promoting their effective dispersion into commodities to achieve continuous aroma release [7]. Moreover, they have been used in cannabinoid delivery to prevent poisoning [8]. Furthermore, gelation of the intermediate oil phase of a Pickering double emulsion has been demonstrated to significantly improve the emulsion stability while also leading to an adjustable flavor release [9]. Moreover, Chen and Tang [10] used spirulina phycocyanin-stabilized transparent HIPEs containing a strong antioxidant to encapsulate fat-soluble bioactive substances and achieve their slow release. Additionally, temperature-responsive HIPEs can be used to transport flammable, explosive, volatile, and toxic liquids [11]. 

In the case of assembling biomacromolecular nutraceuticals for nanoencapsulation and delivery applications, the good interfacial properties of these substances allow them to act as emulsifiers. As a result, the loaded active substances are located at the interface rather than being encapsulated in the dispersed phase [12]. In view of their high resistance to aggregation, nanoparticle-stabilized Pickering emulsions can effectively protect and enhance the absorption of substances in simulated gastrointestinal environments. For example, nanoparticles of soy protein–anthocyanin complexes can be used to prepare stable Pickering emulsions for anthocyanin transport [13], while the self-assembled colloidal particles based on pea proteins and grape seed proanthocyanidins can effectively stabilize emulsions and transport proanthocyanidins [14].

Owing to the fact that emulsion delivery systems exhibit broadly variable structural characteristics, (dis)advantages, digestion mechanisms, and kinetics, it is necessary to select an appropriate delivery method according to the chemical properties, composition, and biological activity of the delivery substance. Moreover, the factors affecting the performances of such delivery methods should be determined, and active substance loss under heating or long-term storage must be evaluated. In addition, it is necessary to review and compare the effectiveness of different delivery methods and evaluate their industrialization potential to obtain stable and high-performance delivery systems. To date, a number of studies have used different emulsion delivery systems for the same active ingredient to enhance the activity and availability, systematically analyzing the load capacity and effectiveness, as well as discussing the challenges and safety issues related to different encapsulation technologies [15]. Since emulsion-based products have specific ingredients and processing/storage requirements, the corresponding studies must be performed on a case-by-case basis. To explore innovative release control methods, regulation of the bioavailability and release spectrum of bioactive substances should be evaluated for different release systems. Furthermore, additional in vivo metabolic kinetic studies are required to verify the efficacy and safety of specific nanofunctional delivery carriers. Moreover, the synergy between different strategies, e.g., co-color and encapsulation strategies for anthocyanin stabilization [16], should be explored. Table 1 lists the different emulsion delivery systems reported to date, focusing on their carrier types, specific dispersal systems, delivery substances, and advantages.

### 2.2. Research Prospects for Delivery Carrier Applications

The main factors affecting delivery carrier applications on the dispersed phase scale are the emulsion structure, droplet size, droplet interactions and compositions, dispersion conditions, volume fraction, and the structural properties of the dispersed phase. In addition, it has been reported that the type of dispersed phase and the selection and diversification of the appropriate load materials directly affect the functionality and potential applications of delivery systems [72]. Related research directions therefore include control over the structural morphology and size of the dispersed phase and the selection and diversification of both the dispersed phase and the loading material.

As reported by Wan et al. [73], the bioavailability of a delivered active substance is affected by its properties in addition to the lipid type, composition, structure, content, and physical state. More specifically, lipids containing essential fatty acids or functional lipids can improve food functionality, with medium-chain triglycerides outperforming short- or long-chain ones in enhancing the active substance effectiveness [74]. In addition, Guo et al. [75] found that the oxidation stabilities and biological activities of phytochemical-containing nanoemulsions were highly dependent on the degree of oil saturation, and they demonstrated that the lipid-lowering effects of tocopherol and sesamol increased upon reducing the extent of oil saturation.

The zeta potential of dispersed-phase droplets can affect the stability of the emulsion interface, especially in different application environments (whether or not effectively protect and enhance the absorption of substances in simulated gastrointestinal environments), thus indirectly affecting the load capacity and availability of delivery active ingredient. In particular, when the antibacterial emulsion loaded with antibacterial active substances plays a role, the zeta potential of dispersed phase droplets will directly affect the contact with Gram-positive bacteria and Gram-negative bacteria, thereby affecting the antibacterial performance of the loaded antibacterial substances [25,33].

Regarding the structures and morphologies of dispersed-phase droplets, emulsion applications can be grouped according to their structural properties. Due to the small sizes of the particles present in the dispersed phase, nanoemulsion delivery systems exhibit significantly higher stabilities and soft matter loading capacities than those based on conventional emulsions, and as a result, they can effectively improve the rapid digestion, metabolism, absorption, and bioavailability of the active components. For example, the bioaccessibility of oil-soluble vitamins in plant-based emulsions was found to decrease with an increase in the oil droplet size [76], and the stability and digestibility of fish oil has been shown to increase through the formulation of soybean protein isolate–phosphatidylcholine-stabilized fish oil nanoemulsions [29]. In addition, Pickering emulsions are known to greatly improve the stability and protective effect of the loaded material because of their high resistance to delamination and coalescence [77]. In view of the large volumes of their inner phases, which exceed the minimum volume of closely arranged rigid balls, the dispersed phases of HIPEs exhibit a closely arranged deformation morphology, which greatly improves the oxidation resistance and increases the material loading capacity. Similarly, in contrast to traditional emulsions, composite emulsion–gel delivery systems, which combine the properties of emulsions and gels, have a specific gel-like network structure and stable mechanical properties, thereby leading to a greatly improved stability and the ability to allow sustained release [78,79]. Thus, the development of novel ultra-stable nano-Pickering emulsions, gel-in-water nanoemulsions [80], oleogel-based nanoemulsions [81], or nano-HIPEs for applications in delivery systems is also important. As an example, it has been reported that nanoemulsion gels stabilized by cyclodextrin-based metal–organic frameworks and glycyrrhizic acid exhibit good long-term storage stabilities under highly alkaline and high-temperature conditions, indicating their potential use as delivery systems [82].

Future research should focus on methods for the preparation of interfacial active materials to improve the performances of emulsions with controllable particle sizes and wide-ranging functions. The preparation of multiple emulsions is also an important research direction, which should contribute to the design of multiple efficient delivery systems and is of great significance in terms of expanding the applied research based on the dispersed phase. In addition, environmentally responsive emulsion-based delivery systems [83] for the sustained and controlled (targeted or triggered) release of substances are of interest to improve the bioactivities and bioavailabilities of compounds such as drug molecules. As an example, in the encapsulation of curcumin by Pickering emulsion hydrogels stabilized by carboxymethyl chitosan–sodium alginate, the nanoparticles can control the release of the active substance (curcumin), thereby accelerating wound healing [84]. Moreover, the formation of pectin anti-gastrointestinal emulsions is a promising method for delaying the digestion and colon-targeted release of lipid-based materials [85]. The fabrication of nonliquid emulsion-based delivery systems (i.e., by freeze- and spray-drying) is another direction for future research, which should permit greater association between laboratory and market applications [86]. 

For emulsion systems that deliver bioactive substances, it is necessary to optimize the emulsion processing conditions [87], investigate the actual benefits after entrapment, evaluate the distribution and stability upon storage, and optimize the degradation behavior. Such investigations are necessary to facilitate the customized development of (health) foods with desirable functionalities and qualities. In this context, Mohamad et al. [88] quantitatively analyzed the distribution and stability of β-carotene in a whey protein emulsion during storage by means of in situ Raman microspectroscopy. Similar works are required to design effective emulsion delivery systems for the encapsulation and stabilization of other active substances. In addition, more realistic models (e.g., cell, animal, and human models) must be established to accurately track the interactions between biological macromolecules and the physiological changes taking place in the active substances transported by different types of emulsions [89]. It is also necessary to identify the related problems and limitations of such systems and, ultimately, to develop optimized formulations. The realization of this goal is of great significance for the rational design and safe application of functional foods with high bioavailabilities. Furthermore, Tan et al. [90] demonstrated that nanoemulsions were more beneficial than other emulsions for absorption, according to the standardized gastrointestinal model (INFOGEST). More specifically, they found that the oil droplet concentration affected the bioavailability of β-carotene by altering its digestion, solubilization, and precipitation processes [91]. However, the chemical stability of curcumin decreased upon decreasing oil droplet size, which suggested that the stabilizing effect of microemulsions exceeded that of nanoemulsions [92]. Moreover, Silva et al. [93] used potato starch (gelatinized or natural) as the main component and a low concentration of sodium alginate and gelatin as the continuous phase to reveal that the swelling behavior of the corresponding emulsion-filled hydrogel during digestion was related to its mechanochemical properties. More specifically, during enterolysis, the oil droplets that had been exposed to the sodium alginate–gelatin mixture formed a porous network, whereas the non-gelatinized starch–sodium, alginate–gelatin hydrogels maintained a closed network with no pores. These findings point to the potential utility of starch-filled hydrogels for the delivery of nutritional supplements in the intestinal digestion phase. Thus, research is also required into the health effects of the delivered bioactive substances and the effects of such systems on the intestinal microflora and the health of the host [94]. 

### 2.3. Assisted Extraction and Separation

To assist in the extraction and separation processes, it is often possible to solubilize substances by exploiting the property differences between the dispersed and continuous phases. Emulsions, as separation media, possess numerous unique properties such as a spherical or discontinuous nanoscale structure and a dynamic structure for rapid polymerization, reseparation, and solubilization processes. The microenvironments of W/O emulsions resemble those of cells, and the extracted materials are difficult to denature. Thus, such emulsions are widely used for the extraction of proteins, peptides, amino acids, enzymes, and other bioactive substances. In one study, Zhao et al. [95] compared the functional, nutritional, and flavor characteristics of soybean protein prepared by conventional and reverse micelle-assisted extraction, revealing that the protein extracted by the latter method exhibited an improved nitrogen solubility index, oil absorption, foaming ability, foaming stability, emulsifying ability, emulsifying stability, and nutritional and flavor characteristics.

Similar studies have analyzed and compared the interface structure and volatile flavor substances of peanut protein prepared by buffer extraction and reverse micellar extraction. More specifically, it was found that reverse micellar extraction can alter the surface morphology and composition of peanut protein to enhance its flavor characteristics [96]. In addition, the non-ionic surfactant cloud point has been used to extract chlorophyll molecules from spinach to avoid the use of volatile organic solvents and significantly improve the extraction rate and antioxidant activity [97]. The presented results demonstrated that aqueous solutions of non-ionic surfactants hold great promise for the extraction of highly hydrophobic compounds from biomass, and their direct use in cosmetic and nutritional applications should also be possible without additional recovery or purification steps.

Microemulsions are typically transparent, uniform, isotropic, and thermodynamically stable colloid systems that form spontaneously in the presence of an ultra-low oil/water interfacial tension. The structures of microemulsions are known to benefit the extraction, adsorption, and concentration of target substances during sample pretreatment, and the above steps can be combined with chromatographic techniques, such as thin-layer chromatography, liquid chromatography, gas chromatography, and electrokinetic chromatography, capillary electrophoresis, and mass spectrometry; these combined systems can be referred to as microemulsion chromatography systems [98]. When a microemulsion is used as the mobile phase during microemulsion chromatography, the solute may be distributed between the stationary phase, the continuous water phase, the oil core phase, and the interfacial membrane phase. The operating variables related to the microemulsion mobile phase can be adjusted to change the retention behavior and improve the chromatographic separation efficiency and selectivity. In view of the enriching and solubilizing effects of microemulsions and the reduced interfacial tension, molecules with a high polarity are distributed in the continuous aqueous phase, while molecules with a low polarity are distributed in the oil nuclei or at the interpenetrating membrane fence composed of surfactants and cosurfactants. Due to a range of distribution, adsorption, static electricity, hydrophobic, stereo, and other possible effects, the tested components dispersed in microemulsions can exhibit different migration speeds, which can lead to a high selectivity and an excellent degree of enrichment, ultimately resulting in the sensitive detection and efficient separation of various (e.g., hydrophilic, hydrophobic, acidic, alkaline, and neutral) substances. This method also allows the selective separation of charged and noncharged components to enable the partitioning of complex species that possess only slight differences in their structures and properties.

## 3. Applications on the Interface Structure Scale

The interface between the dispersed phase and the continuous phase acts as a barrier and ensures continuity. From the viewpoint of the interface structure and functions, the current applications are based on the construction of emulsion-based enzymatic reaction plants relying on mass transfer kinetics, as exemplified by the use of protein colloidal particles to build a Pickering interface catalytic platform for regulating the reaction rate and products. In addition, in the body, the interfacial structure can delay lipid digestion, control lipid digestion and absorption, and alter the lipid/protein digestion dynamics.

### 3.1. Biphasic Enzymatic Catalysis Systems

Enzymes catalyze many important reactions with high chemo-, regio-, and stereoselectivities. However, such reactions are usually difficult to carry out in biphasic environments. To improve the efficiencies of enzymatic reactions, stabilization of the enzymes at the water/oil interface can be achieved through emulsion formation, which also increases the interface area. In this context, Zhang et al. [99] enzymatically modified pectin in an aqueous/organic biphasic system by grafting salicylic acid (and their isomers) onto pectin molecules to endow them with good antioxidant, emulsifying, and antibacterial activities. Interfacial engineering in Pickering emulsion photocatalytic microreactors has also been demonstrated to yield several advantages, such as the avoidance of particle aggregation, an increase in the specific surface area, and a uniformization of the active sites [100].

To reduce the costs of such enzymatic reactions, demulsification must be performed under relatively mild conditions to recover the enzymes and separate the products. Although amphiphilic molecules are commonly employed to stabilize emulsions, such molecules affect the enzyme activity. Consequently, Pickering emulsions are widely used in catalysis, as they do not affect the enzyme activity; do not induce pollution; and offer the benefits of a facile purification protocol, a high stability, and a large oil/water interface. As such, Pickering emulsions show great potential for application in the development of bipolar enzyme catalytic bioreactors. The use of a mesoporous carbon-immobilized enzyme (i.e., lipase) as both the emulsifier and the catalyst to prepare a green and efficient Pickering emulsion-based reaction system in a one-step process has also been reported, and the emulsion stability was improved without any reduction in the enzyme activity [101]. It was revealed that, in the Pickering emulsion microenvironment, lipase exerted its catalytic effect via the interface activation mechanism, and a sustainable and efficient enzyme reaction factory composed of numerous emulsion droplets was formed. Subsequently, the same group developed a green and highly active microarray enzyme factory, revealing the dual interface activation mechanism of lipase under ultrasonic and emulsion microenvironments, while also achieving a high enzymatic activity, a good stability, an acceptable recyclability and reusability, and an easily scaled-up protocol [102]. The developed green, solvent-free, and efficient catalyst was therefore considered to have significant potential for use in the enzymatic preparation of food lipids. The above enzyme microarray technology can also be widely used for the green and efficient preparation and enzymatic modification of functional lipids, such as sterol esters, vitamin esters, breast milk structural lipids, and resveratrol ester derivatives, thereby providing a new means for improving the physicochemical properties of natural active substances, enhancing their functional activities, and expanding their scope of application [103].

The design and preparation of amphiphilic nanoparticles and the development of novel stimuli-responsive Pickering emulsion systems can be used to increase catalyst separation efficiencies and simplify catalyst recovery [104]. In this context, Xi et al. employed intelligent self-assembled protein colloidal particles [105] and natural sodium caseinate [106] as biomimetic catalysts for cascade reactions at the oil/water interface to assist the production of foods and pharmaceuticals. It was found that pH-responsive Pickering emulsions could sustain >100 emulsification/demulsification cycles, thereby enabling green and sustainable catalytic reactions to be carried out, followed by facile product separation to reduce the time and costs associated with catalyst separation and recovery. Given their excellent engineering versatilities, the above systems can be widely used in heterogeneous catalysis, food production, crude oil recovery, and transportation applications.

The industrial use of Pickering emulsions in biphasic catalysis is limited by their low oil/water volume ratio and their instability. Therefore, the development of Pickering interface biocatalytic systems with high oil/water ratios, high stabilities, and good recyclabilities is a key research aim. In this context, Wang et al. [107] used HIPEs stabilized by enzyme-modified copolymer nanoparticles to produce a two-phase enzymatic catalysis system featuring an optimal balance of flux, stability, and recyclability. In addition, Jiao et al. [108] developed an enzyme microreactor based on an HIPE-containing monolithic column for protein enzymolysis, wherein they achieved a high enzymolytic activity and a good stability. In contrast to conventional immobilized enzyme reactors based on a particle accumulation structure, the open pore structure of the above reactor effectively improved the protein digestion efficiency because of an improved mass transfer and provided a new strategy for the efficient enzymatic hydrolysis of proteins.

### 3.2. Digestive Effects of Various Substances in the Body

Lipid digestion in food emulsion systems is usually an interfacial process that is mainly influenced by the combination of lipase–biosurfactant (bile salt) complexes with the surfaces of emulsified lipid droplets. Indeed, a number of studies have demonstrated that the structural characteristics of interfacial materials, interfacial film types [109], and interfacial compositions [110] are closely related to the digestive characteristics of emulsions. More specifically, variations in the cellulose lengths [111], types of cellulose [112], and rigidities of the whey protein microgels [113] can modulate the gastrointestinal digestion behaviors of such emulsions. Therefore, the interfacial structures and properties of emulsions that can affect digestion and absorption in vivo are mainly optimized with the aims to regulate lipid digestion, reduce/delay fat absorption, and increase the bioavailability/targeted delivery and release of bioactive substances. Indeed, such strategies are important to improve the nutritional values of food products and to prevent obesity [114]. In this context, it has been reported that the high desorption energy of particles at the Pickering emulsion interface has significant potential for controlling lipid digestion [115]. Therefore, regulation of the rate and degree of lipid and functional factor digestion via interface engineering is a matter of high significance.

In addition, Naso et al. [116] found that food emulsifiers can bind bile salts and influence their structures to control lipid digestion, while Sarkar et al. [117] pointed out that the construction of an oil–water interface prevented the competitive replacement of bile salts and delayed the transport of lipases to lipid substrates to regulate lipolysis in humans. Furthermore, they classified Pickering particles according to their shapes and their enzyme responsiveness properties to explain the behavior and mechanism of stable droplet lipid digestion. They pointed out that control over interfacial particle spacing or adaptability to intestinal biosurfactant desorption can be used to modulate lipid digestion kinetics. Moreover, Chen et al. [118] investigated the enzymatic degradation and bioaccessibility properties of a nanoemulsion featuring β-carotene coated with whey protein isolate (WPI), soybean protein isolate (SPI), and sodium caseinate (SC) during in vitro gastrointestinal digestion. It was found that the WPI- and SC-coated samples rapidly adsorbed the lipolysis products and bile salts at the oil–water interface, preventing lipase from approaching the lipid core and thereby reducing the lipolysis and micellization rates. In the SPI emulsion sample, the adsorption and replacement rates of the bile salts were lower, but the adsorption and replacement degrees were higher, which ultimately resulted in a greater number of surface binding sites for the enzyme and accelerated the lipolysis reaction. 

Similar studies probed the relationship between the digestion behaviors of interfacial proteins and the bioaccessibilities of the lipophilic emulsion constituents, providing guidance for the design of safe protein-based systems for the delivery of emulsified bioactive molecules. In this context, Zhou et al. [119] exploited the special affinity between procyanidins and proline-rich gliadin to design an emulsion interface and develop an antioxidant Pickering emulsion with digestive resistance. The decrease in the content of released free fatty acids and the inhibition of lipid oxidation indicated that interface structure engineering helps to prevent obesity. In addition, Zhao et al. [120] demonstrated that partial enzymatic hydrolysis can be used to prepare functional soybean protein-based nanoparticles suitable for designing particle-carrying interfaces and delaying the digestibility of lipids in emulsion-based functional foods. Furthermore, Wen et al. [121] deciphered the structural network that endows HIPEs with their stability (i.e., through the formation of a crosslinked soy protein microgel) and their in vitro digestibility. It was demonstrated that the HIPE digestibility was affected by the protein concentration, and the release rate of free fatty acids was slower in the case of intestinal digestion.

Future investigations into interface design parameters and the development of new mathematical models should aid the customization of granular interfaces to delay lipid digestion and achieve the site-dependent controlled release of lipid active molecules in composite soft substance systems. In this case, Zhu et al. [122] reported that lecithin can alleviate protein flocculation and promote fat digestion in an infant formula milk model, thereby mitigating the insufficient supply of fat in such milk powders. Similarly, Liang et al. [123] studied the effects of the dairy emulsifier type and the fat droplet size on the gastrointestinal digestive behavior of a model emulsion; these studies provided valuable information for the optimization of infant formula products and nutritional dairy beverages.

It has also been demonstrated that the gastrointestinal digestion behaviors of lipids in crystalline emulsions can be included by changes in the crystal shape [124], the sizes of the fat crystals [125], and the distribution sites of the crystallizable emulsifiers [126]. Moreover, the crystal structure formed by gel self-assembly can lead to only a small area being available for lipase adsorption, thereby indicating that the development of oil–gel emulsions with different physical properties can result in a favorable lipid bioavailability. Combination with a gel structure may therefore prolong the digestion time in the gastrointestinal tract and achieve continuous release. Thus, the design of an oil–gel system with a controllable lipid digestibility that permits control over the bioavailability of the delivered fat-soluble active substance is a matter of high practical significance [127].

### 3.3. Effects of Washing and Disinfection

During the washing of fresh food, surfactant molecules are adsorbed around stains, and so the washing effect is closely related to the interface structure. Given the increased incidence of foodborne diseases related to freshly cut products, the food industry requires novel chemical disinfectants to replace chlorine. In this context, Kang et al. [128] studied the influence of the surfactant type on the washing performance of a cinnamon leaf essential oil emulsion for kale leaves and revealed that this effect was strongly influenced by the ionic properties of the surfactant. Importantly, it should be noted that substances capable of replacing detergents have potential applications in the context of food-grade Pickering emulsions.

### 3.4. Research Prospects for Interface Applications

**Figure 3:**
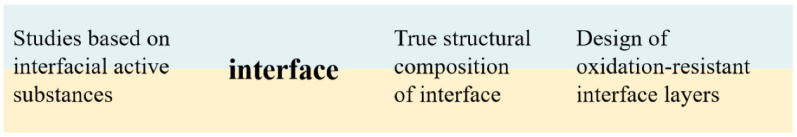
Research prospects for interface applications.

The above discussion suggests that applications based on the interface structure are affected by the structure and function of the emulsifying stabilizer, the interactions between biomolecules and the interface, various colloid phenomena (e.g., miscibility, thermodynamic incompatibility, complexation, flocculation, and separation); the true structural composition of the interface; and the oxidation resistance of the interface layer; among other factors (Figure 3). More specifically, interfacial active substances play a key role in the formation and stability of emulsion systems, as well as in the regulation of their properties and functions.

#### 3.4.1. Studies Based on Interfacial Active Substances

The formation of emulsions and their resulting stabilities, properties, and functions is closely related to the presence of interfacial active substances. For example, milk is stabilized by casein micelles, which possess an irreversible association complex structure that is generated by the bridging of colloidal calcium phosphate. Consequently, milk is extremely stable and retains its structure both at high temperatures and during homogenization, thereby allowing the production of dry milk powder that can be later rehydrated to afford liquid milk. The corresponding dispersion is mainly stabilized by the steric hindrance and electrostatic repulsion effects attributed to casein. These observations indicate the importance of selecting and designing suitable biological surfactants for the development of simple and rapid preparation and control methods to regulate the formation, stabilities, and physicochemical properties of emulsions. 

**Figure 4:**
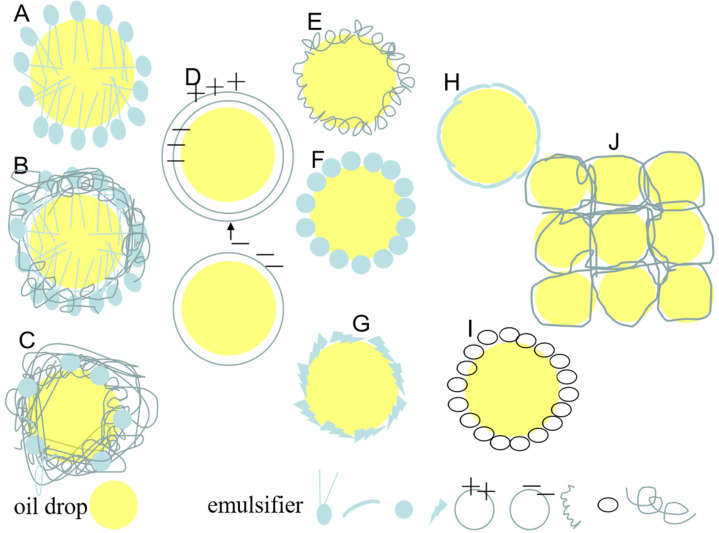
Schematic diagram of emulsifying stabilizer in emulsion: (**A**) surfactant, (**B**) mixed emulsifier, (**C**) conjugated emulsifier, (**D**) multi-layer emulsifier, (**E**) fibers, (**F**) spherical, (**G**) microgels, (**H**) nanogels fibrils, (**I**) hollow nanoparticles, and (**J**) structural agents.

According to report, emulsion stabilization mechanisms mainly include the following three types: (1) Traditional stabilizers are known to create interfacial films coating around the droplets due to molecular rearrangement and relocation, and excess emulsifier in the continuous phase also provide steric hindrance. (2) Pickering particles that effectively adsorb and irreversibly anchor to the water–oil interface to form a solid particle protective coating around droplets and create steric hindrance by interior particle networking in the continuous phase, the analytical energy is much greater than the adsorption energy. (3) The structuring agent mainly refers to the material unable to adsorb onto interface but rather relies on matrix formation, which enhance the 3D reticular structure in the bulk, which are advantageous to bridge, connect, and immobilization the different droplets more compactly. According to their action mechanism, the emulsifying stabilizers can be divided into traditional emulsifiers (mainly relying on good amphiphilic properties) (such as surfactant, mixed emulsifier, conjugated emulsifier, and multi-layer emulsifier) (Figure 4A–D) [17,20,21,24]; Pickering-type emulsifiers (mainly relying on the partial wettability of particles and relatively structural integrity to achieve the irreversible interface adsorption) (such as micro/nanoparticles, which can exist in various forms, e.g., fibers, spherical, microgels, nanogels fibrils, and hollow nanoparticles) (Figure 4E–I) [13,33,35,39,43,44,53]; and structural agents (Figure 4J). Moreover, irrespective of their type, emulsifying stabilizers should exhibit appropriate particle sizes, morphological characteristics, and amphiphilicity to ensure that they stabilize the internal phase and help to preserve its structural stability in the continuous phase [4].

Researchers have focused on the different structural and functional criteria required by different emulsion systems. For example, the amphiphilicity, molecular size, and adsorption speed of the interfacial active substances for conventional emulsions have been investigated, while, for Pickering emulsions, the importance of the three-phase antennae and the deformation recovery of particles at the interface have been examined. To date, research in this area has focused on the development of natural green material sources [129]; structural and functional property analyses; molecular self-assembly; molecular modification; molecular combinations and interactions (i.e., direct mixing, layer-by-layer assembly, and conjugated composite formation); simple and efficient preparation/modification methods; the exploration of structure–activity relationships and stability mechanisms; and the development of antioxidant and stimuli-responsive interfacial active materials [130]. 

As previously described, stimuli-responsive interfacial active substances are reassembled upon changes in the external environment [131,132,133]. Therefore, stimuli-responsive Pickering emulsions, such as temperature-responsive Pickering emulsions, photoresponsive structured liquids [134], thermoresponsive nanoemulsions [135], and Janus nanoparticles [136], can be obtained by changing the surface states of the amphiphilic nanoparticles (such as the charge and wettability) using environmental stimuli. It should be noted that to obtain functional Pickering emulsions for targeted release, trigger release, switch release, or light-triggered molecule selective release, the precise assembly of carrier systems is required to prevent unnecessary leakage; to obtain multiple stimuli-responsive surfactants; and to build dynamically reconfigurable, movable, and controllable multifunctional droplets [137]. For example, Zhong et al. [138] prepared a soybean lipophilic protein–hydroxypropyl, methylcellulose–calcium chloride thermosensitive emulsion gel and demonstrated that the addition of a salt changed the gelation structure of the emulsion and effectively reduced its temperature. This thermosensitive emulsion gel could better adapt to the human body temperature and transform from an emulsion to an emulsion gel in the digestive tract, thereby contributing to the targeted release of nutrients and controlled lipid release. Inspired by cell phagocytosis, Rodríguez-Arco et al. [139] prepared a self-driven magnetic Pickering emulsion (MPE) capable of selectively absorbing silica gel particles. After ingesting colloidal particles, the water-soluble carrier was selectively transported and released, while the enzyme activity was coupled within the MPE droplets. Their results provide insights into the development of colloid-based materials and provide a new method for the micron-scale regulation of particle synthesis to induce highly ordered behavior. These explorations are expected to bring new opportunities in the fields of sensing, smart packaging, and drug delivery [137]. The ongoing research leaves no doubt that attractive smart materials based on stimuli-responsive emulsions will be available in the future [140].

#### 3.4.2. The True Structural Composition of the Interface

Conventional emulsions are mainly stabilized by traditional emulsifiers, while Pickering emulsions are usually stabilized by colloidal particles. However, food emulsions possess complex interfacial compositions and usually contain both emulsifiers and particles, i.e., they can be classified as particle–polymer, particle–surfactant, or particle–polymer–surfactant emulsions. Given the different molecular structures, charge characteristics, addition orders, and mass ratios of the emulsifiers and particles, the resulting mixed interfaces can exhibit diverse microstructures, which can be divided into those prepared by co-adsorption, complexation, or layer-by-layer deposition. Accordingly, the interfacial behaviors of mixed interfacial active substances and their abilities to regulate the emulsion stability, functionality, lipolysis, digestion, and absorption properties are also variable [141,142]. Thus, the detailed exploration of this area is of great significance in the context of future applied research.

Zhang et al. [143] revealed the existence of synergistic and competitive adsorption between gelatin and surfactants with different molecular structures at the oil–water interface of fish oil-loaded emulsions and demonstrated their effects on the emulsion performance. In addition, Wei et al. [144] found that compared with emulsions that were stabilized by particles alone, emulsions stabilized by particles and emulsifiers exhibited a higher β-carotene entrapment efficiency under environmental pressure. The same group further studied the advantages of nanoparticles (zein colloid particles), biopolymers (propylene glycol alginate), and surfactants (rhamnolipid) in terms of enhancing the stabilities of emulsions and functional factors and regulating their digestibility and bioavailability properties [145]. Furthermore, Zheng et al. [146] reported that the lipolysis of protein-stabilized emulsions was inhibited by the addition of different low-molecular-weight emulsifiers. By monitoring the interactions between droplets, the authors found that the active components and surfactant molecules exhibited competitive adsorption in the same phase but showed mixed adsorption when present as different phases. The above studies are therefore expected to contribute to the design of slimming foods based on bioactive ingredients that are intended to increase the satiety properties and nutritional values of such foods.

#### 3.4.3. Design of Oxidation-Resistant Interface Layers

The emulsion structure design is important for improving the oxidation stability of lipids, since different types of emulsions (e.g., conventional, multilayer, gel, and Pickering emulsions) are known to exhibit varying oxidation stabilities. More specifically, the oil-in-water interface structure, where the oxidation reaction begins and propagates, is a key influencing factor, and so the design of antioxidant interface layers can improve the oxidation stabilities of food emulsions. As a result, the sensory nutritional characteristics of these products are enhanced, and their shelf lives are lengthened.

The surface area properties that can be used to control lipid oxidation include the interface thickness, the interface filler and permeability, the interface composition [147], the interface charge, and the interface structure. Moreover, adjustment of the emulsion structure is a feasible and superior method to improve the oxidation resistance. Compared with conventional emulsions, multilayer emulsions feature a thicker interfacial layer that acts as a physicochemical barrier [148], while the high viscosity of the continuous phase in a gel emulsion hinders the transfer phenomena from taking place. In addition, Song et al. [149] investigated the effects of the interface thickness and the droplet density on the chemical stability of β-carotene in emulsions and on the ability of oil-soluble antioxidants to retard β-carotene degradation; they found that thick and dense interfacial layers could effectively delay carotene degradation. Furthermore, the water–oil interfaces of oil-in-water Pickering emulsions are covered by dense granular layers, which reduce the probability of contact between oil and oxygen, the co-oxidant (metal ions), light, and other pro-oxidant factors, thereby enhancing the oxidation stability of the oil [150]. It was also found that the generated particles could carry antioxidants to the emulsion surface, thereby further enhancing emulsion antioxidant properties [151]. To date, extensive studies have been carried out based on the advantages of bio-based antioxidative emulsifiers [152], including antioxidant peptides [153], protein–polysaccharides [154], protein–polyphenols [155], polyphenol–protein–polysaccharide complexes, the Maillard reaction conjugates of protein hydrolysates and polysaccharides, and Maillard reaction products [156]. 

In addition, the development and utilization of antioxidant emulsifiers, couplings, and compounds can be used to inhibit oxidation and promote the application and development of antioxidant functional foods. Recently, Bravo-Díaz [157] reviewed kinetic approaches to the control of lipid peroxidation in oil-in-water emulsions. Moreover, it has been reported that the antioxidant effects of emulsifiers and other substances adsorbed on the droplet surfaces are exerted by various means, including chelation, free radical scavenging, the binding of secondary lipid oxidation products, and physical barrier formation [158]. Furthermore, the effects of the emulsifier micelle concentration and the mixing mode of the antioxidants on the antioxidant efficiencies of emulsions can be used to optimize emulsion formulae [159]. Plant polyphenols tend to exhibit an excellent antioxidant activity and are widely used to inhibit lipid oxidation in Pickering emulsions. The formation of dense interfacial layers through the complexation of polyphenols by proteins or polysaccharides can also enhance the antioxidant capacity. In this context, Yi et al. [160] used natural antioxidants (i.e., black rice anthocyanins) to inhibit lipid and protein oxidation in whey protein-stabilized emulsions and thereby improve the overall oxidation stability. Moreover, co-encapsulated resveratrol and epigallocatechin-3-gallate were found to improve the antioxidation properties of fish oil emulsions [161]. From the aforementioned reports, it is clear that the antioxidant activities, distributions, and interactions of phenolic compounds at the oil–water interfaces of food emulsions should be examined in detail.

Wang et al. [162] reported the design of Gemini interface antioxidants based on gallic acid as a model plant polyphenol, and they revealed the excellent interfacial colonization ability and antioxidant activity of dodecyl Gemini gallic acid in the complex phase of emulsions. As a result, they were able to overcome the shortcomings exhibited by the intermolecular assembly of traditional surfactants. It should be noted that the extraordinary antioxidant activity of Gemini antioxidants stems from their interfacial self-assembly behaviors, thereby indicating their potential application as emulsifiers and carriers for the controlled release of genes and drug molecules in various industries. Similarly, by tuning the self-assembly of amphiphilic sodium alginate-decorated selenium nanoparticles, surfactants can be used to prepare antioxidant Pickering emulsions [163].

Another relatively recent report found that the biosurfactants produced by lactobacilli can act as antioxidants and biopreservatives to improve the shelf life of raw ground goat meat [164]. It has also been reported that natural particles (i.e., matcha raw flour, spinach leaves, pineapple fibers, rosemary cake, turmeric, and carrot extract powder) can also protect emulsions from lipid oxidation and coalescence [165]. This behavior may be due to the accumulation of chain-breaking antioxidants and/or chelating agents that are embedded in the particle matrix on the droplet surface but can still be used to react or interact with pre-oxidation catalysts.

## 4. Applications on the Macrostructure Scale

### 4.1. Design of Structured Soft Materials for Food Applications

**Figure 5:**
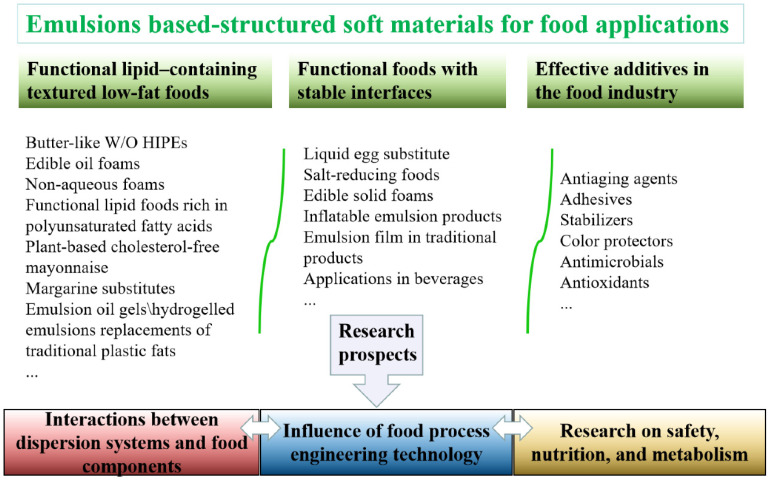
The key food-grade emulsion-based structured soft materials for food applications and their future research prospects.

The applications of emulsions in foods are fully reflected in the design of structured soft materials for low-fat foods and functional foods with stable interfaces (Figure 5). Overall, such applications involve control over the texture, taste, and appearance of the food.

#### 4.1.1. Development of Low-Fat Foods

The pursuit of foods containing health lipids is mainly reflected in the development of low-fat foods, the replacement of foods that may contain *trans*-fatty acids, increases in the unsaturated fat contents in food systems, and reductions in the saturated fat contents. In this context, the use of functional lipid-containing textured foods instead of hydrogenated oils is the preferred choice. Thus, emulsion-based food systems are of great interest because of their value in developing healthy lipid foods that can satisfy the nutrition, taste, texture, and satiety requirements desired by consumers. For example, water-in-oil HIPEs offer a favorable texture and facile usability, while edible oil foams can be used to develop low-fat foods. More specifically, nonaqueous foams formed by oil–gel stirring can be used to develop healthy foods with low fat and saturated fatty acid contents, as well as a desirable taste and texture. To date, structured oils, HIPEs, emulsion gels, and oil gels have attracted significant attention due to their semi-solid or solid textural properties. These systems can replace saturated fats, such as hydrogenated vegetable oils, and can be used to effectively increase the contents of unsaturated fats in various foods. Moreover, the use of double emulsions based on the microstructural binding of water is also a promising fat replacement strategy [166]. 

Lee et al. [167] developed a highly stable W/O HIPE that consists of 80% water and 20% milk fat droplets and mimics the color and texture of butter while serving as a low-calorie, butter-like spread with a low-fat content. The stability, viscoelasticity, and rheological properties of this HIPE can be further improved by adding carrageenan and beeswax, and the addition of milk proteins, plant-derived proteins, vitamins, and flavoring substances to the internal water phase can be carried out to modify the product flavor, taste, and health benefits.

The potential benefits of edible oil foams and the recent advances in their research have been described by Heymans et al. [168], who also considered the Pickering stability of crystalline particles and the influence of food processing on their crystal properties. In addition, Li et al. [169] found that medium-long-chain diacylglycerol (MLCD) can undergo interfacial crystallization after emulsification and cooling, thereby greatly improving the physical stability of the corresponding Pickering emulsion during storage or freeze–thaw cycling. Researchers also replaced partially hydrogenated palm oil with MLCD to prepare an oil–gel-based non-aqueous foam with a good storage stability, and they demonstrated the suitability of this foam for use in the development of low-calorie health foods with a desirable texture and taste [170]. Furthermore, researchers used the self-assembly and co-crystallization between MLCD and β-sitosterol to form a dense crystal network, which was used to prepare new emulsions for controlling the release of volatile compounds. Using this system, they also further prepared rigid and stable oil gels and nonaqueous foams to promote the development of healthy foods with desirable textures [171]. Similarly, the above authors found that the synergistic action of diacylglycerol and polyglycerol polyricinoleate (PGPR) can be used to prepare a water-in-oil emulsion with a high hardness value, a high viscoelasticity, and an excellent freeze–thaw stability [172]. These results have important theoretical and practical significance for reducing the use of traditional saturated hydrogenated fats and constructing novel food systems based on a healthy oil that is free from saturated *trans*-fats.

Emulsion gels are semi-solid food systems with a gel network structure that combine the properties of emulsions and gels to improve the stability of the mixed emulsion system while also enhancing the rheological properties of the gel. Many foods, including fatty puddings, yogurts, salad dressings, sausages, tofu, and fresh cheese, are emulsion-filled gels. More specifically, HIPEs are typical emulsion–gel systems that contain a high proportion of the dispersed phase and are highly stable. To generate stable emulsion–gel systems, heating, acid treatment, and enzyme treatments can be used to induce crosslinking of the protein matrix and generate a spatial network structure. In addition, cooling and the introduction of metal ions can produce an emulsion–gel network structure from a polysaccharide matrix. The structural and functional properties of the resulting systems can impart oils with the functionalities of solid fats, which is conducive to the development of a wide variety of structural lipids and semi-solid foods, in addition to promoting fat replacement in various foods, as described below.

(1) Functional lipid foods rich in polyunsaturated fatty acids: By constructing and assembling soybean β-conglobulin–polyphenol composite nanoparticles, Tang successfully prepared linseed oil-based HIPEs that exhibited an excellent oxidation stability. These HIPEs exerted an excellent thermal protection effect on β-carotene that was loaded onto flaxseed oil, and they significantly inhibited oxidation of the flaxseed oil [173]. Similarly, the wrapping of oil droplets in a three-dimensional solid gel-phase network (i.e., a hydrogel) has been found to greatly improve the emulsion stability and enhance the mechanical properties of protein hydrogels. This was achieved through the formation of strong interactions between the proteins adsorbed on the oil droplet surfaces and the proteins present in the gel matrix. For example, crosslinking with genipin can enhance the gel properties of hemp seed protein and improve the textures of emulsion-filled products, wherein the degree of crosslinking can be tuned to control product digestibility [174]. Considering that flaxseed oil is rich in polyunsaturated fatty acids, the above hydrogel may provide new opportunities for the development of functional foods.

(2) Plant-based cholesterol-free mayonnaise: An HIPE system similar to mayonnaise was prepared using plant-based emulsifiers instead of egg yolk to inspire the development and utilization of egg-free mayonnaise [175]. The mayonnaise substitute prepared using wheat gluten HIPEs was similar to mayonnaise in terms of its appearance, microstructure, rheological behavior, and oral friction properties [176]. It has also been reported that HIPEs based on citrus fiber and corn polypeptides can also be used as mayonnaise substitutes and that they exhibit an excellent thixotropic recovery, as well as a good thermal and freeze–thaw stability. As a result, such systems have potential for application in the preparation of new pasteurized sauces and condiments with long shelf lives [177]. Similarly, Lu et al. [178] used a Pickering emulsion constructed from ultra-fine apple peel powder to prepare cholesterol-free mayonnaise as an alternative to traditional mayonnaise, and they demonstrated that the obtained product exhibited excellent nutritional and physicochemical properties, as well as a good stability. In addition, Pickering emulsions stabilized with chitosan–stearic acid nanogels, further incorporating clove essential oil, were used to produce fish oil-enriched mayonnaise with a more gelatinous structure and a good oxidation stability [179]. However, further research is needed to confirm the effects of other food ingredients in the commercial mayonnaise recipe [180].

(3) Margarine substitutes: Using peanut protein microgel particles as an emulsifier, Jiao et al. [181] developed new types of HIPEs. As these systems did not contain *trans*-fats, and since they resembled margarine in terms of their external morphologies, rheological behaviors, and other functional properties, they were considered to be potential margarine substitutes. Such systems are of importance since they can help reduce the risk of developing cardiovascular disease, diabetes, and cancers caused by *trans*-fats.

(4) Oil gels/hydrogel emulsions: The use of biopolymers to construct new oils with zero *trans-* and saturated fatty acid contents is of great significance in terms of improving the nutritional values and health profiles of fat-based foods. Example systems include emulsion-based oil gels [182] and hydrogel emulsions [183]. In addition, Wang et al. [184] constructed a camellia seed oil–gel system loaded with SPI nanoparticles using an emulsion template method, while Pan et al. [185] reported the xanthan gum-assisted fabrication of a stable gelatin–procyanidin emulsion-based oil–gel and successfully applied it to pastry to delay oxidation. Furthermore, Yang et al. [186] recently demonstrated that egg white protein particles and rhamnolipid-based emulsion gels could substitute butter in the preparation of cookies, and they found that the appearance, texture, and taste of the cookies were improved. Moreover, a gel prepared using whey protein and sodium dodecyl sulfate was employed as a fat substitute in low-fat sausages to improve their water retention capability, emulsion stability, and texture properties [187], while a phenolic compounds-supplemented emulsified gel was used as a substitute for animal fat in Frankfurt sausages to increase their oxidative stability during cold storage [188]. Importantly, no adverse effects were detected in relation to the sensory/physicochemical properties or the fat structure of these Frankfurt sausages. Moreover, the good thermal and storage stabilities of the sausages indicate the potential of such systems for helping to meet the demand for high-quality healthier products.

As mentioned above, emulsion gels have been widely used to develop healthy meat products [189], reduce the levels of *trans*-fats, and impart greater stabilities to the food structures and to heat-sensitive ingredients. However, as the abovementioned HIPEs are not suitable for direct consumption because of their high oil contents, researchers have developed low-oil-phase emulsion gels, such as emulsion filling gels [190], emulsion fluid gels, and emulsion granular gels, to reduce the oil contents and broaden the application scope of emulsion gels. For example, Hu et al. [191] prepared a defatted Antarctic krill protein-stabilized low-oil emulsion gel using high-intensity ultrasonication. They found that the stability of this gel could be attributed to the steric hindrance and hydrophobic interactions between the constituent particles, and the potential value of this gel was demonstrated for food, nutrition, pharmaceutical, and cosmetic applications. Moreover, upon the addition of curcumin, the antioxidant properties of the low-oil-phase emulsion gel foods were enhanced, and the lipid-soluble nutrients, which can be easily oxidized in a low-fat diet, were protected [192].

The development of healthy lipid alternatives for foods is full of opportunities and challenges, and so, it is necessary to evaluate the application of mixed fat substitutes in food matrices in terms of their melt and cooling effects; their physicochemical properties (i.e., hardness, texture, crisp, daub, and chewiness); and their nutritional and technological functions. Moreover, fundamental investigations are required to ensure that such systems can mimic the beneficial properties of lipids and to develop cheaper and more facile processes. It should also be noted that the wide range of food systems available to us exhibit a variety of different complexities, and the interactions between different substances in their compositions may affect the final product characteristics. For example, Gutiérrez-Luna et al. [193] reviewed gels as replacements for lipids in baked goods and examined their application effects and nutritional properties and described various challenges faced in the production of nutritionally enhanced foods, particularly those related to their technical and sensory acceptability. In addition, Grossmann et al. [194] recommended a series of standardized methods for testing the quality attributes of plant-based milk and cream substitutes. It is expected that their work will aid the design of plant-based milk substitutes with properties similar to those of real dairy products; this and similar studies are of great significance for promoting the development of fat-based foods exhibiting better health, nutrition, and safety profiles.

#### 4.1.2. Development of Functional Foods with Stable Interfaces

Functional foods with stable interfaces in different textural states can be developed using a selection of food emulsion systems. Currently, emulsion-based functional foods mainly include salt-reducing foods, edible solid foams, inflatable emulsions, edible protein films, and functional drinks. Interestingly, variations in the pH of mung bean protein-based emulsions and the addition of calcium have been demonstrated to produce a texture similar to that of an egg; therefore, these emulsions have potential use as a liquid egg substitute [195].

Due to the significant health risks associated with a high-salt intake, the development of salt-reducing foods has received growing attention [196]. In this context, Wang et al. [197] discussed a promising method for regulating the salinity by reducing the sodium content through the emulsion-based delivery of NaCl. In addition, the emulsion size, the emulsion drying process, the obtained powder form, the oil phase composition, and the positioning of NaCl in the emulsion were identified as promising research directions for the rational design of emulsion systems to achieve sodium reduction. Furthermore, Sun et al. [198] widely discussed a design strategy based on adjusting the structures of foods and salts to achieve salt reduction, and they examined the relationship between salt reduction and the structural characteristics of the emulsion-based products. Compared to single-emulsion systems, double-emulsion systems were found to offer an enhanced sensory perception of salty taste, since the dissolved salt is present in both the internal and external phases. In terms of a cheese matrix, this relatively loose and porous microstructure promotes salt release; however, the release of salt in this matrix can be limited by increasing the gel strength.

The application of edible solid foams with adjustable structures and mechanical properties has attracted attention in the food industry [199]. For example, Zhang et al. [200] prepared an O/W Pickering emulsion stabilized by soy protein isolate/bacterial cellulose, and they produced an edible solid foam with excellent mechanical properties and a good biocompatibility after removing the solvent from the emulsion.

Whipped cream is a typical inflatable emulsion that can be described as a three-phase system composed of water, oil, and air. In this system, a proportion of the agglomerated fat spheres forms a crystalline network to wrap the air present in the system and generate a foam. In this context, Wang et al. [201] found that to meet various production needs, an appropriate emulsifier formula can be selected according to the functional differences between emulsifiers, ultimately leading to the generation of dairy and non-dairy inflatable emulsions with good sensory properties and textures. This synergistic effect between emulsions can lead to superior product qualities, an enhanced foaming rate and foam hardness, an increased viscosity of the inflatable emulsion, and an improved emulsion stability to enhance fat floating.

Heating and drying are the key factors that are known to affect the properties of soybean protein films. More specifically, it has been reported that increasing the heating temperature had no obvious effect on the protein composition of a soy protein isolate–oil emulsion film; however, the glass transition temperature of the protein was increased [202]. Thus, optimization of the production and drying processes allowed the preparation of an edible protein film from soybean protein and soybean oil. This film can potentially replace tofu skin in traditional products, such as rotten skin shrimp rolls, pork rolls, and rice balls.

The application of colloidal emulsions in beverages is also on the rise. For example, due to their favorable taste, flavor, and nutritional value, acidic milk beverages have broad market prospects. However, their processing and storage often induce emulsification and precipitation, which can have detrimental effects on the sensory qualities and the shelf lives of products; this problem can be mitigated through the addition of polysaccharide hydrophilic colloids. More specifically, Guo et al. [203] reviewed the stabilizing effects of polysaccharide macromolecular hydrophilic colloids on acidic milk beverages, in addition to evaluating the functions, influencing factors, regulation methods, and stabilization mechanism of these colloids. Moreover, they reviewed strategies related to the structural modification and functional improvement of polysaccharide stabilizers, revealing the trends and challenges of developing plant protein-based acidic beverages. Furthermore, Du et al. [204] designed a sports and meal substitute drink using a high-energy emulsion containing high oil, protein, and maltodextrin contents. Regulation of the stability, viscosity, and color of protein drinks provides a theoretical basis for the manufacture of such products with different sensory characteristics. Recently, it has been reported that Pickering emulsions based on soy protein isolate–tannic acid can protect aroma compounds in beverages [205], and the preparation of solid drinks (e.g., oil powders) based on the Pickering emulsion template and spray-drying [206] or vacuum freeze-drying [207] is also an important research direction. For example, tea powders are required to have a high water-solubility, a controllable oxidation stability, excellent rehydration properties, and a good fluidity.

#### 4.1.3. Effective Additives in the Food Industry

In view of their favorable functional properties, emulsion systems can be used as ingredients or effective additives in the food industry, wherein they can play a role similar to that of food additives to improve the taste, color, flavor, safety, and shelf life of the final product. Some key examples of such emulsion systems are detailed as follows:

(1) Antiaging agents: Dun et al. [208] investigated the effects of micro- and nanoemulsions on the gelatinization and aging characteristics of rice starch. They found that the addition of emulsions inhibited the short- and long-term aging of rice starch, thereby demonstrating the potential applications of such emulsions in starch foods. Researchers have also developed an antiaging and bacteriostatic edible emulsion film based on mung bean starch and guar gum [209]: the emulsion film prepared using sunflower seed oil improved the quality of rice cakes and inhibited their aging during storage; the emulsion film prepared using grape seed extract was found to exhibit antibacterial activity and was also suitable for application in the rice cake industry.

(2) Adhesives: Pure starch-based adhesives often exhibit poor mechanical properties, water resistance properties, and storage stabilities. To address this, a starch-based adhesive emulsion reinforced by amphiphilic nano-TiO_2_ was examined, and this system was found to induce crosslinking between the latex particles inside the starch adhesive, ultimately enhancing the stability and adhesion properties of the adhesive [210]. Moreover, the crosslinking induced in this system altered the water migration rate and the film formation time during adhesive curing, thereby endowing the starch film with a superior compatibility with food matrices, in addition to a higher strength and an elevated water resistance.

(3) Stabilizers: The quality of chicken sausage is known to depend strongly on the related emulsifying stability. Typically, nonmeat proteins (e.g., whey protein, casein, and soy protein) are added to improve the emulsifying stability of a meat product. However, non-meat proteins can act as allergens. Zhu et al. [211] found that l-arginine/l-lysine could improve the emulsifying stability of chicken sausage by increasing the electrostatic repulsion of emulsion droplets and decreasing the interfacial tension between soybean oil and water, thereby achieving a breakthrough in chicken sausage production without using nonmeat proteins. In addition, Fang et al. [212] found that high-quality golden line surimi gel products could be prepared under the combined action of emulsified lard and transglutaminase, while Xu et al. [213] recently reported that the simultaneous addition of salt and HIPEs stabilized by yolk-modified starch complexes could positively affect the formation of chicken surimi gel; promote the generation of a compact gel network structure; improve the gel properties (e.g., hardness, texture, and viscoelasticity); and reduce losses during cooking, which demonstrated that HIPEs have great potential use as healthier lipid components in meat products.

(4) Color protectors: As reported by Tao et al. [214], steppogenin, vitamins C and E, and butylhydroxytoluene can be used to prepare oil-in-water microemulsions. This microemulsion technology can greatly increase the solubility of steppogenin, reaching values 3000 times greater that in water, and it also provides an effective solution for the inhibition of enzymatic browning of fresh apple juice by flavonoid tyrosinase inhibitors. Furthermore, the addition of antioxidants, such as vitamins C and E and butylhydroxytoluene, also enhances the stability of the juice during storage.

(5) Antimicrobials and antioxidants: It is widely known that the oxidative spoilage of food leads to a deterioration of food quality, and so, the application of nanoemulsion systems that deliver antioxidants and antibacterial agents to replace traditional antioxidants and antibacterial preservatives is of particular interest. In such emulsion-based products, plant essential oils and polyphenols are commonly used as antimicrobial and antioxidant substances, respectively [215]. Feng et al. [216] found that the addition of a vitamin E-containing nanoemulsion to fish sausage could effectively delay the oxidation of fish sausage oil and protect the degradation of the unsaturated fatty acids. The prepared nanoemulsion exhibited a small particle size, a uniform particle size distribution, and an excellent stability, ultimately promoting the antioxidant effect of the encapsulated vitamin E. Moreover, the addition of this nanoemulsion did not affect the texture, color, or other sensory properties of the fish sausage, which is conducive to food industry applications. Similarity, a curcumin and rosemary nanoemulsion was found to be applicable to all perishable fish products examined in a recent study by Ceylan et al. [217], wherein treatment of the fish surface with the nanoemulsion effectively limited the growth of bacteria, thereby inhibiting bacterial spoilage. Furthermore, it was reported that the encapsulation of cinnamon essential oil in a chitosan- and pectin-based nanoemulsion led to a system that greatly improved the water dispersity, thermal and chemical stabilities, bioavailability, and biological activity of the essential oil, whilst also permitting its controlled release to ensure the quality, safety, and nutritional status of the meat slices [218]. Moreover, the combination of low-temperature atmospheric plasma and aromatic alcohol nanoemulsions was demonstrated to have a significant synergistic inhibitory effect on *Escherichia coli* O157:H7 and salmonella in instant chicken [219]. This finding could lead to the improved control of these pathogens in cooked chicken without affecting the degree of oil oxidation, which is an important index for evaluating the meat quality. As a further example, da Rosa et al. [220] used a pluronic surfactant-based nanoprecipitation method to encapsulate oregano and thyme essential oils in zein nanocapsules, and they demonstrated that these heat-resistant nanocapsules could be used as in situ bread preservatives to protect the bread from mold. The potential use of such systems has also been discussed for the delivery of plant essential oils and extracts as preservatives and antioxidants for cheese and cheese products [221]. In this case, the encapsulation of these oils is advantageous over the direct addition approach, since it helps to avoid the flavors and odors associated with the oils. Importantly, encapsulation also prolongs the antibacterial and antioxidant activities and can lead to superior control over moisture and mass losses while improving the shelf life of cheese and enhancing its physicochemical/sensory characteristics. Moreover, nanoemulsions and bionanocomposite membranes have been found to control the oxidation rate and the degree of carbon dioxide exchange in cheese, while also acting as carriers of antimicrobial agents [222]. Such advantages can ultimately reduce weight loss and minimize microbial decay to improve the shelf life of cheese.

### 4.2. Research Prospects for Structured Soft Food Materials

Food is a complex soft substance system with multiple components, scales, and phases. However, when emulsion systems are mixed with food ingredients, the complexity of the macromolecular interactions is affected by additional factors, such as the source of the raw food materials, the processing technology, the food colloid concentration and structural parameters, the pH value and ionic strength of the system, and the temperature. To understand and describe such complex structures, it is necessary to establish relationships between macroscopic functional properties and microscopic structural features. Understanding such correlations is required from the perspective of not only the basic theory but also industrial processing (to address various practical issues). Therefore, to strengthen the application potential of food emulsion systems in real foods such as butter, frozen desserts, fat substitutes, and 3D-printed foods, the practical significance of the structural design must be emphasized, and the qualities of multicomponent emulsion systems should be improved. However, the complexity of the multiscale structure and the wide variation in properties pose great challenges. In general, such issues can be categorized by the areas of food physical science, structural rheology, and colloid science, thereby highlighting the necessity of a creative multidimensional interdisciplinary integration of food science, condensed matter physics, colloid chemistry, and polymer science to improve the overall quality and value of emulsion-supplemented foods.

#### 4.2.1. Interactions between Dispersion Systems and Food Components

The stability and functional properties of emulsions may change upon the mixing of emulsions with other ingredients during food processing and storage. Therefore, the composition, content, and structural characteristics of real emulsion-based food components deserve careful investigation. As an example, under different solute conditions, Crowley et al. [223] investigated the effects of heating and cooling on the colloidal properties of β-casein concentrate for forming protein complexes, and they showed that, in common nutrient product formulations, these properties exhibit significant temperature-dependent changes in the presence of minerals. In addition, Chen et al. [224] found that the physicochemical and interfacial properties of biomimetic milk fat globules (BMFGS) strongly influence the protein/phospholipid ratio in the receptor phase, and so, this factor should be considered in the design and application of BMFGS. Furthermore, Renhe et al. [225] studied the effects of partial whey protein depletion on the thermal stability of condensed milk during membrane filtration. Compared with ultrafiltration, microfiltration reduced the whey protein content to a sufficient level to increase the thermal stability. This difference was attributed to the type and amount of complexes formed in the whey phase. Moreover, Zhou et al. [226] found that the calcium fortification of plant-based milk may reduce the bioavailability of vitamin D due to the formation of insoluble calcium soaps in the small intestine. In this context, it should be noted that, compared with CaCl_2_, CaCO_3_ has a higher calcium bioavailability. These findings therefore contribute to the rational development of nutritionally fortified plant-based milks with improved physicochemical and nutritional properties, and they could also lead to the design of emulsion delivery systems with high vitamin bioaccessibility properties. It has also been reported that variations in the pectin content and the gastric pH affected the stable algal oil emulsion digestion of soybean lecithin, and the presence of a stable emulsion microstructure during gastric digestion improved the lipid digestibility and the docosahexaenoic acid (DHA) bioavailability in vitro [227]. Additionally, Hu et al. [228] reported that the inclusion of biopolymers can improve the bioavailability of hesperidin in emulsion-based delivery systems, mainly because these biopolymers can alter the crystallization behavior and water solubility of the health nutrients.

Since interactions with other components of the food formulation are known to directly determine the sensory quality of the final food product, as well as its processing, nutritional value, and stability, it is important to study the structures of emulsion-based foods at different (e.g., micro-, meso-, and macro-) scales and explore the corresponding structure–activity relationships to regulate the food structure and function. More specifically, the movement of hydrophilic reagents through the oil phase should be investigated, as should the influence of the oil phase composition and the carrier oil and the self-assembly or co-assembly of the multicomponent biomolecules. Furthermore, at high concentrations, the effects of macromolecular crowding on the stabilities of emulsion-based foods and on the conformational structures of the interfacial active substances also deserve attention [229].

Other areas of interest include the interactions of surfactants with proteins and the effects of food-derived mixed protein/lipid/polysaccharide interactions, aggregation, and functional properties on the physicochemical parameters of colloid systems. Moreover, the influence of thickeners, hydrocolloids, mineral ions, pH, and the solute conditions on the colloid properties should be investigated. Indeed, the interactions between emulsion systems and food components are of great significance when considering the structurization of food colloids and the design of future food structures. The use of food colloids to design functional products for patients suffering from dysphagia or diabetes, or for the preparation of botanical artificial meat for vegetarians, is also of particular interest in the current climate.

#### 4.2.2. Influence of the Food Process Engineering Technologies

**Figure 6:**
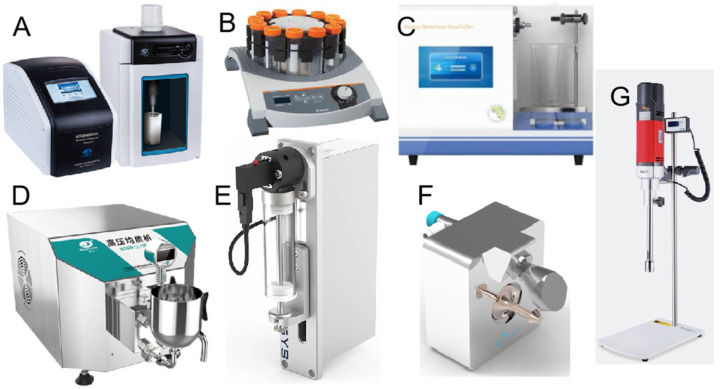
Emulsification techniques instruments: (**A**) ultrasonic processer, (**B**) vortex generator, (**C**) online film emulsifying equipment, (**D**) high-pressure homogenizer, (**E**) microfluidic devices, (**F**) fogging tester, and (**G**) high-speed shearing dispersers.

In general, an emulsion formation process occurs in a high-speed flow state—that is, in a high-energy injection state. The essential process involves realizing the migration and diffusion of interfacial active substances to the surface of the forming dispersed phase under the condition of high energy flow, which then must smoothly adsorb to the surface of the forming dispersed phase, rearrange, crosslink, and, finally, solidify on the forming surface. Notably, a certain energy barrier activation energy must also be overcome, and each step is accompanied by a decrease in the free energy of the system. Thus, emulsion preparation techniques usually involve high shear processes like high-speed shearing dispersion, high pressure homogenization, microfluidics, ultrasound, dual ultrasonic nebulizer spray, vortex, layer-by-layer electrostatic deposition technique, electric field emulsion method, etc. As shown in the Figure 6, the emulsification instruments corresponding to the above emulsification techniques were as follows: ultrasonic processer, vortex generator, online film emulsifying equipment, high-pressure homogenizer, microfluidic devices, fogging tester, and high-speed shearing dispersers.

Advanced engineering technologies are essential for the development of rapid, green, and simple preparation methods for controlling the physicochemical properties of emulsion systems. More specifically, through the use of the appropriate technology, extracts can be directly transformed into functional emulsions for practical applications. In this context, Fernandezavila et al. [230] explored the effects of ultrasonication on the properties and stability of an oil-in-water emulsion of *Pulicaria jaubertii* extract. They demonstrated a green shortcut to the transformation of plant extracts into functional nanoemulsions while also achieving enhancement of the active substance stability and antioxidant activity, thereby increasing the potential of such extracts for application in the food industry. 

The use of new technologies to protect bioactive substances while enhancing their release rates remains an ongoing challenge for the food industry [231]. Thus, to produce emulsions with stable physicochemical and microbiological parameters, ultra-high-pressure homogenization (UHPH) can be used. For example, Aguilar et al. [232] found that emulsions prepared by UHPH exhibited smaller particle sizes and more uniform microstructures than those prepared by conventional homogenization and high-temperature, short-term heating (i.e., 20 s at 70 °C). They also reported that a sterile emulsion prepared by UHPH was stable to oxidation for three months. Overall, their study facilitates the use of UHPH in the preparation of oil-in-water emulsions for conjugated linoleic acid delivery in functional foods. Importantly, the cavitation caused by high-intensity ultrasound irradiation can reduce the microbial contents of raw meat emulsions without altering their flavor, color, or nutritional quality.

As another technological example, thermosonography has been used for the homogenization of camel milk cream to reduce the sizes of fat particles and enhance emulsion stability, hardness, cohesion, and dynamic viscosity [233]. In addition, fermentation by lactic acid bacteria is a safe and green technology that is used to increase the emulsifying performance of egg yolk [234]. Similarly, full consideration should be given to the stretching of the droplet or bubble interface during production or transportation, as this induces continuous movement of the emulsion or foam. Currently, the effects of these deformations on the stabilities of emulsion-based foods are not entirely clear, although it is known that the rheological parameters of the interface play a key role in the stabilization and sensory modification of food emulsions [235]. In addition, Zhao et al. [236] found that changes in the interfacial properties of cream balls after homogenization and heat treatment are the key factors that influence their digestion in vitro. More specifically, compared with raw cream balls, those subjected to homogenization and heat treatment featured an increased total release of free fatty acids and a different fatty acid release profile. This behavior was attributed to the fact that homogenization reduced the size of the milk fat balls and attracted increased numbers of milk proteins to the cream ball surfaces to accelerate fat digestion. The resulting reduced number of glycosylated molecules in the cream balls and the lowered integrity of the phospholipid layer therefore resulted in an enhanced digestibility. It has also been reported that, compared with the phacoemulsification approach, membrane-assisted nanoemulsification could provide nanoemulsions with superior size and dispersion control, in addition to a lower energy consumption [237].

In contrast, emulsion-based foods may encounter stability problems, such as flocculation, aggregation, or demulsification during acidic and thermal cycling. Therefore, further research is required to establish the optimal relationships between the composition, process, engineering technique, and function to optimize the operational parameters of industrial processes. The key to this research lies in varying the structures, functions, safety profiles, and quality characteristics of emulsion-based food systems in different environments, under different physicochemical processing parameters, and using different preparation conditions. More specifically, techniques such as ultrasound, microwave irradiation, and high-pressure processing can alter the physicochemical and functional properties of food components, exerting a range of effects depending on processing parameters, conditions, and food substrates employed. Such modifications can impart the emulsions or emulsion-based foods with desirable properties; however, undesirable traits can also arise. Thus, by monitoring and adjusting the processing conditions, it becomes possible to obtain the desired interactions between the food components without destroying the main structure of the active compound.

#### 4.2.3. Safety, Nutrition, and Metabolism Profiles

To successfully expand the application scope of food emulsion systems, it is necessary to consider their textures and (multi)functional design, in addition to their sensory characteristics, their effective bioavailability and health effects, their nutritional properties and metabolic processes, quality control issues, and consumer acceptance and trust. Moreover, to advance research in this area and promote the application of such systems, safety evaluations are required, and the related regulatory approval processes must be considered. Thus, the following paragraphs describe the research trends of emulsion-based food systems from the viewpoints of their sensory properties, nutritional values, and safety.

It is well-known that the dynamic balance of flavor release and retention in food emulsions greatly affects the sensory quality and consumer acceptance of foods. However, this area has received very little attention in the context of emulsion systems. Thus, theoretical and mathematical models for predicting and controlling flavor release in real food systems, as well as for evaluating protein–flavor interactions, are required to understand the relationships between food emulsion systems and flavor substances. Moreover, studies focusing on the addition of various flavor substances, changes in the release/retention modes of these flavor compounds, and their ability to bind other substances can provide theoretical and experimental data to support the production of foods with desirable formulations and flavors. Evaluations of the effects of different processing methods and varying environmental conditions on the flavor binding/release behavior, sensory properties, and flavor release mechanisms both in vivo and in vitro would also be desirable. 

The key interactions between food emulsion systems and the human organism are the surface–interface interactions, wherein the surface films of many biochemical systems and processes are composed of surface-adsorbed biopolymer molecules. For example, the pectin–lysozyme complex is a novel bifunctional structure that enhances oral lubrication and the tastes of foods due to its favorable properties [238]. Following the intake of food, the tactile perception of oral friction is the first factor noted by the consumer, and the degree of oral lubrication directly affects the pleasure of food intake and the consumer acceptability. Thus, oral tribology has been used to influence the perceptions of foods, as exemplified by the smoothness and astringency of emulsions, creams, and gels [239]. In addition, Upadhyay et al. [240] discussed the oral tactile origin of the smoothness of emulsion-based foods, demonstrating that the oil content and the emulsifier type significantly affect the tribological behavior of such emulsifying systems. Moreover, they emphasized the important role of saliva in this process. Furthermore, Yang et al. [241] studied the structure and tribology of a natural oil–body and κ-carrageenan emulsion gel and established a relationship between the gel properties and its lubrication performance. Future research should therefore aim to improve the sensory qualities of emulsion-based food systems, such as tuning the rheological and tribological properties to simulate the oral processing of emulsions.

To date, the majority of studies in this area have focused on the development of delivery systems to improve the absorption, metabolism, and health effects of bioactive substances. The gastrointestinal digestion and absorption of emulsion-based foods directly affect the bioavailability of the active substances [242]. In contrast, few studies have considered the overall intake, transportation, digestion, and absorption processes, as well as the interactions between emulsion systems and the human body. An understanding of the complex interactions between emulsion-based foods and human saliva, the tongue [243], the oral cavity [244], the oral biofilm [245], the gastric pathway, the intestines, the human microbiome, and the catalase enzyme [246] is also desirable. More specifically, such an understanding should allow the evolution of the bioavailability and the activity of functional active components loaded by the dispersion system to be carried out, and it would also be expected to aid in the efficient screening of the biomaterial properties to optimize responses in vivo. Thus, the future design of food emulsion systems as innovative food ingredients will require further attention to the real bioavailability, the route from the oral cavity to the internal organs, and the establishment of the corresponding models, including those predicting the gastrointestinal fate. Moreover, the main factors affecting gastrointestinal digestion and release should also be considered, as should the associative interactions between hydrocolloids, the effective methods of improving the bioaccessibility and utilization efficiency, the specific absorption pathways, and the effects of colloidal substances on human intestinal probiotics and pathogenic bacteria [247]. For all kinds of macronutrients and bioactive substances, various in vitro and in vivo methods can be developed to explore the digestion and absorption processes based on gastrointestinal hydrolysis and lipolysis while also permitting the quantitative monitoring of structural changes and interactions [248]. Moreover, differences in the microstructures, physicochemical properties, and lipid/protein digestion processes in different regions of the gastrointestinal tract have also been investigated [249,250]. However, few studies have examined the digestive behavior and metabolism in animal experiments, and so, further research is needed to expand our knowledge in this field. Additionally, to clarify the biological effects of the corresponding substances and accelerate discoveries in personalized food and drug system designs, machine learning and intelligent algorithms can also be used [251]. 

In terms of the safety profiles, it must be considered whether the processing and utilization of food emulsion systems (including those of the basic raw materials, byproducts, and residues) are sufficiently safe. The nanostructures or other inactive components present in emulsion-based foods should also be evaluated due to their potential risk. The in vivo safety profiles of micro-/nanocolloidal particles are controversial, thereby highlighting the importance of researching the in vivo metabolic behavior (i.e., dynamic formation and decomposition processes), biological distribution, and safety of food colloids. Therefore, strict and clear guidelines should be issued for the use and evaluation of nanostructures in food technology, and these should be based on an evaluation of their toxicological data and nutritional characteristics. In this context, studies probing the fate of granular colloidal emulsions using simulated gastrointestinal digestion and intestinal mucosa are of great significance for safety and function evaluations [252]. In addition, a selection of studies has examined the antibacterial activities of emulsion systems in the environment while also discussing the origin of this activity [253]. However, to achieve the commercialization of interface-dominated functional foods, additional work is required to fully understand the antibacterial activities of nanoemulsions and to assess whether further safety control is needed for their practical application.

It should also be pointed out that the co-intake of food emulsion systems and foods poses certain hazards, such as those associated with pesticide residues, acrylamide, advanced glycation end-products, and toxic Maillard reaction products [254]. In this context, Zhang et al. [255] found that the simultaneous intake of emulsions with fruits or vegetables may increase the bioaccessibility of pesticides, such as hydrophobic pesticide residues that are present in natural products; however, this is dependent on the composition and structural properties of the ingested emulsion. In addition, Ke et al. [256] studied the interactions between acrylamide and micelles in the water extract of French fries, showing that such binding may affect the biological impact and toxicity of acrylamide by shielding its direct contact with cells or other biological substances. Ultimately, their study provided a new perspective for the risk assessment of acrylamide in food matrices. Future studies should explore the ability of food emulsion systems in the context of promoting the absorption of harmful substances. Moreover, it would be of interest to inhibit the formation of such substances to reduce their potentially harmful effects. The related structure–activity relationships, mechanisms of action, absorption and metabolic processes, and health risks are also of interest for future studies to promote the use of food emulsion systems as a new class of regulators and/or functional components. 

### 4.3. Material Applications

Emulsion systems can be widely used to promote the emergence of safe, healthy, convenient, and high-quality novel materials, including food particles and nanoparticles, microcapsules, biodegradable composites, porous materials, active films, edible coatings, hydrogels, and aerogels [257]. It should be pointed out that the applications of materials with different characteristics can vary, as can their related preparation methods. In research and production, appropriate preparation methods should be selected according to the material type and application. Thus, Table 2 presents details regarding the application of emulsion systems in material preparation, and a number of diversified research ideas are described below.

#### 4.3.1. Materials for Active Sustained-Release Membranes

Emulsified blend films based on konjac glucomannan/carrageenan/camellia oil have been reported to exhibit a good thermal stability, in addition to favorable optical and mechanical properties [298]. Consequently, film materials can be prepared using emulsion technology, wherein the oil type and concentration can affect the final film performance [299]. More specifically, carrageenan/agar-based functional films integrated with zinc sulfide nanoparticles and the Pickering emulsion of tea tree essential oil can be used for active packaging applications [300]. As reported by Wu et al. [301], packaging films based on Pickering emulsion commonly present functional antimicrobial and antioxidant activities. In addition, demulsification-induced rapid curing is a new method for the rapid preparation of polymer membrane materials with controlled structures, compositions, and functions, which can form stable mechanical connections between the polymer coating and the substrate [302]. In addition, this precoating accelerates water volatilization and helps to achieve rapid and effective adhesion between substrates, thereby rendering it applicable in the production of flexible devices and wearable materials.

Currently, emulsion-based active sustained-release membrane materials are an emerging research topic due to their ability to interact with foods and exert antioxidant and/or antibacterial effects and/or to prolong the shelf lives of foods through the controlled release of active agents during storage [303]. In addition, such systems can endow foods with specific nutritional enhancement functions. Food-grade nanoemulsions loaded with functional components are well suited for this role. Thus, using a gelatin- and chitosan-based coating matrix, Huang et al. [304] prepared a nanoemulsion-based edible coating containing rosemary extract (antioxidant) and ε-poly-l-lysine (antifungal). This coating effectively promoted the release of active compounds to the meat surface and improved the safety and quality of ready-to-eat carbonado chicken. In addition, Liu et al. [305] applied a composite nanoemulsion active film containing star anise essential oil, polylysine, and nisin to improve the quality and shelf lives of ready-to-eat meat products. Furthermore, the use of a chitosan nanoemulsion coating containing pomegranate peel extract (i.e., based on phenols and flavonoids as the active compounds) was demonstrated to effectively prolong the shelf lives of almonds [306].

The use of microemulsions to encapsulate antibacterial essential oils and the manufacture of active biopolymer packaging materials for foods using self-microemulsified essential oils have also been reported. More specifically, an antibacterial film based on food-grade self-microemulsified thyme essential oil was prepared using sodium alginate as the film-forming biopolymer, and it was revealed that the antibacterial effect significantly exceeded that of the corresponding nanoemulsion film [306]. More specifically, the microemulsion film completely released thyme oil in the form of oil-swollen micelles, while the nanoemulsion film released only 3% of the thyme oil upon contact with water. The former film exhibited an excellent antibacterial activity in ground beef, although it has been found that dual emulsion films loaded with dual antibacterial agents can induce more lasting antibacterial activities. For example, a composite edible film fabricated by incorporating a water-in-oil-in-water emulsion into a chitosan film was used to protect fresh fish meat [307]. However, to enhance the effect of the active membrane, the matrix that forms the membrane should be considered, as should the carrier of the active agent and any factors affecting the controlled release characteristics of the system. More specifically, it is known that the microstructure, molecular interactions, and environmental factors can affect the controlled release behavior of an active membrane, and so, the optimization of these factors could lead to novel applications for bio-based active membrane packaging in the food industry.

Future research in this area should ideally focus on the fabrication of intelligent emulsion-based active packaging films (i.e., those providing real-time signals responding to packaging conditions and changes in the food quality) [308], since these films can enable rapid, reliable, and online evaluation, which, together with visual food quality evaluations, can be applied to new active food packaging technologies. It has also been reported that dispersion systems loaded with natural food colorants can be integrated into pH- or gas/temperature-sensitive smart packaging coatings for monitoring the food quality [309]. For example, pH-responsive bilayer indicator films based on konjac glucomannan/camellia oil (the outer layer), and carrageenan/anthocyanin/curcumin (the indicator layer) can be used to monitor meat freshness [310]. In addition, the preparation of packaging materials capable of the controlled release of active compounds could allow further studies into controlled release kinetics and mechanisms, such as when and how to trigger the release, the extent of the release, and the release speed suitable for improving the food quality and safety during extended storage. The formulations of emulsion-based edible packaging materials should therefore be optimized for the development of edible packaging and coating materials that can control the release of bioactive substances [311]. Although the eugenol emulsion/chitosan edible coating provides a green coating for extending the shelf lives of fresh meat products [312]; consumers’ acceptance of such systems must also be considered [313].

#### 4.3.2. Particulate Materials

Microparticulate materials can be prepared using emulsion gels, which can be divided into bulk gels and gel particles according to their appearance, while gel particles can be categorized, according to their size, into gel beads (>1 mm), gel particles (0.2 µm–1 mm), and nanogel particles (<0.2 µm). Lin et al. [314] used an oil-in-water-in-oil multiple emulsion as a model to prepare a sodium alginate oil-in-water emulsion microgel and examined its microscopic morphology, rheology, and digestion properties. They also probed the effect of this model on the double gel system of an emulsion microgel prepared by an internal/external method to provide a reference for the applications of sodium alginate emulsion microgels in foods.

In view of their favorable structural properties, core–shell particles can encapsulate and protect functional compounds and regulate their transport and release. The interfacial polymerization of surfactants can be used to form core–shell particles based on emulsion or dispersion polymerization, as well as antisolvent precipitation. Food-grade alginate–gliadin hydrophilic–hydrophobic core–shell particles can be easily prepared by an antisolvent method based on gel network limitation [315]. In addition, seed hydrogel particles can be prepared using a water-in-oil emulsion as a template; subsequently, the dispersed phase of the emulsion is solidified, and hydrogel particles are obtained by means of phase inversion or filtration. The prepared core–shell particles were found to exhibit an excellent biocompatibility and biodegradability while also limiting swelling and improving the water resistance and continuous release properties, thereby providing novel ideas for the protection of unstable or hygroscopic compounds and bioactive substances while also contributing to flavor substance transport and controlled release. 

Emulsion polymerization can be used to synthesize hydrophilic crosslinked polymer microspheres that are well-suited for the in situ embedding of immobilized enzymes. This strategy allows the poor operational stability to be overcome while also reducing the diffusion distance and accelerating substrate and product diffusion. As an example, emulsion-immobilized enzyme technologies can play an important role in promoting the perception of food flavors [316]. More specifically, α-whey protein nanotubes can be formed by the self-assembly of partially hydrolyzed α-whey proteins and peptides to fix lipase. Such lipase nanotube-stabilized oil-in-water Pickering emulsions featured increased their enzymatic activities and promoted the release of fatty acids to improve the flavors of low-fat cheeses. The above study therefore inspired the development of immobilized enzyme carriers to effectively improve the flavor defects of low-fat foods. In brief, the fusion of the Pickering emulsion and immobilized enzyme technologies significantly broadens the application range of enzymes, while the use of cleverly designed immobilized biocatalyst reactors can realize automated production and greatly reduce production costs.

#### 4.3.3. Microcapsule Materials

Microencapsulation allows dispersed solids, liquids, and gases to be wrapped with semi-permeable or closed shells, thereby protecting the core material from environmental factors, masking bad flavors, and improving the solubility/fluidity. Emulsification is a key step in the process of oil microencapsulation, and the emulsion properties can affect the retention rate, microencapsulation efficiency, solubility, and oxidation stability of the microcapsule core material. As a result, investigations are ongoing to discover a variety of new ingredients for application in the microcapsule field [263]. To improve the efficiency and chemical stability of microcapsules, a small particle size and a uniform distribution of the oil droplets are required during the emulsion. Noncompliance with these requirements may result in deep depressions on the surfaces of the microcapsule powders, a loose inner wall, an abundance of cavities, and a low rate of active ingredient retention in the microcapsules [317]. To address these issues, Chen et al. [318] developed a structured film for the microencapsulation of DHA oil, which was inspired by milk fat globule membranes. More specifically, DHA-rich oil emulsions were prepared using different ratios of sunflower phospholipids; proteins (whey protein concentrate, soy protein isolate, and SC); and maltodextrin, wherein the DHA microcapsules were subsequently obtained by spray-drying. This system is of interest, since the microcapsules can protect the bioactivity of the DHA oil emulsion during its transport to the intestinal tract, thereby resulting in a high lipid digestibility and DHA hydrolysis efficiency in the small intestine. As another example, upon the microencapsulation of pepper essential oil, the associative colloid of soy protein and pectin formed during the bilayer emulsion exhibited a higher oil retention, lower water adsorption and cohesion properties, thicker walls, a smoother appearance, and a superior quality to those formed in a single-layer emulsion [319]. The preparation of phase-change material microcapsules using Pickering emulsions as the templates is a relatively simple and environmentally friendly process and affords products exhibiting superior stabilities and sustainabilities than traditional emulsions. In addition, Chen et al. [320] used an in situ precipitation strategy to prepare zein core–shell microcapsules with an adjustable shell thickness for the delivery of seaweed oil, and they demonstrated that these microcapsules could minimize the impact of lipid oxidation and improve the bioavailabilities of the loaded lipophilic substances. Similarly, microcapsules based on amyloid fibers (such as β-lactoglobulin, SPI, and lysozyme) that exhibit excellent emulsification and film-forming properties have been used to encapsulate bioactive compounds such as fish oil and lutein [321]. Furthermore, using a food emulsion system to carry out the micro- and nanoencapsulation of plant essential oils, which ensured the high stability and effective controlled release of the essential oils under harsh conditions while also improving the nutritional composition and antioxidant/antibacterial activities of foods and expanding the related application scope. 

Although microencapsulation allows the loading of natural pigments as food colorings, microcapsules loaded with β-carotene are unable to meet color demands. Thus, Gao et al. [322] explored a novel strategy to improve the color intensity of β-carotene by increasing the amount of β-carotene adsorbed on the microcapsule surface. Compared with the β-carotene encapsulated inside the water-in-oil emulsion droplets, the β-carotene particles on the droplet surface exhibited an improved color intensity. The use of Pickering emulsions to construct high-level assembly microcapsules with bionic membrane structures is a hotspot in the research of biomimetic materials and self-assembly. Inspired by the structure of bacterial hair, Dou et al. [323] used crystalline rod-shaped nanomicelles produced by high-level interface self-assembly (i.e., crystallization-driven self-assembly) to construct actively growing biomimetic microcapsules and provide new approaches for the encapsulation and transport of functional molecules. This system was also applicable in microreaction catalytic converters and biomolecule signal exchange. Indeed, it is already known that microcapsules capable of disintegrating or changing their permeability in response to environmental stimuli can be constructed using environmentally sensitive crosslinking agents. Cellulose-based liquid crystal microspheres can be formed by self-assembly in water-in-oil emulsions. The topological transition between the radial and bipolar forms of these microspheres is expected to provide biomedical materials for the development of stimuli-responsive bio-optical devices [324]. In addition, the Pickering emulsion template method can control the surface wettability properties of polymer microspheres and superhydrophobic surfaces, while super-oleophilic surfaces can be formed on polymer microspheres by means of the Pickering emulsion polymerization method [325]. Such materials with unique wettability properties can lead to the excellent separation of emulsions. In the future, it would be desirable to use microfluidic or size-controlled emulsification technologies to improve the size uniformity of microcapsules.

#### 4.3.4. Porous Materials

In recent years, the fabrication of biocompatible and biodegradable porous materials has attracted growing attention in the field of biomedical engineering. In addition, following the stabilization of an emulsion system, porous materials can be formed by removing the internal dispersed phase, and this technology can be applied to the construction of scaffolds for tissue engineering and regenerative medicine. Indeed, this straightforward method has received significant research interest. For example, HIPEs have become a mature medium for the preparation of porous materials owing to their dense inner phase droplet structure [326]. The selection and design of raw materials, methods, adjustable mechanical properties, and stimuli responses must therefore be considered in combination with the specific applications of scaffold materials. 

To date, emulsion-templated porous materials have been used for a range of applications [327]. Jiao et al. [181] stabilized HIPEs using peanut protein microgel particles, revealing that these emulsions can be rendered suitable for various applications simply by changing the composition of the internal phase. When the internal phase corresponds to an edible oil, it can be used to replace partially hydrogenated vegetable oil. In contrast, when the internal phase is *n*-hexane, the emulsion can be used as a template to produce porous materials. As the corresponding raw material is natural, nontoxic, biocompatible, and biodegradable, the resulting porous material can be employed in biological tissue engineering. In addition, Zhou et al. [328] constructed an all-natural protein-based porous material relying on Pickering emulsion templating and the Hofmeister effect. The emulsion stabilized by gliadin–chitosan hybrid particles was used as a template, and gelatin and osmotic ions were added to improve the mechanical strength, which resulted in the formation of nontoxic and biodegradable porous materials with highly open and controllable pore structures, excellent liquid absorption properties (i.e., a body fluid and rabbit blood mimic), and the ability to resist stress and maintain their geometry. These materials therefore have potential for application in many areas of biomedicine and materials, such as cell culturing, catalysis, and wound or bone defect healing.

##### D Printing Materials

The 3D food printing technology is well-suited for the design of convenient and flexible customized foods and to simplify the food supply chain. In this context, HIPEs have potential in the area of 3D food printing because of their good biocompatibility and dual emulsion/gel characteristics. Since the inside-to-outside (interface and crosslinking enhancements) and outside-to-inside (physical and environmental regulations) methods can improve the crosslinking degree and strength of the gel network, emulsion gels with enhanced mechanical properties can meet the requirements of 3D printing in terms of their shape, texture, taste, and functionality. In addition, Du et al. [329] found that the crosslinking of transglutaminase improved the thermal and storage stability of gelatin HIPEs and allowed 3D-printed models with high thermal stability to be obtained. In addition, Wang et al. [330] found that chitosan oligosaccharide-modified gelatin can be used to prepare HIPEs with a range of desirable properties, including a high viscosity, a high elasticity, and an excellent rheological stability, thereby allowing their application in 3D printing.

Moreover, it has been reported that HIPEs stabilized by co-assembled rice proteins and carboxymethyl cellulose are excellent inks for 3D printing, wherein their injectability and printability can be controlled by varying the substitution degree of carboxymethyl cellulose [331]. In addition, fatigue-resistant cod muscle fiber has been shown to stabilize tough and elastic HIPEs, which exhibit customizable rheological and textural properties, and, therefore, feature an excellent 3D printing performance and can be directly used in cake-based cream casting and printing [332]. The porous network structures of such HIPEs can also be used as templates for aerogel synthesis, and as another potential application, the ability of aerogels to efficiently absorb splashed (soybean) oil renders them well-suited for fabric cleaning and oil spill remediation. The above porous network structures can also be used as scaffolds for cell adhesion and proliferation in tissue engineering. Thus, the robust, simple, reusable, and biofriendly HIPEs described in this study can be developed into materials for diverse applications. Notably, potato starch can assist the preparation of well-structured 3D-printable fat analogs based on inulin emulsion-filled gels that resemble real meat fat. More specifically, potato starch inhibits melting during heat treatment and allows the generation of marbling in artificial meat [333]. Furthermore, soybean protein-based emulsion gels can be used to construct 3D-printed fat-reducing artificial meats and porous artificial plant meats with a high resolution and shape fidelity, thereby highlighting their contribution to meeting personalized customer needs [334].

Thus, future work in this area should focus on the in-depth study of emulsion-based material characteristics [335]; the development of instrumental or mathematical models; and the improvement of the material liquidity, gelation performance, and intelligence (i.e., the relationship between internal structure and various stimuli). Moreover, design optimization and the reorganization of emerging technologies (e.g., microwave and ultrasonic) may be combined to realize efficient printing, accurate product molding, and intelligent product quality control, as well as to develop personalized, shape-stable, and nutritious 3D foods [336]. Related applications can be therefore expanded to include interactive 4D-printed foods to meet the health needs of consumers and enhance the commercial value of such products. For example, printed customized functional soft food can be used to facilitate chewing and swallowing in the elderly or to solve problems such as vitamin D deficiencies [337].

#### 4.3.5. Material Research Prospects

Research into food emulsion systems should address a number of questions in terms of the materials employed for such applications. For example, how does one continue the development of high-performance, green, and safe basic raw materials for food emulsion systems? What are the limitations and challenges related to the extraction, applicability, and applications of basic materials? How do the selection and design of basic materials and interactions between different materials render dispersion systems more functional and valuable? How does one improve the selection and design processes for each spatial scale of the emulsion system? What materials can be developed and prepared based on dispersion systems, and how can these materials be used? Do the prepared materials have natural, high-quality, convenient, safe, sustainable, and biodegradable solutions? How does one render the prepared materials better-suited for practical applications? How does one integrate new elements such as constantly developing new technologies into material preparation? How does one further develop controllable and adjustable intelligent multi-effect material products? How do the surface interfaces of the prepared materials interact with the application environment? Do the developed materials achieve the desired effects and functions in specific applications? What other improvements are possible, how can they be implemented, and how effective are they? When materials are applied to organisms, how do the corresponding interactions further induce physiological processes and help to realize the biological functions of materials?

### 4.4. Other Emerging Applications

The application scope of food emulsion systems has continued to increase in recent years. For example, in the field of biomedicine, Xia et al. [338] creatively used a Pickering emulsion as a vaccine adjuvant. The flexibility and lateral fluidity of the developed system resulted in a viscoelasticity, fluidity, and surface roughness similar to those of natural pathogens, which promoted the research of vaccine adjuvants to the stage of intelligent bionics. In addition, the above emulsion system exhibited efficient antigen loading, a good safety and stability, and an excellent immune activity while avoiding the use of surfactants and inspired the design of antitumor, antibacterial, and antiviral vaccine adjuvants. In this context, the Chinese yam polysaccharide poly (lactic-*co*-glycolic acid) (PLGA) nanoparticle-stabilized Pickering emulsion [339] was employed as a novel adjuvant, as were alhagi honey polysaccharides encapsulated into PLGA nanoparticle-based Pickering emulsions [340], and these adjuvants were found to induce strong, efficient, and long-lasting immune responses. In addition, Mosquera et al. [341] developed an immunomodulatory nanogel vaccine that increased the immune responses in male mice with a metabolic syndrome, thereby providing a new potential for application for nanoscale materials.

Pickering emulsions can also be developed into sustained-release nontoxic platforms for antimicrobial peptides. For example, Cai et al. [342] controlled the emergence of bacterial drug resistance using a stable Pickering emulsion with antimicrobial peptide nanoparticles (formed by the interaction of the catfish antimicrobial peptide with lecithin or chitosan), and they achieved milder and more effective anti-inflammatory effects in the case of peritonitis. Such systems may therefore provide a means to combat the issues related to multidrug-resistant bacteria in biomedical applications. In terms of antiviral therapeutics and nanodelivery systems, nanoemulsions are of particular interest. For example, Larsson et al. [343] reported that self-assembled surfactants form nanomicelles in solutions, and so, they may endow drugs with the required properties. Additionally, Dos Santos et al. [344] generated large monolayer membrane vesicles based on the droplet microfluidic technology of the double-emulsion method and simulated the intracellular signaling pathway by encapsulating different enzyme combinations within the droplets. This method fully simulated the biochemical reaction environment of cells, and so, it was considered to be suitable for modeling almost all multistep enzymatic reaction pathways while also facilitating the discovery of some side reactions and byproducts, as well as their mechanisms of formation.

In the field of agricultural pesticides, Ma et al. [345] used a 1 wt% solution of saponin glycyrrhizic acid (a natural triterpenoid with an excellent self-assembly behavior) to prepare an environmentally friendly agricultural Pickering emulsion gel with an agricultural oil content of 80 wt%. As a result, they significantly reduced the traditional surfactant dosage and endowed the system with an excellent acid–alkali resistance and thixotropic properties, thereby providing a novel method aimed at pesticide storage and transportation and at reducing agricultural environmental pollution. In addition, it has been reported that the combination of a larvicidal composite alginate hydrogel with a Pickering emulsion of an essential oil increased the corresponding larvicidal activity because of the higher release rate [346]. In terms of sample pretreatment, He et al. [347] used biochar as a Pickering emulsion stabilizer to prepare biochar composite microspheres with regular morphologies, controllable particle sizes, and high specificities; this was achieved by means of Pickering emulsion polymerization combined with molecular imprinting technology.

Finally, bioactive emulsions with beneficial antimicrobial properties have been demonstrated to be applicable in the production of textile materials [348]. More specifically, Pickering emulsions find applications in the preparation of phase change materials [349] and the generation of hybrid foams exhibiting high-performance microwave absorption properties [350]. In addition, they have been applied to enhance the stabilization and paper sizing performance of various materials [351], to prepare self-healing composite coatings [352], and to limit the influx of metal ions [353]. Additionally, they can be used in cosmetic applications [354], such as in the case of a nanoemulgel that employs *Argania spinosa* microfibrillated cellulose and natural emulsifiers [355]. In further works, the application of emulsions in cosmetics should focus on formulation to ensure the desired level of skin penetration and the corresponding risk assessments [356].

## 5. Conclusions

This work reviews the applications of food emulsions at different structural levels from a new perspective and discusses a range of influencing factors based on inherent structural advantages to inspire the development of future applications. Different types of emulsion systems are known to exhibit certain similarities and feature particularities; therefore, it is desirable to combine the essential features with the related application demands to pave the way for new applications and allow a fully integrated comparative analysis of different emulsion systems. Moreover, the application potential for the targeted design of emulsion dispersion systems should be evaluated, as should the development of new food ingredients or products with good nutritional, functional, sensory properties, and biological activities. To effectively highlight the respective advantages of different food emulsion systems, improvements in the design and performances are necessary by manipulating the emulsion structure via a combination of strategies. Such strategies may include the selection or preparation of components in the aqueous phase, at the interface, or in a dispersed-phase matrix. Therefore, it is necessary to build a coherent multiscale theoretical system for the evaluation of different emulsions (i.e., on the microscopic, mesoscopic, and macroscopic scales) to support and expand the experimental results discussed herein. For example, simulation methods can accurately predict and guide the structural and functional design of emulsions while also actively regulating the emulsion properties to meet the requirements of different applications. Such approaches are expected to optimize the commercial usage and consumption of emulsions, in addition to removing various barriers in the production and processing stages, such as safety issues, production costs, environmental consequences, sustainability, consumer acceptance and trust, government regulations, and labeling issues. 

Given that no comparative summary is currently available that addresses applied research based on the essential characteristics of emulsion systems in variable-scale hierarchies, we believe that our study will help researchers better understand the existing problems and further promote the efficient utilization of food-grade emulsions.

## Figures and Tables

**Table 1:** Applications of food-grade emulsions in soft matter delivery.

Emulsion Type	Emulsifying Stabilizer	Delivery Material	Advantages	Reference
O/W	whey protein hydrolysates after succinylation and glycation in different orders	curcumin	enhanced stability and bioavailability	[17]
O/W	whey protein isolate (WPI)	β-carotene	enhanced retention rate and bioavailability	[18]
O/W	WPI, sodium alginate	lycopene	enhanced stability and bioaccessibility	[19]
O/W	high methoxyl pectin-rhamnolipid-pea protein isolate-curcumin complex	β-carotene	enhanced stability and bioaccessibility	[20]
O/W	β-lactoglobulin-ferulic acid-chitosan ternary conjugate	β-carotene	enhanced physicochemical stability	[21]
O/W	a ternary co-orbital compound covalently assembled from bovine serum albumin, chlorogenic acid and dextran	lutein	enhanced stability, solubility and bioavailability	[22]
O/W bilayer-	lactoferrin beet pectin, or carboxymethyl chitosan,	astaxanthin	enhanced physicochemical properties and bioactivity	[23]
O/W bilayer-	WPI and chitosan	lycopene	provide a better protection on physical characteristics, storage and light stability	[24]
O/W nano-		peppermint oil	enhanced stability, antibacterial	[25]
O/W nano-	tea saponins	silymarin	tea saponins as emulsifiers and cryoprotectants to prevent the irreversible aggregation of droplets during freeze-drying	[26]
O/W nano-	rice bran protein	quercetin	enhanced stability and bioavailability	[27]
O/W nano-	tea polysaccharide conjugate	β-carotene	enhanced physicochemical stability and bioaccessibility	[28]
O/W nano-	soybean protein isolate (SPI)-phosphatidylcholine	fish oil	enhanced stability and digestibility under simulated gastrointestinal conditions	[29]
O/W nano-	SPI-dextran conjugation	capsicum annuum L.	enhanced physical stability with good encapsulation efficiency	[30]
O/W nano-	phospholipid	berberine	enhanced hypoglycemic efficacy	[31]
O/W nano-	zein-polyglycerol conjugate	resveratrol	enhanced chemical stability, in vitro gastrointestinal digestion, and antioxidant activity	[32]
O/W Pickering	chitin nanofibrils	cinnamon cassia oil	enhanced diffusion efficiency, controlled release of EOs, prolonged antibacterial activities	[33]
O/W Pickering	cellulose nanocrystals	clove oil	enhanced antibacterial activity	[34]
O/W Pickering	zein-adzuki bean seed coat polyphenol covalent crosslinking nanoparticles	polyphenol	inhibite lipid oxidation and promote in vitro digestion	[35]
O/W Pickering	waxy maize starch nanocrystal and chitosan	resveratrol	enhanced gut-retention time of resveratrol	[36]
O/W Pickering	glycated proteins and chitooligosaccharides	curcumin	enhanced stability	[37]
O/W Pickering	β-lactoglobulin-(−)-Epigallocatechin-3-gallate (EGCG) composite colloidal nanoparticles	lutein	encapsulate and control release of lipophilic components with high antioxidant performance	[38]
O/W Pickering	maillard-reacted WPI and epigallocatechin gallate complex	curcumin	enhanced thermal stability	[39]
O/W Pickering	chitosan-hydrophobic alginate nanocomposites	Ibuprofen	pH-triggered for drug controlled-release	[40]
O/W Pickering	zein-pectin composite nanoparticles	cinnamon essential oil	good dispersibility and continuous release ability, slow-release antimicrobial in storage application	[41]
O/W Pickering	se-enriched peanut protein nanoparticles	5-demethylnobiletin from chenpi	enhanced bioaccessibility and in vitro absorption	[42]
O/W Pickering	complexation of carboxymethyl cellulose/cationic chitosan	curcumin	enhanced encapsulation and stability	[43]
O/W Pickering	α-Lactalbumin self-assembled nanoparticles	curcumin	enhanced bioavailability	[44]
O/W Pickering emulsion gels	chitosan hydrochloride-carboxymethyl starch and curdlan	β-carotene	protect bioactive components and designing low-fat food alternatives	[45]
O/W HIPEs	chitosan and xanthan gum	β-carotene	efficient transportation	[46]
O/W HIPEs	biomass lignin	β-carotene	anti-photo and anti-thermal	[47]
O/W HIPEs	chitosan	β-carotene	high loading contents and stability	[48]
O/W HIPEs	WPI microgels	lactobacillus plantarum	enhanced the viability	[49]
O/W HIPEs	β-Lactoglobulin-propylene glycol alginate composite hydrogel particles	probiotics and curcumin	co-encapsulation enhanced survivability and controlled release	[50]
O/W HIPEs	gliadin nanoparticles/gum arabic	β-carotene	enhanced stability and bioavailability	[51]
O/W HIPEs	pecan protein/xanthan gum	quercetin	enhanced stability and bioaccessibility	[52]
O/W HIPEs	ovalbumin-pectin complexes	curcumin	enhanced stability and bioaccessibility	[53]
O/W HIPEs	pea protein isolate-high methoxyl pectin-EGCG complex	EGCG	high antioxidant performance	[54]
O/W HIPEs	WPI-EGCG covalent conjugate nanoparticles	lactobacillus plantarum	enhanced storage and gastrointestinal passage viability	[55]
O/W HIPEs	complexes of sugar beet pectin, tannic acid, and chitosan	curcumin	enhanced stability and bioaccessibility	[56]
O/W HIPEs	pea protein and high methoxyl pectin colloidal particles	β-carotene	enhanced chemical stability and controlled release property	[57]
W/O HIPEs	sodium alginate	hydrophobic capsaicin	controlled release, reduced irritation, and enhanced bioaccessibility	[58]
W/O HIPEs		bitter peptides	reduced bitterness, enhanced gastrointestinal stability	[59]
W/O HIPEs	polyglycerin ricinoleate	probiotic	enhanced viability under food and gastrointestinal conditions	[60]
emulsion gels	pectin, rhamnogalacturonan-I	curcumin	protection and sustained release of curcumin	[61]
emulsion gels	gellan gum	pancreatic lipase	trigger (cargo) release	[62]
S/O/W	caseinate, lecithin	lactase	protect activity during storage and digestion, and control in vitro hydrolysis of lactose in milk	[63]
W/O/W	polyglycerol polyricinoleate (PGPR)	iron	the addition of encapsulated iron had no negative effects on the sensory general acceptability	[64]
W/O/W	PGPR	anthocyanin	prevent anthocyanin degradation and maximise double emulsion stability and protect anthocyanin colour retention and prevent leakage	[1]
W/O/W	black soya bean protein, PGPR	Insulin, quercetin	bioaccessibility, chemical stability and solubility under simulated gastrointestinal conditions increase	[65]
W/O/W	PGPR	lactobacillus reuteri	protect the probiotics during storage and in vitro gastro-intestinal digestion	[66]
W/O/W	PGPR	resveratrol	enhanced bioavailability, physicochemical stability, in vitro digestion resistivity and transport properties	[67]
W/O/W	whey protein concentrate-high methoxyl pectin, PGPR	brassinolide and cinnamon essential oil	delayed the senescence of broccoli via regulating chlorophyll degradation and energy metabolism	[68]
W/O/W	PGPR, quillaja saponin, gum arabic	anthocyanin	anthocyanin was successfully embedded	[69]
W/O/W nano-	PGPR	unsaturated guluronate oligosaccharide	address the storage difficulties and provides in vitro sustained-release	[70]
W/O/W HIPEs	zein nanoparticles and soy lecithin		the water droplets inside are protected by strong gastric juices	[71]

**Table 2:** Applications of food-grade emulsions in material preparation.

Prepared Material	Emulsion Type	Emulsifying Stabilizer	Preparation Method	Advantages	Reference
microcapsule	O/W	zein and whey protein concentrate	emulsion-electrospraying technology	enhanced the bioaccessibility of β-carotene	[258]
microcapsules	O/W Pickering	cellulose nanocrystal	emulsion in-situ polymerization	promising for temperature regulation and thermal energy storage	[259]
microencapsulation	O/W Pickering	polystyrene/cellulose nanocrystal hybrid shell	emulsion polymerization	highly efficient, economical, green and has designable characteristics (adjusting phase change temperature and shell conditions)	[260]
microencapsulation	O/W	gelatin, sodium	complex coacervation	enhanced ω-3 carrying capacity, oxidation stability of fish oil and the formation of the microcapsules with smoother, denser structure, superior mechanical strength	[261]
microencapsulation	O/W nano-	modified starches	spray dried nanoemulsions	the microcapsules containing curcumin, resveratrol and borage seed oil had good physical and chemical properties and retention rate	[262]
microcapsules	O/W Pickering	chitosan-decorated silica nanoparticles	spray dried emulsion templating	peppermint oil-loaded and antibacterial improvement	[263]
capsules	O/W/O	cellulose nanocrystals	emulsion polymerization	as a promising packaging container effectively	[264]
microcapsules	O/W Pickering		emulsion polymerization	as multifunctional coatings	[265]
microspheres	O/W Pickering		emulsion template	hydroxyapatite-loaded magnetic polycaprolactone hollow microspheres for malachite green adsorption	[266]
green hydrophilic capsules	O/W Pickering	cellulose nanocrystal	emulsion polymerization	morphology control and spongelike behavior	[267]
gel beads	O/W	WPI/dextran Maillard conjugate	emulsification and ionotropic gelation	the activity of probiotics is high, but rapeseed canola oil is not suitable for long-term storage co-embedding probiotics	[268]
microstructured gel beads	O/W	alginate-caseinate matrix	emulsification-gelation technique	the iron encapsulated gel beads can develop as a promising safe iron fortifier by relieving lipid oxidation and iron odor	[269]
chitosan nanoparticles	W/O	span80	hydrodynamic cavitation to intensify the emulsion cross-linking process	the preparation of a finer and a narrower distribution of NPs in a more energy-efficient manner	[270]
nanospheres	inverse Pickering mini-	ZnO@PNIPAM	inverse Pickering miniemulsion polymerization	good dispersion	[271]
calcium-lipid microspheres	S/O/W	sodium caseate-gelatin		enhanced dispersion stability and digestive properties of CaCO_3_ in liquid foods	[272]
biodegradable composite material	O/W Pickering	regenerated cellulose	the combination method based on Pickering emulsion	the method to prepare cellulose reinforced biodegradable composites is simple, environmentally friendly and low cost	[273]
edible coating	O/W	persian gum	combination of full silicone rubber coated with clove and thyme essential oil emulsion	the texture, smell, color, the microbial quality and overall acceptability of coated fish fillets were significantly improvment	[274]
edible Films	O/W Pickering	dialdehyde cellulose nanocrystals		dihydromyricetin-loaded provide antioxidant	[275]
composite films	O/W Pickering	cellulose nanocrystal	emulsion casting technology	enhanced film water-resistance and antibacterial activity	[276]
edible coating	beeswax-in-water Pickering	cellulose nanofibrils/carboxymethyl chitosan	emulsion casting technology	promising applications in antiseptic and fresh keeping for berry fruits	[277]
coating	O/W Pickering	chitosan, cellulose nanofiber	emulsion casting technology	sandalwood oil can improve antifungal features and properties for fresh fruit application	[278]
coating	O/W Pickering	starch-beeswax	nanomaterial- emulsion coating	extend produce shelf-life	[279]
active film	O/W nano-	gelatin, chitosan, sodium caseinate	emulsion casting technology	have appropriate physical properties and strong antioxidant activity, and can as an active and biodegradable packaging material for food preservation	[280]
active film	O/W nano-	pullulan coating incorporated with cinnamon essential oil nanoemulsion	emulsion casting technology	Improvement of storage quality of strawberries	[281]
antimicrobial bilayer films	O/W Pickering	zein, chitosan	polylactic acid/Pickering emulsion	enhanced the mechanical properties (with tight, uniform, binds firmly), ductility and moisture resistance, and enhanced the oxygen barrier, continuously releases thymol and has directional release effect	[282]
nanohydrogels	W/O	starch	reverse emulsification and internal gelation	with great potential in many application fields, such as food, nutrition and health products and pharmaceuticals	[283]
food 3D printing	O/W HIPEs	sea bass protein microgel particles		with the extrudability, printing performance, and self-supporting properties	[284]
solid foams	O/W Pickering	microcrystalline cellulose/chitosan complex particles	emulsion templating	with porous structure, high strength, energy absorption, safe, clean and green	[199]
solid foams	O/W HIPEs	chitin nanofibrils	HIPEs templating	with 3D structuring	[285]
nanoparticles	O/W	Poly (lactic-co-glycolic acid)	emulsion-solvent evaporation	Improve embedding rate and physical stability, solubility, prolong circulation in blood and maintain release, which is beneficial to the maintenance of drug effectiveness and long-term treatment	[286]
composite nanoparticles	O/W	β-carotene-zein-propylene glycol alginate	emulsification-evaporation method	Improve physicochemical stability and in vitro simulated gastrointestinal digestion	[287]
fine particles	O/W	ethyl cellulose	ternary system-based emulsion castings	porous and spherical ethyl cellulose fine particles	[288]
microparticles	O/W/O, W/O/W/O	cellulose	multiple emulsion templating	microparticles have hierarchical internal structures	[289]
microporous particles	O/W Pickering		emulsion polymerization	as thermal insulation coatings, can save energy consumption in many areas	[290]
porous bioceramics	O/W Pickering		emulsion templating	application for bone regeneration	[291]
porous 3D scaffolds	O/W Pickering	cellulose and chitosan nanofibers	emulsion templating	bioadaptive porous 3D scaffolds	[292]
porous hollow fibers	O/W HIPEs		HIPEs templating	controllable structures	[293]
porous hydrogels	O/W HIPEs	soy-protein	HIPEs templating	highly interconnected structures with large voids show enhanced absorption behavior	[294]
hierarchically porous monolith	W/O HIPEs		HIPEs templating	high MOF accessibility and strengthened mechanical properties	[295]
porous materials	O/W HIPEs	gliadin–chitosan complex particles	HIPEs templating	water-insoluble, nontoxic and high porosity	[296]
phase change material	O/W HIPEs		HIPEs polymerized	shape stabilization and as thermal energy storage	[297]

## Data Availability

Data is contained within the article.

## References

[1] Sebben D.A., MacWilliams S.V., Yu L., Spicer P.T., Bulone V., Krasowska M., Beattie D.A. (2021). Influence of Aqueous Phase Composition on Double Emulsion Stability and Colour Retention of Encapsulated Anthocyanins. Foods.

[2] Wan J., Ningtyas D.W., Bhandari B., Liu C., Prakash S. (2022). Oral perception of the textural and flavor characteristics of soy-cow blended emulsions. J. Texture Stud..

[3] Lin F., Zhao X., Yang S., He F., Qin W., Gong H., Yu G., Feng Y., Li J. (2021). Interfacial regulation and visualization of Pickering emulsion stabilized by Ca^2+^-triggered amphiphilic alginate-based fluorescent aggregates. Food Hydrocoll..

[4] Du X., Hu M., Liu G., Qi B., Zhou S., Lu K., Xie F., Zhu X., Li Y. (2022). Development and evaluation of delivery systems for quercetin: A comparative study between coarse emulsion, nano-emulsion, high internal phase emulsion, and emulsion gel. J. Food Eng..

[5] Liu C., Wang R., He S., Cheng C., Ma Y. (2020). The stability and gastro-intestinal digestion of curcumin emulsion stabilized with soybean oil bodies. LWT.

[6] Wang D., Ma Y., Wang Q., Huang J., Sun R., Xia Q. (2019). Solid Self-Emulsifying Delivery System (S-SEDS) of Dihydromyricetin: A New Way for Preparing Functional Food. J. Food Sci..

[7] Alehosseini E., Jafari S.M., Tabarestani H.S. (2021). Production of D-limonene-loaded Pickering emulsions stabilized by chitosan nanoparticles. Food Chem..

[8] Light K., Karboune S. (2021). Emulsion, hydrogel and emulgel systems and novel applications in cannabinoid delivery: A review. Crit. Rev. Food Sci. Nutr..

[9] Chen M., Wang W., Xiao J. (2022). Regulation Effects of Beeswax in the Intermediate Oil Phase on the Stability, Oral Sensation and Flavor Release Properties of Pickering Double Emulsions. Foods.

[10] Chen X.-H., Tang C.-H. (2021). Highly transparent antioxidant high internal phase emulsion gels stabilized solely by C-phycocyanin: Facilitated formation through subunit dissociation and refractive index matching. Colloids Surf. A Physicochem. Eng. Asp..

[11] Xu Y.-T., Tang C.-H., Liu T.-X., Liu R. (2018). Ovalbumin as an outstanding Pickering nanostabilizer for high internal phase emulsions. J. Agric. Food Chem..

[12] Shi Y., Ye F., Zhu Y., Miao M. (2022). Development of dendrimer-like glucan-stabilized Pickering emulsions incorporated with β-carotene. Food Chem..

[13] Ju M., Zhu G., Huang G., Shen X., Zhang Y., Jiang L., Sui X. (2020). A novel pickering emulsion produced using soy protein-anthocyanin complex nanoparticles. Food Hydrocoll..

[14] Dai T., Li T., Li R., Zhou H., Liu C., Chen J., McClements D.J. (2020). Utilization of plant-based protein-polyphenol complexes to form and stabilize emulsions: Pea proteins and grape seed proanthocyanidins. Food Chem..

[15] Fathi M., Vinceković M., Jurić S., Viskić M., Režek Jambrak A., Donsì F. (2019). Food-Grade Colloidal Systems for the Delivery of Essential Oils. Food Rev. Int..

[16] Tan C., Dadmohammadi Y., Lee M.C., Abbaspourrad A. (2021). Combination of copigmentation and encapsulation strategies for the synergistic stabilization of anthocyanins. Compr. Rev. Food Sci. Food Saf..

[17] Pan Y., Li X.-M., Meng R., Zhang B. (2019). Exploration of the stabilization mechanism and curcumin bioaccessibility of emulsions stabilized by whey protein hydrolysates after succinylation and glycation in different orders. J. Agric. Food Chem..

[18] Zhao R., Fu W., Chen Y., Li B., Liu S., Li Y. (2022). Structural modification of whey protein isolate by cinnamaldehyde and stabilization effect on β-carotene-loaded emulsions and emulsion gels. Food Chem..

[19] Liang X., Yan J., Guo S., McClements D.J., Ma C., Liu X., Liu F. (2021). Enhancing lycopene stability and bioaccessibility in homogenized tomato pulp using emulsion design principles. Innov. Food Sci. Emerg. Technol..

[20] Guo Q., Bayram I., Shu X., Su J., Liao W., Wang Y., Gao Y. (2022). Improvement of stability and bioaccessibility of β-carotene by curcumin in pea protein isolate-based complexes-stabilized emulsions: Effect of protein complexation by pectin and small molecular surfactants. Food Chem..

[21] Wang D., Lv P., Zhang L., Yang S., Wei Y., Mao L., Yuan F., Gao Y. (2020). Enhanced physicochemical stability of β-carotene emulsions stabilized by β-lactoglobulin− ferulic acid− chitosan ternary conjugate. J. Agric. Food Chem..

[22] Yan Y., Zhu Q., Diao C., Wang J., Wu Z., Wang H. (2020). Enhanced physicochemical stability of lutein-enriched emulsions by polyphenol-protein-polysaccharide conjugates and fat-soluble antioxidant. Food Hydrocoll..

[23] Zhao T., Ma D., Mulati A., Zhao B., Liu F., Liu X. (2021). Development of astaxanthin-loaded layer-by-layer emulsions: Physicochemical properties and improvement of LPS-induced neuroinflammation in mice. Food Funct..

[24] Lv P., Wang D., Liang R., Liu J., Li J., Gao Y., Zhang J., Yuan F. (2021). Lycopene-loaded bilayer emulsions stabilized by whey protein isolate and chitosan. LWT.

[25] Liu Q., Gao Y., Fu X., Chen W., Yang J., Chen Z., Wang Z., Zhuansun X., Feng J., Chen Y. (2021). Preparation of peppermint oil nanoemulsions: Investigation of stability, antibacterial mechanism and apoptosis effects. Colloids Surf. B Biointerfaces.

[26] Deng M., Chen H., Xie L., Liu K., Zhang X., Li X. (2022). Tea saponins as natural emulsifiers and cryoprotectants to prepare silymarin nanoemulsion. LWT.

[27] Chen W., Ju X., Aluko R.E., Zou Y., Wang Z., Liu M., He R. (2020). Rice bran protein-based nanoemulsion carrier for improving stability and bioavailability of quercetin. Food Hydrocoll..

[28] Li Q., Shi J., Du X., McClements D.J., Chen X., Duan M., Liu L., Li J., Shao Y., Cheng Y. (2021). Polysaccharide conjugates from Chin brick tea (*Camellia sinensis*) improve the physicochemical stability and bioaccessibility of β-carotene in oil-in-water nanoemulsions. Food Chem..

[29] Li Y., Li M., Qi Y., Zheng L., Wu C., Wang Z., Teng F. (2020). Preparation and digestibility of fish oil nanoemulsions stabilized by soybean protein isolate-phosphatidylcholine. Food Hydrocoll..

[30] Zhou Y., Teng F., Tian T., Sami R., Wu C., Zhu Y., Zheng L., Jiang L., Wang Z., Li Y. (2020). The impact of soy protein isolate-dextran conjugation on capsicum oleoresin (*Capsicum annuum* L.) nanoemulsions. Food Hydrocoll..

[31] Xu H.-Y., Liu C.-S., Huang C.-L., Chen L., Zheng Y.-R., Huang S.-H., Long X.-Y. (2019). Nanoemulsion improves hypoglycemic efficacy of berberine by overcoming its gastrointestinal challenge. Colloids Surf. B Biointerfaces.

[32] Zhu P., He J., Huang S., Han L., Chang C., Zhang W. (2021). Encapsulation of resveratrol in zein-polyglycerol conjugate stabilized O/W nanoemulsions: Chemical stability, in vitro gastrointestinal digestion, and antioxidant activity. LWT.

[33] Huang Y., Liu H., Liu S., Li S. (2020). Cinnamon Cassia Oil Emulsions Stabilized by Chitin Nanofibrils: Physicochemical Properties and Antibacterial Activities. J. Agric. Food Chem..

[34] Yu H., Huang G., Ma Y., Liu Y., Huang X., Zheng Q., Yue P., Yang M. (2021). Cellulose nanocrystals based clove oil Pickering emulsion for enhanced antibacterial activity. Int. J. Biol. Macromol..

[35] Ge S., Jia R., Liu W., Xie J., Liu M., Cai D., Zheng M., Liu H., Liu J. (2022). Lipid oxidation and in vitro digestion of pickering emulsion based on zein-adzuki bean seed coat polyphenol covalent crosslinking nanoparticles. Food Chem..

[36] Jo M., Ban C., Goh K.K., Choi Y.J. (2021). Enhancement of the gut-retention time of resveratrol using waxy maize starch nanocrystal-stabilized and chitosan-coated Pickering emulsions. Food Hydrocoll..

[37] Yu J., Wang Q., Zhang H., Qin X., Chen H., Corke H., Hu Z., Liu G. (2021). Increased stability of curcumin-loaded pickering emulsions based on glycated proteins and chitooligosaccharides for functional food application. LWT.

[38] Su J., Guo Q., Chen Y., Mao L., Gao Y., Yuan F. (2020). Utilization of β-lactoglobulin- (−)-Epigallocatechin- 3-gallate(EGCG) composite colloidal nanoparticles as stabilizers for lutein pickering emulsion. Food Hydrocoll..

[39] Liu G., Wang Q., Hu Z., Cai J., Qin X. (2019). Maillard-reacted whey protein isolates and epigallocatechin gallate complex enhance the thermal stability of the pickering emulsion delivery of curcumin. J. Agric. Food Chem..

[40] Mao Q., Li M., Zhang S., Zhang X., He G., Zhang W. (2020). Chitosan-hydrophobic alginate nanocomposites stabilized pH-triggered Pickering emulsion for drug controlled-release. Int. J. Biol. Macromol..

[41] Jiang Y., Wang D., Li F., Li D., Huang Q. (2020). Cinnamon essential oil Pickering emulsion stabilized by zein-pectin composite nanoparticles: Characterization, antimicrobial effect and advantages in storage application. Int. J. Biol. Macromol..

[42] Ning F., Wang X., Zheng H., Zhang K., Bai C., Peng H., Huang Q., Xiong H. (2019). Improving the bioaccessibility and in vitro absorption of 5-demethylnobiletin from chenpi by se-enriched peanut protein nanoparticles-stabilized pickering emulsion. J. Funct. Foods.

[43] Zhu X., Chen J., Hu Y., Zhang N., Fu Y., Chen X. (2021). Tuning complexation of carboxymethyl cellulose/cationic chitosan to stabilize Pickering emulsion for curcumin encapsulation. Food Hydrocoll..

[44] Liu B., Liu B., Wang R., Li Y. (2021). α-Lactalbumin self-assembled nanoparticles with various morphologies, stiffnesses, and sizes as pickering stabilizers for oil-in-water emulsions and delivery of curcumin. J. Agric. Food Chem..

[45] Li X.-M., Meng R., Xu B.-C., Zhang B. (2021). Investigation of the fabrication, characterization, protective effect and digestive mechanism of a novel Pickering emulsion gels. Food Hydrocoll..

[46] Huang M., Wang J., Tan C. (2021). Tunable high internal phase emulsions stabilized by cross-linking/electrostatic deposition of polysaccharides for delivery of hydrophobic bioactives. Food Hydrocoll..

[47] Chen K., Lei L., Qian Y., Yang D., Qiu X. (2019). Development of anti-photo and anti-thermal high internal phase emulsions stabilized by biomass lignin as a nutraceutical delivery system. Food Funct..

[48] Li W., Nian Y., Huang Y., Zeng X., Chen Q., Hu B. (2019). High loading contents, distribution and stability of β-carotene encapsulated in high internal phase emulsions. Food Hydrocoll..

[49] Su J., Wang X., Li W., Chen L., Zeng X., Huang Q., Hu B. (2018). Enhancing the viability of Lactobacillus plantarum as probiotics through encapsulation with high internal phase emulsions stabilized with whey protein isolate microgels. J. Agric. Food Chem..

[50] Su J., Cai Y., Tai K., Guo Q., Zhu S., Mao L., Gao Y., Yuan F., Van der Meeren P. (2021). High-internal-phase emulsions (HIPEs) for co-encapsulation of probiotics and curcumin: Enhanced survivability and controlled release. Food Funct..

[51] Ma L., Zou L., McClements D.J., Liu W. (2020). One-step preparation of high internal phase emulsions using natural edible Pickering stabilizers: Gliadin nanoparticles/gum Arabic. Food Hydrocoll..

[52] Huang M., Wang Y., Ahmad M., Ying R., Wang Y., Tan C. (2021). Fabrication of pickering high internal phase emulsions stabilized by pecan protein/xanthan gum for enhanced stability and bioaccessibility of quercetin. Food Chem..

[53] Wang L., Zhang H., Li H., Zhang H., Chi Y., Xia N., Li Z., Jiang L., Zhang X., Rayan A.M. (2022). Fabrication and digestive characteristics of high internal phase Pickering emulsions stabilized by ovalbumin-pectin complexes for improving the stability and bioaccessibility of curcumin. Food Chem..

[54] Feng T., Wang X., Wang X., Zhang X., Gu Y., Xia S., Huang Q. (2021). High internal phase pickering emulsions stabilized by pea protein isolate-high methoxyl pectin-EGCG complex: Interfacial properties and microstructure. Food Chem..

[55] Qin X.-S., Gao Q.-Y., Luo Z.-G. (2021). Enhancing the storage and gastrointestinal passage viability of probiotic powder (*Lactobacillus plantarum*) through encapsulation with pickering high internal phase emulsions stabilized with WPI-EGCG covalent conjugate nanoparticles. Food Hydrocoll..

[56] Miao J., Xu N., Cheng C., Zou L., Chen J., Wang Y., Liang R., McClements D.J., Liu W. (2021). Fabrication of polysaccharide-based high internal phase emulsion gels: Enhancement of curcumin stability and bioaccessibility. Food Hydrocoll..

[57] Yi J., Gan C., Wen Z., Fan Y., Wu X. (2021). Development of pea protein and high methoxyl pectin colloidal particles stabilized high internal phase pickering emulsions for β-carotene protection and delivery. Food Hydrocoll..

[58] Wu X., Xu N., Cheng C., McClements D.J., Chen X., Zou L., Liu W. (2022). Encapsulation of hydrophobic capsaicin within the aqueous phase of water-in-oil high internal phase emulsions: Controlled release, reduced irritation, and enhanced bioaccessibility. Food Hydrocoll..

[59] Gao Y., Wu X., McClements D.J., Cheng C., Xie Y., Liang R., Liu J., Zou L., Liu W. (2022). Encapsulation of bitter peptides in water-in-oil high internal phase emulsions reduces their bitterness and improves gastrointestinal stability. Food Chem..

[60] Zhang Y., Liu H., McClements D.J., Xie Y., Cheng C., Zou L., Liu W., Liu W. (2022). Probiotic encapsulation in water-in-oil high internal phase emulsions: Enhancement of viability under food and gastrointestinal conditions. LWT.

[61] Zhang L., Zheng J., Wang Y., Ye X., Chen S., Pan H., Chen J. (2022). Fabrication of rhamnogalacturonan-I enriched pectin-based emulsion gels for protection and sustained release of curcumin. Food Hydrocoll..

[62] Moayedzadeh S., Gunasekaran S., Madadlou A. (2022). Emulsion gels loaded with pancreatic lipase: Preparation from spontaneously made emulsions and assessment of the rheological, microscopic and cargo release properties. Food Res. Int..

[63] Zhang Y., Zhong Q. (2017). Solid-in-Oil-in-Water Emulsions for Delivery of Lactase to Control in Vitro Hydrolysis of Lactose in Milk. J. Agric. Food Chem..

[64] Koohenjani D.K., Lashkari H. (2022). Effects of double emulsion encapsulated iron on the properties of fortified cream. LWT.

[65] Han L., Lu K., Zhou S., Qi B., Li Y. (2022). Co-delivery of insulin and quercetin in W/O/W double emulsions stabilized by different hydrophilic emulsifiers. Food Chem..

[66] Marefati A., Pitsiladis A., Oscarsson E., Ilestam N., Bergenståhl B. (2021). Encapsulation of Lactobacillus reuteri in W1/O/W2 double emulsions: Formulation, storage and in vitro gastro-intestinal digestion stability. LWT.

[67] Shi A., Wang J., Guo R., Feng X., Ge Y., Liu H., Agyei D., Wang Q. (2021). Improving resveratrol bioavailability using water-in-oil-in-water (W/O/W) emulsion: Physicochemical stability, in vitro digestion resistivity and transport properties. J. Funct. Foods.

[68] Huang H., Wang D., Belwal T., Dong L., Lu L., Zou Y., Li L., Xu Y., Luo Z. (2021). A novel W/O/W double emulsion co-delivering brassinolide and cinnamon essential oil delayed the senescence of broccoli via regulating chlorophyll degradation and energy metabolism. Food Chem..

[69] Liu J., Zhou H., Mundo J.L.M., Tan Y., Pham H., McClements D.J. (2020). Fabrication and characterization of W/O/W emulsions with crystalline lipid phase. J. Food Eng..

[70] Li M., Bi D., Yao L., Yi J., Fang W., Wu Y., Xu H., Hu Z., Xu X. (2021). Optimization of preparation conditions and in vitro sustained-release evaluation of a novel nanoemulsion encapsulating unsaturated guluronate oligosaccharide. Carbohydr. Polym..

[71] Jiang H., Zhang T., Smits J., Huang X., Maas M., Yin S., Ngai T. (2021). Edible high internal phase Pickering emulsion with double-emulsion morphology. Food Hydrocoll..

[72] Bai L., Huan S., Rojas O.J., McClements D.J. (2021). Recent innovations in emulsion science and technology for food applications. J. Agric. Food Chem..

[73] Wan L., Li L., Harro J.M., Hoag S.W., Li B., Zhang X., Shirtliff M.E. (2020). In vitro gastrointestinal digestion of palm olein and palm stearin-in-water emulsions with different physical states and fat contents. J. Agric. Food Chem..

[74] Jiang T., Charcosset C. (2022). Encapsulation of curcumin within oil-in-water emulsions prepared by premix membrane emulsification: Impact of droplet size and carrier oil type on physicochemical stability and in vitro bioaccessibility. Food Chem..

[75] Guo X., Sun X.-T., Liang L., Shi L.-K., Liu R.-J., Chang M., Wang X.-G. (2021). Physical Stability, Oxidative Stability, and Bioactivity of Nanoemulsion Delivery Systems Incorporating Lipophilic Ingredients: Impact of Oil Saturation Degree. J. Agric. Food Chem..

[76] Tan Y., Zhou H., Zhang Z., McClements D.J. (2021). Bioaccessibility of oil-soluble vitamins (A, D, E) in plant-based emulsions: Impact of oil droplet size. Food Funct..

[77] Meng R., Wu Z., Xie Q.-T., Zhang B., Li X.-L., Liu W.-J., Tao H., Li P.-J. (2020). Zein/carboxymethyl dextrin nanoparticles stabilized pickering emulsions as delivery vehicles: Effect of interfacial composition on lipid oxidation and in vitro digestion. Food Hydrocoll..

[78] Chen M., Li W., Wang W., Cao Y., Lan Y., Huang Q., Xiao J. (2022). Effects of gelation on the stability, tribological properties and time-delayed release profile of double emulsions. Food Hydrocoll..

[79] Sun R., Xia Q. (2020). In vitro digestion behavior of (W1/O/W2) double emulsions incorporated in alginate hydrogel beads: Microstructure, lipolysis, and release. Food Hydrocoll..

[80] Fardous J., Omoso Y., Joshi A., Yoshida K., Patwary M.K.A., Ono F., Ijima H. (2021). Development and characterization of gel-in-water nanoemulsion as a novel drug delivery system. Mater. Sci. Eng. C.

[81] Palla C.A., Aguilera-Garrido A., Carrín M.E., Galisteo-González F., Gálvez-Ruiz M.J. (2022). Preparation of highly stable oleogel-based nanoemulsions for encapsulation and controlled release of curcumin. Food Chem..

[82] Qiu C., Wang J., Qin Y., Xu X., Jin Z. (2018). Characterization and mechanisms of novel emulsions and nanoemulsion gels stabilized by edible cyclodextrin-based metal–organic frameworks and glycyrrhizic acid. J. Agric. Food Chem..

[83] Cai X., Wang Y., Du X., Xing X., Zhu G. (2020). Stability of pH-responsive Pickering emulsion stabilized by carboxymethyl starch/xanthan gum combinations. Food Hydrocoll..

[84] Wu X., Zhang Q., Wang Z., Xu Y., Tao Q., Wang J., Kong X., Sheng K., Wang Y. (2022). Investigation of construction and characterization of carboxymethyl chitosan-sodium alginate nanoparticles to stabilize Pickering emulsion hydrogels for curcumin encapsulation and accelerating wound healing. Int. J. Biol. Macromol..

[85] Du Le H., Loveday S.M., Nowak E., Niu Z., Singh H. (2020). Pectin emulsions for colon-targeted release of propionic acid. Food Hydrocoll..

[86] Nunes R., Pereira B.D.A., Cerqueira M.A., Silva P., Pastrana L.M., Vicente A.A., Martins J.T., Bourbon A.I. (2020). Lactoferrin-based nanoemulsions to improve the physical and chemical stability of omega-3 fatty acids. Food Funct..

[87] Felix M., Guerrero A., Carrera-Sánchez C. (2022). Optimization of Multiple W1/O/W2 Emulsions Processing for Suitable Stability and Encapsulation Efficiency. Foods.

[88] Mohamad W.F.W., Buckow R., Augustin M., McNaughton D. (2017). In situ quantification of β-carotene partitioning in oil-in-water emulsions by confocal Raman microscopy. Food Chem..

[89] Tang X.-M., Liu P.-D., Chen Z.-J., Li X.-Y., Huang R., Liu G.-D., Dong R.-S., Chen J. (2022). Encapsulation of a *Desmodium intortum* Protein Isolate Pickering Emulsion of β-Carotene: Stability, Bioaccesibility and Cytotoxicity. Foods.

[90] Tan Y., Li R., Zhou H., Liu J., Mundo J.L.M., Zhang R., Mcclements D.J. (2020). Impact of calcium levels on lipid digestion and nutraceutical bioaccessibility in nanoemulsion delivery systems studied using standardized INFOGEST digestion protocol. Food Funct..

[91] Tan Y., Zhang Z., Zhou H., Xiao H., McClements D.J. (2020). Factors impacting lipid digestion and β-carotene bioaccessibility assessed by standardized gastrointestinal model (INFOGEST): Oil droplet concentration. Food Funct..

[92] Kharat M., Aberg J., Dai T., McClements D.J. (2020). Comparison of emulsion and nanoemulsion delivery systems: The chemical stability of curcumin decreases as oil droplet size decreases. J. Agric. Food Chem..

[93] Silva K.C.G., Bourbon A.I., Pastrana L., Sato A.C.K. (2021). Biopolymer interactions on emulsion-filled hydrogels: Chemical, mechanical properties and microstructure. Food Res. Int..

[94] Catalkaya G., Venema K., Lucini L., Rocchetti G., Delmas D., Daglia M., De Filippis A., Xiao H., Quiles J.L., Xiao J. (2020). Interaction of dietary polyphenols and gut microbiota: Microbial metabolism of polyphenols, influence on the gut microbiota, and implications on host health. Food Front..

[95] Zhao X., Zhang X., Liu H., Zhang G., Ao Q. (2018). Functional, nutritional and flavor characteristic of soybean proteins obtained through reverse micelles. Food Hydrocoll..

[96] Zhao X., Liu H., Zhang X., Zhu H., Ao Q. (2019). Surface structure and volatile characteristic of peanut proteins obtained through AOT reverse micelles. Colloids Surf. B Biointerfaces.

[97] Leite A.C., Ferreira A.M., Morais E.S., Khan I., Freire M.G., Coutinho J.A. (2018). Cloud point extraction of chlorophylls from spinach leaves using aqueous solutions of nonionic surfactants. ACS Sustain. Chem. Eng..

[98] Javed S., Ahsan W. (2019). Microemulsion based chromatographic techniques: Past lessons and future directions. J. Liq. Chromatogr. Relat. Technol..

[99] Zhang G., Huang B., Zheng C., Chen Q., Fei P. (2020). Investigation of a lipase-catalyzed reaction between pectin and salicylic acid and its isomers and evaluation of the emulsifying properties, antioxidant activities, and antibacterial activities of the corresponding products. J. Agric. Food Chem..

[100] Qu Y., Sun D., Yu Y. (2022). Interfacial engineering in Pickering emulsion photocatalytic microreactors: From mechanisms to prospects. Chem. Eng. J..

[101] Dong Z., Liu Z., Shi J., Tang H., Xiang X., Huang F., Zheng M. (2019). Carbon Nanoparticle-Stabilized Pickering Emulsion as a Sustainable and High-Performance Interfacial Catalysis Platform for Enzymatic Esterification/Transesterification. ACS Sustain. Chem. Eng..

[102] Sun T., Dong Z., Wang J., Huang F.-H., Zheng M.-M. (2020). Ultrasound-assisted interfacial immobilization of lipase on hollow mesoporous silica spheres in a pickering emulsion system: A hyperactive and sustainable biocatalyst. ACS Sustain. Chem. Eng..

[103] Xu L.-J., Yang T., Wang J., Huang F.-H., Zheng M.-M. (2021). Immobilized lipase based on hollow mesoporous silicon spheres for efficient enzymatic synthesis of resveratrol ester derivatives. J. Agric. Food Chem..

[104] Li Z., Shi Y., Zhu A., Zhao Y., Wang H., Binks B.P., Wang J. (2021). Light-Responsive, reversible emulsification and demulsification of oil-in-water pickering emulsions for catalysis. Angew. Chem..

[105] Xi Y., Liu B., Wang S., Huang X., Jiang H., Yin S., Ngai T., Yang X. (2021). Growth of Au nanoparticles on phosphorylated zein protein particles for use as biomimetic catalysts for cascade reactions at the oil–water interface. Chem. Sci..

[106] Xi Y., Liu B., Jiang H., Yin S., Ngai T., Yang X. (2020). Sodium caseinate as a particulate emulsifier for making indefinitely recycled pH-responsive emulsions. Chem. Sci..

[107] Wang M., Wang M., Zhang S., Chen J. (2019). Pickering gel emulsion stabilized by enzyme immobilized polymeric nanoparticles: A robust and recyclable biocatalyst system for biphasic catalysis. React. Chem. Eng..

[108] Jiao Y.-J., Yuan F.-F., Fan P.-R., Wei Z.-H., Huang Y.-P., Liu Z.-S. (2021). Macroporous monolithic enzyme microreactor based on high internal phase emulsion functionalized with gold nanorods for enzymatic hydrolysis of protein. Chem. Eng. J..

[109] Li R., Xue H., Gao B., Liu H., Han T., Hu X., Tu Y., Zhao Y. (2022). Physicochemical properties and digestibility of thermally induced ovalbumin–oil emulsion gels: Effect of interfacial film type and oil droplets size. Food Hydrocoll..

[110] Infantes-Garcia M.R., Verkempinck S., Gonzalez-Fuentes P., Hendrickx M., Grauwet T. (2021). Lipolysis products formation during in vitro gastric digestion is affected by the emulsion interfacial composition. Food Hydrocoll..

[111] Ni Y., Gu Q., Li J., Fan L. (2021). Modulating in vitro gastrointestinal digestion of nanocellulose-stabilized pickering emulsions by altering cellulose lengths. Food Hydrocoll..

[112] Martínez S., Espert M., Salvador A., Sanz T. (2022). The role of oil concentration on the rheological properties, microstructure, and in vitro digestion of cellulose ether emulsions. Food Hydrocoll..

[113] Chen S., Du Y., Zhang H., Wang Q., Gong Y., Chang R., Zhang J., Zhang J., Yuan Y., Liu B. (2022). The lipid digestion behavior of oil-in-water Pickering emulsions stabilized by whey protein microgels of various rigidities. Food Hydrocoll..

[114] Zhang X., Wu Y., Li Y., Li B., Pei Y., Liu S. (2022). Effects of the interaction between bacterial cellulose and soy protein isolate on the oil-water interface on the digestion of the Pickering emulsions. Food Hydrocoll..

[115] Santos T.P., Okuro P.K., Cunha R.L. (2021). Pickering emulsions as a platform for structures design: Cutting-edge strategies to engineer digestibility. Food Hydrocoll..

[116] Naso J.N., Bellesi F.A., Ruiz-Henestrosa V.M.P., Pilosof A.M. (2019). Studies on the interactions between bile salts and food emulsifiers under in vitro duodenal digestion conditions to evaluate their bile salt binding potential. Colloids Surf. B Biointerfaces.

[117] Sarkar A., Zhang S., Holmes M., Ettelaie R. (2019). Colloidal aspects of digestion of Pickering emulsions: Experiments and theoretical models of lipid digestion kinetics. Adv. Colloid Interface Sci..

[118] Chen L., Yokoyama W., Liang R., Zhong F. (2020). Enzymatic degradation and bioaccessibility of protein encapsulated β-carotene nano-emulsions during in vitro gastro-intestinal digestion. Food Hydrocoll..

[119] Zhou F., Yan L., Yin S., Tang C., Yang X. (2018). Development of Pickering Emulsions Stabilized by Gliadin/Proanthocyanidins Hybrid Particles (GPHPs) and the Fate of Lipid Oxidation and Digestion. J. Agric. Food Chem..

[120] Zhao M., Shen P., Zhang Y., Zhong M., Zhao Q., Zhou F. (2021). Fabrication of soy protein nanoparticles via partial enzymatic hydrolysis and their role in controlling lipid digestion of oil-in-water emulsions. ACS Food Sci. Technol..

[121] Wen J., Zhang Y., Jin H., Sui X., Jiang L. (2020). Deciphering the Structural Network That Confers Stability to High Internal Phase Pickering Emulsions by Cross-Linked Soy Protein Microgels and Their In Vitro Digestion Profiles. J. Agric. Food Chem..

[122] Zhu X., Wang Q., Leng Y., Chen F., Wu F., Mu G., Wu X. (2021). Lecithin alleviates protein flocculation and enhances fat digestion in a model of infant formula emulsion. Food Chem..

[123] Liang L., Zhang X., Wang X., Jin Q., Mcclements D.J. (2018). Influence of Dairy Emulsifier Type and Lipid Droplet Size on Gastrointestinal Fate of Model Emulsions: In Vitro Digestion Study. J. Agric. Food Chem..

[124] Wan L., Li L., Xiao J., He N., Zhang R., Li B., Zhang X. (2022). The interfacial digestion behavior of crystalline oil-in-water emulsions stabilized by sodium caseinate during in vitro gastrointestinal digestion. Food Hydrocoll..

[125] Jiao W., Li L., Yu A., Zan S., Chen Z., Liang Y., Liang K., Li B., Zhang X. (2022). Modulating the in vitro gastrointestinal digestibility of crystalline oil-in-water emulsion: Different fat crystal sizes and polymorphic forms under the same SFC. Food Chem..

[126] Li W., Wang W., Yong C., Lan Y., Huang Q., Xiao J. (2022). Effects of the Distribution Site of Crystallizable Emulsifiers on the Gastrointestinal Digestion Behavior of Double Emulsions. J. Agric. Food Chem..

[127] Dong L., Lv M., Gao X., Zhang L., Rogers M., Cao Y., Lan Y. (2020). In vitro gastrointestinal digestibility of phytosterol oleogels: Influence of self-assembled microstructures on emulsification efficiency and lipase activity. Food Funct..

[128] Kang J., Park S., Park J.B., Song K.B. (2019). Surfactant type affects the washing effect of cinnamon leaf essential oil emulsion on kale leaves. Food Chem..

[129] Zhang X., Liang H., Li J., Li B. (2022). Fabrication of processable and edible high internal phase Pickering emulsions stabilized with gliadin/sodium carboxymethyl cellulose colloid particles. Food Hydrocoll..

[130] Cui F., McClements D.J., Liu X., Liu F., Ngai T. (2022). Development of pH-responsive emulsions stabilized by whey protein fibrils. Food Hydrocoll..

[131] Courtenay J.C., Jin Y., Schmitt J., Hossain K.M.Z., Mahmoudi N., Edler K.J., Scott J.L. (2021). Salt-Responsive Pickering Emulsions Stabilized by Functionalized Cellulose Nanofibrils. Langmuir.

[132] Tan C., Pajoumshariati S., Arshadi M., Abbaspourrad A. (2019). A simple route to renewable high internal phase emulsions (HIPEs) strengthened by successive cross-linking and electrostatics of polysaccharides. Chem. Commun..

[133] Tie S., Su W., Zhang X., Chen Y., Zhao X., Tan M. (2021). pH-Responsive core–shell microparticles prepared by a microfluidic chip for the encapsulation and controlled release of procyanidins. J. Agric. Food Chem..

[134] Sun H., Li L., Russell T.P., Shi S. (2020). Photoresponsive structured liquids enabled by molecular recognition at liquid–liquid interfaces. J. Am. Chem. Soc..

[135] Hashemnejad S.M., Badruddoza A.Z.M., Zarket B., Ricardo Castaneda C., Doyle P.S. (2019). Thermoresponsive nanoemulsion-based gel synthesized through a low-energy process. Nat. Commun..

[136] Jiang Y., Chakroun R., Gu P., Gröschel A.H., Russell T.P. (2020). Soft polymer Janus nanoparticles at liquid–liquid interfaces. Angew. Chem..

[137] Ku K.H., Li J., Yoshinaga K., Swager T.M. (2019). Dynamically Reconfigurable, Multifunctional Emulsions with Controllable Structure and Movement. Adv. Mater..

[138] Zhong M., Xie F., Zhang S., Sun Y., Qi B., Li Y. (2020). Preparation and digestive characteristics of a novel soybean lipophilic protein-hydroxypropyl methylcellulose-calcium chloride thermosensitive emulsion gel. Food Hydrocoll..

[139] Rodríguez-Arco L., Li M., Mann S. (2017). Phagocytosis-inspired behaviour in synthetic protocell communities of compartmentalized colloidal objects. Nat. Mater..

[140] Zhu Q., Liu S., Sun J., Liu J., Kirubaharan C.J., Chen H., Xu W., Wang Q. (2020). Stimuli-responsive cellulose nanomaterials for smart applications. Carbohydr. Polym..

[141] Infantes-Garcia M.R., Verkempinck S.H., Hendrickx M.E., Grauwet T. (2021). Kinetic modeling of in vitro small intestinal lipid digestion as affected by the emulsion interfacial composition and gastric prelipolysis. J. Agric. Food Chem..

[142] Infantes-Garcia M.R., Verkempinck S.H., Del Castillo-Santaella T., Maldonado-Valderrama J., Hendrickx M.E., Grauwet T. (2022). In vitro gastric lipid digestion of emulsions with mixed emulsifiers: Correlation between lipolysis kinetics and interfacial characteristics. Food Hydrocoll..

[143] Zhang T., Ding M., Zhang H., Tao N., Wang X., Zhong J. (2020). Fish oil-loaded emulsions stabilized by synergetic or competitive adsorption of gelatin and surfactants on oil/water interfaces. Food Chem..

[144] Wei Y., Tong Z., Dai L., Wang D., Lv P., Liu J., Mao L., Yuan F., Gao Y. (2020). Influence of interfacial compositions on the microstructure, physiochemical stability, lipid digestion and β-carotene bioaccessibility of Pickering emulsions. Food Hydrocoll..

[145] Wei Y., Zhou D., Mackie A., Yang S., Dai L., Zhang L., Mao L., Gao Y. (2021). Stability, interfacial structure, and gastrointestinal digestion of β-carotene-loaded pickering emulsions co-stabilized by particles, a biopolymer, and a surfactant. J. Agric. Food Chem..

[146] Zheng L., Cao C., Chen Z., Cao L., Huang Q., Song B. (2020). Evaluation of emulsion stability by monitoring the interaction between droplets. LWT.

[147] Yan S., Xu J., Zhang S., Zhu H., Qi B., Li Y. (2022). Effect of interfacial composition on the physical stability and co-oxidation of proteins and lipids in a soy protein isolate-(−)-epigallocatechin gallate conjugate emulsion. Food Hydrocoll..

[148] Tian L., Zhang S., Yi J., Zhu Z., Li M., Decker E.A., McClements D.J. (2021). Formation of Antioxidant Multilayered Coatings for the Prevention of Lipid and Protein Oxidation in Oil-in-Water Emulsions: *Lycium barbarum* Polysaccharides and Whey Proteins. J. Agric. Food Chem..

[149] Song H.Y., Moon T.W., Choi S.J. (2019). Impact of antioxidant on the stability of β-carotene in model beverage emulsions: Role of emulsion interfacial membrane. Food Chem..

[150] Feng T., Wang X., Wang X., Xia S., Huang Q. (2021). Plant protein-based antioxidant Pickering emulsions and high internal phase Pickering emulsions against broad pH range and high ionic strength: Effects of interfacial rheology and microstructure. LWT.

[151] Wang Z., Ma Y., Chen H., Deng Y., Wei Z., Zhang Y., Tang X., Li P., Zhao Z., Zhou P. (2022). Rice bran-modified wheat gluten nanoparticles effectively stabilized pickering emulsion: An interfacial antioxidant inhibiting lipid oxidation. Food Chem..

[152] Chen J., Li X., Cao C., Kong B., Wang H., Zhang H., Liu Q. (2022). Effects of different pH conditions on interfacial composition and protein-lipid co-oxidation of whey protein isolate-stabilised O/W emulsions. Food Hydrocoll..

[153] Yesiltas B., García-Moreno P.J., Gregersen S., Olsen T.H., Jones N.C., Hoffmann S.V., Marcatili P., Overgaard M.T., Hansen E.B., Jacobsen C. (2022). Antioxidant peptides derived from potato, seaweed, microbial and spinach proteins: Oxidative stability of 5% fish oil-in-water emulsions. Food Chem..

[154] Xu M., Lian Z., Chen X., Yao X., Lu C., Niu X., Xu M., Zhu Q. (2021). Effects of resveratrol on lipid and protein co-oxidation in fish oil-enriched whey protein isolate emulsions. Food Chem..

[155] Zou Y.-C., Wu C.-L., Ma C.-F., He S., Brennan C.S., Yuan Y. (2019). Interactions of grape seed procyanidins with soy protein isolate: Contributing antioxidant and stability properties. LWT.

[156] Feng J., Berton-Carabin C.C., Fogliano V., Schroën K. (2022). Maillard reaction products as functional components in oil-in-water emulsions: A review highlighting interfacial and antioxidant properties. Trends Food Sci. Technol..

[157] Bravo-Díaz C. (2022). Advances in the control of lipid peroxidation in oil-in-water emulsions: Kinetic approaches. Crit. Rev. Food Sci. Nutr..

[158] Daoud S., Bou-Maroun E., Waschatko G., Cayot P. (2021). Lipid oxidation in oil-in-water emulsions: Iron complexation by buffer ions and transfer on the interface as a possible mechanism. Food Chem..

[159] Da Silveira T.F.F., Laguerre M., Bourlieu-Lacanal C., Lecomte J., Durand E., Figueroa-Espinoza M.C., Baréa B., Barouh N., Castro I.A., Villeneuve P. (2021). Impact of surfactant concentration and antioxidant mode of incorporation on the oxidative stability of oil-in-water nanoemulsions. LWT.

[160] Yi J., Qiu M., Liu N., Tian L., Zhu X., Decker E.A., McClements D.J. (2020). Inhibition of lipid and protein oxidation in whey-protein-stabilized emulsions using a natural antioxidant: Black rice anthocyanins. J. Agric. Food Chem..

[161] Yin X., Dong H., Cheng H., Ji C., Liang L. (2022). Sodium caseinate particles with co-encapsulated resveratrol and epigallocatechin-3-gallate for inhibiting the oxidation of fish oil emulsions. Food Hydrocoll..

[162] Wang P., Liu S. (2020). Development of a Gemini Interfacial Antioxidant for Oil in Water Emulsion with Gallic Acid and Dodecyl Gemini Chains. J. Agric. Food Chem..

[163] Cao X., Xiong C., Zhao X., Yang S., Wen Q., Tang H., Zeng Q., Feng Y., Li J. (2022). Tuning self-assembly of amphiphilic sodium alginate-decorated selenium nanoparticle surfactants for antioxidant Pickering emulsion. Int. J. Biol. Macromol..

[164] Mouafo H.T., Mbawala A., Tanaji K., Somashekar D., Ndjouenkeu R. (2020). Improvement of the shelf life of raw ground goat meat by using biosurfactants produced by lactobacilli strains as biopreservatives. LWT.

[165] Schröder A., Laguerre M., Tenon M., Schroën K., Berton-Carabin C.C. (2021). Natural particles can armor emulsions against lipid oxidation and coalescence. Food Chem..

[166] Eisinaite V., Juraite D., Schroën K., Leskauskaite D. (2017). Food-grade double emulsions as effective fat replacers in meat systems. J. Food Eng..

[167] Lee M.C., Tan C., Ravanfar R., Abbaspourrad A. (2019). Ultra-Stable Water-in-Oil High Internal Phase Emulsions Featuring Interfacial and Biphasic Network Stabilization. ACS Appl. Mater. Interfaces.

[168] Heymans R., Tavernier I., Dewettinck K., Van der Meeren P. (2017). Crystal stabilization of edible oil foams. Trends Food Sci. Technol..

[169] Li G., Lee W.J., Liu N., Lu X., Tan C.P., Lai O.M., Qiu C., Wang Y. (2021). Stabilization mechanism of water-in-oil emulsions by medium-and long-chain diacylglycerol: Post-crystallization vs. pre-crystallization. LWT.

[170] Lei M., Zhang N., Lee W.J., Tan C.P., Lai O.M., Wang Y., Qiu C. (2020). Non-aqueous foams formed by whipping diacylglycerol stabilized oleogel. Food Chem..

[171] Qiu C., Lei M., Lee W.J., Zhang N., Wang Y. (2021). Fabrication and characterization of stable oleofoam based on medium-long chain diacylglycerol and β-sitosterol. Food Chem..

[172] Yang J., Qiu C., Li G., Lee W.J., Tan C.P., Lai O.M., Wang Y. (2020). Effect of diacylglycerol interfacial crystallization on the physical stability of water-in-oil emulsions. Food Chem..

[173] Peng L.-P., Tang C.-H. (2020). Outstanding antioxidant pickering high internal phase emulsions by co-assembled polyphenol-soy β-conglycinin nanoparticles. Food Res. Int..

[174] Wang Q., Jiang J., Xiong Y.L. (2019). Genipin-Aided Protein Cross-linking to Modify Structural and Rheological Properties of Emulsion-Filled Hempseed Protein Hydrogels. J. Agric. Food Chem..

[175] Huang Z.-X., Lin W.-F., Zhang Y., Tang C.-H. (2022). Freeze-thaw-stable High Internal Phase Emulsions Stabilized by Soy Protein Isolate and Chitosan Complexes at pH 3.0 as Promising Mayonnaise Replacers. Food Res. Int..

[176] Liu X., Guo J., Wan Z.-L., Liu Y.-Y., Ruan Q.-J., Yang X.-Q. (2018). Wheat gluten-stabilized high internal phase emulsions as mayonnaise replacers. Food Hydrocoll..

[177] Ruan Q., Yang X., Zeng L., Qi J. (2019). Physical and tribological properties of high internal phase emulsions based on citrus fibers and corn peptides. Food Hydrocoll..

[178] Lu Z., Zhou S., Ye F., Zhou G., Gao R., Qin D., Zhao G. (2021). A novel cholesterol-free mayonnaise made from Pickering emulsion stabilized by apple pomace particles. Food Chem..

[179] Hosseini R.S., Rajaei A. (2020). Potential Pickering emulsion stabilized with chitosan-stearic acid nanogels incorporating clove essential oil to produce fish-oil-enriched mayonnaise. Carbohydr. Polym..

[180] Li S., Jiao B., Meng S., Fu W., Faisal S., Li X., Liu H., Wang Q. (2022). Edible mayonnaise-like Pickering emulsion stabilized by pea protein isolate microgels: Effect of food ingredients in commercial mayonnaise recipe. Food Chem..

[181] Jiao B., Shi A., Wang Q., Binks B.P. (2018). High-internal-phase pickering emulsions stabilized solely by peanut-protein-isolate microgel particles with multiple potential applications. Angew. Chem..

[182] Li X.-L., Meng R., Xu B.-C., Zhang B., Cui B., Wu Z.-Z. (2022). Function emulsion gels prepared with carrageenan and zein/carboxymethyl dextrin stabilized emulsion as a new fat replacer in sausages. Food Chem..

[183] Paglarini C.d.S., Vidal V.A.S., Martini S., Cunha R.L., Pollonio M.A.R. (2022). Protein-based hydrogelled emulsions and their application as fat replacers in meat products: A review. Crit. Rev. Food Sci. Nutr..

[184] Wang C., Wang X., Liu C., Liu C. (2021). Application of LF-NMR to the characterization of camellia oil-loaded pickering emulsion fabricated by soy protein isolate. Food Hydrocoll..

[185] Pan H., Xu X., Qian Z., Cheng H., Shen X., Chen S., Ye X. (2021). Xanthan gum-assisted fabrication of stable emulsion-based oleogel structured with gelatin and proanthocyanidins. Food Hydrocoll..

[186] Yang Y., Zhang M., Li J., Su Y., Gu L., Yang Y., Chang C. (2022). Construction of egg white protein particle and rhamnolipid based emulsion gels with β-sitosterol as gelation factor: The application in cookie. Food Hydrocoll..

[187] Kwon H.C., Shin D.-M., Yune J.H., Jeong C.H., Han S.G. (2021). Evaluation of gels formulated with whey proteins and sodium dodecyl sulfate as a fat replacer in low-fat sausage. Food Chem..

[188] Pintado T., Muñoz-González I., Salvador M., Ruiz-Capillas C., Herrero A.M. (2021). Phenolic compounds in emulsion gel-based delivery systems applied as animal fat replacers in frankfurters: Physico-chemical, structural and microbiological approach. Food Chem..

[189] Hu X., McClements D.J. (2022). Construction of plant-based adipose tissue using high internal phase emulsions and emulsion gels. Innov. Food Sci. Emerg. Technol..

[190] Cao M., Zhang X., Zhu Y., Liu Y., Ma L., Chen X., Zou L., Liu W. (2022). Enhancing the physicochemical performance of myofibrillar gels using Pickering emulsion fillers: Rheology, microstructure and stability. Food Hydrocoll..

[191] Hu S., Wu J., Zhu B., Du M., Wu C., Yu C., Song L., Xu X. (2021). Low oil emulsion gel stabilized by defatted Antarctic krill (*Euphausia superba*) protein using high-intensity ultrasound. Ultrason. Sonochem..

[192] Du C.-X., Xu J.-J., Luo S.-Z., Li X.-J., Mu D.-D., Jiang S.-T., Zheng Z. (2022). Low-oil-phase emulsion gel with antioxidant properties prepared by soybean protein isolate and curcumin composite nanoparticles. LWT.

[193] Gutiérrez-Luna K., Astiasarán I., Ansorena D. (2020). Gels as fat replacers in bakery products: A review. Crit. Rev. Food Sci. Nutr..

[194] Grossmann L., Kinchla A.J., Nolden A., McClements D.J. (2021). Standardized methods for testing the quality attributes of plant-based foods: Milk and cream alternatives. Compr. Rev. Food Sci. Food Saf..

[195] Wang Y., Zhao J., Zhang S., Zhao X., Liu Y., Jiang J., Xiong Y.L. (2022). Structural and rheological properties of mung bean protein emulsion as a liquid egg substitute: The effect of pH shifting and calcium. Food Hydrocoll..

[196] Lan M., Zheng J., Huang C., Wang Y., Hu W., Lu S., Liu F., Ou S. (2022). Water-In-Oil Pickering Emulsions Stabilized by Microcrystalline Phytosterols in Oil: Fabrication Mechanism and Application as a Salt Release System. J. Agric. Food Chem..

[197] Wang X., Ullah N., Shen Y., Sun Z., Wang X., Feng T., Zhang X., Huang Q., Xia S. (2021). Emulsion delivery of sodium chloride: A promising approach for modulating saltiness perception and sodium reduction. Trends Food Sci. Technol..

[198] Sun C., Zhou X., Hu Z., Lu W., Zhao Y., Fang Y. (2021). Food and salt structure design for salt reducing. Innov. Food Sci. Emerg. Technol..

[199] Ahsan H.M., Pei Y., Luo X., Wang Y., Li Y., Li B., Liu S. (2020). Novel stable pickering emulsion based solid foams efficiently stabilized by microcrystalline cellulose/chitosan complex particles. Food Hydrocoll..

[200] Zhang X., Zhou J., Chen J., Li B., Li Y., Liu S. (2020). Edible foam based on pickering effect of bacterial cellulose nanofibrils and soy protein isolates featuring interfacial network stabilization. Food Hydrocoll..

[201] Wang Y., Hartel R.W., Zhang L. (2021). The stability of aerated emulsions: Effects of emulsifier synergy on partial coalescence and crystallization of milk fat. J. Food Eng..

[202] Hu Y., Shi L., Ren Z., Hao G., Chen J., Weng W. (2021). Characterization of emulsion films prepared from soy protein isolate at different preheating temperatures. J. Food Eng..

[203] Guo Y., Wei Y., Cai Z., Hou B., Zhang H. (2021). Stability of acidified milk drinks induced by various polysaccharide stabilizers: A review. Food Hydrocoll..

[204] Du Q., Tang J., Xu M., Lyu F., Zhang J., Qiu Y., Liu J., Ding Y. (2021). Whey protein and maltodextrin-stabilized oil-in-water emulsions: Effects of dextrose equivalent. Food Chem..

[205] Wu K., Shi Z., Liu C., Su C., Zhang S., Yi F. (2022). Preparation of Pickering emulsions based on soy protein isolate-tannic acid for protecting aroma compounds and their application in beverages. Food Chem..

[206] Li K.-Y., Zhou Y., Huang G.-Q., Li X.-D., Xiao J.-X. (2022). Preparation of powdered oil by spray drying the Pickering emulsion stabilized by ovalbumin–gum Arabic polyelectrolyte complex. Food Chem..

[207] Xu W., Sun H., Li H., Li Z., Zheng S., Luo D., Ning Y., Wang Y., Shah B.R. (2022). Preparation and characterization of tea oil powder with high water solubility using Pickering emulsion template and vacuum freeze-drying. LWT.

[208] Dun H., Liang H., Zhan F., Wei X., Chen Y., Wan J., Ren Y., Hu L., Li B. (2020). Influence of O/W emulsion on gelatinization and retrogradation properties of rice starch. Food Hydrocoll..

[209] Lee E.-s., Song H.-g., Choi I., Lee J.-S., Han J. (2020). Effects of mung bean starch/guar gum-based edible emulsion coatings on the staling and safety of rice cakes. Carbohydr. Polym..

[210] Chen L., Din Z.-u., Yang D., Hu C., Cai J., Xiong H. (2021). Functional nanoparticle reinforced starch-based adhesive emulsion: Toward robust stability and high bonding performance. Carbohydr. Polym..

[211] Zhu X., Li L., Li S., Ning C., Zhou C. (2019). l–Arginine/l–lysine improves emulsion stability of chicken sausage by increasing electrostatic repulsion of emulsion droplet and decreasing the interfacial tension of soybean oil-water. Food Hydrocoll..

[212] Fang Q., Shi L., Ren Z., Hao G., Chen J., Weng W. (2021). Effects of emulsified lard and TGase on gel properties of threadfin bream (Nemipterus virgatus) surimi. LWT.

[213] Xu L., Lv Y., Su Y., Chang C., Gu L., Yang Y., Li J. (2022). Enhancing gelling properties of high internal phase emulsion-filled chicken gels: Effect of droplet fractions and salts. Food Chem..

[214] Tao J., Zhu Q., Qin F., Wang M., Chen J., Zheng Z.-P. (2017). Preparation of steppogenin and ascorbic acid, vitamin E, butylated hydroxytoluene oil-in-water microemulsions: Characterization, stability, and antibrowning effects for fresh apple juice. Food Chem..

[215] Ai Y., Fang F., Zhang L., Liao H. (2022). Antimicrobial activity of oregano essential oil and resveratrol emulsions co-encapsulated by sodium caseinate with polysaccharides. Food Control.

[216] Feng X., Tjia J.Y.Y., Zhou Y., Liu Q., Fu C., Yang H. (2020). Effects of tocopherol nanoemulsion addition on fish sausage properties and fatty acid oxidation. LWT.

[217] Ceylan Z., Meral R., Kose S., Sengor G., Akinay Y., Durmus M., Ucar Y. (2020). Characterized nano-size curcumin and rosemary oil for the limitation microbial spoilage of rainbow trout fillets. LWT.

[218] Wang W., Zhao D., Xiang Q., Li K., Wang B., Bai Y. (2021). Effect of cinnamon essential oil nanoemulsions on microbiological safety and quality properties of chicken breast fillets during refrigerated storage. LWT.

[219] González-González C.R., Labo-Popoola O., Delgado-Pando G., Theodoridou K., Doran O., Stratakos A.C. (2021). The effect of cold atmospheric plasma and linalool nanoemulsions against Escherichia coli O157: H7 and Salmonella on ready-to-eat chicken meat. Lwt.

[220] Da Rosa C.G., de Melo A.P.Z., Sganzerla W.G., Machado M.H., Nunes M.R., Maciel M.V.d.O.B., Bertoldi F.C., Barreto P.L.M. (2020). Application in situ of zein nanocapsules loaded with *Origanum vulgare* Linneus and *Thymus vulgaris* as a preservative in bread. Food Hydrocoll..

[221] Christaki S., Moschakis T., Kyriakoudi A., Biliaderis C.G., Mourtzinos I. (2021). Recent advances in plant essential oils and extracts: Delivery systems and potential uses as preservatives and antioxidants in cheese. Trends Food Sci. Technol..

[222] Jafarzadeh S., Salehabadi A., Nafchi A.M., Oladzadabbasabadi N., Jafari S.M. (2021). Cheese packaging by edible coatings and biodegradable nanocomposites; improvement in shelf life, physicochemical and sensory properties. Trends Food Sci. Technol..

[223] Crowley S.V., Kelly A.L., Omahony J.A., Lucey J.A. (2019). Colloidal properties of protein complexes formed in β-casein concentrate solutions as influenced by heating and cooling in the presence of different solutes. Colloids Surf. B Biointerfaces.

[224] Chen M., Sagis L.M.C. (2019). The influence of protein/phospholipid ratio on the physicochemical and interfacial properties of biomimetic milk fat globules. Food Hydrocoll..

[225] Renhe I.R.T., Corredig M. (2018). Effect of partial whey protein depletion during membrane filtration on thermal stability of milk concentrates. J. Dairy Sci..

[226] Zhou H., Zheng B., Zhang Z., Zhang R., He L., McClements D.J. (2021). Fortification of plant-based milk with calcium may reduce vitamin D bioaccessibility: An in vitro digestion study. J. Agric. Food Chem..

[227] Lin X., Wright A.J. (2018). Pectin and gastric pH interactively affect DHA-rich emulsion in vitro digestion microstructure, digestibility and bioaccessibility. Food Hydrocoll..

[228] Hu Y., Liu F., Pang J., McClements D.J., Zhou Z., Li B., Li Y. (2020). Biopolymer additives enhance tangeretin bioavailability in emulsion-based delivery systems: An in vitro and in vivo study. J. Agric. Food Chem..

[229] Hu M., Liu G., Du X., Zhang X., Qi B., Li Y. (2020). Molecular crowding prevents the aggregation of protein-dextran conjugate by inducing structural changes, improves its functional properties, and stabilizes it in nanoemulsions. Int. J. Biol. Macromol..

[230] Fernandezavila C., Trujillo A.J. (2017). Enhanced stability of emulsions treated by Ultra-High Pressure Homogenization for delivering conjugated linoleic acid in Caco-2 cells. Food Hydrocoll..

[231] Singh C.K.S., Lim H.-P., Khoo J.Y.-P., Tey B.-T., Chan E.-S. (2022). Effects of high-energy emulsification methods and environmental stresses on emulsion stability and retention of tocotrienols encapsulated in Pickering emulsions. J. Food Eng..

[232] Aguilar C., Serna-Jiménez J., Benitez E., Valencia V., Ochoa O., Sotelo L. (2021). Influence of high power ultrasound on natural microflora, pathogen and lactic acid bacteria in a raw meat emulsion. Ultrason. Sonochem..

[233] Kashaninejad M., Razavi S.M.A. (2020). Influence of thermosonication treatment on the average size of fat globules, emulsion stability, rheological properties and color of camel milk cream. LWT.

[234] Tian L., Hu S., Jia J., Tan W., Yang L., Zhang Q., Liu X., Duan X. (2021). Effects of short-term fermentation with lactic acid bacteria on the characterization, rheological and emulsifying properties of egg yolk. Food Chem..

[235] Xie L., Shi C., Cui X., Zeng H. (2017). Surface forces and interaction mechanisms of emulsion drops and gas bubbles in complex fluids. Langmuir.

[236] Zhao L., Du M., Mao X. (2019). Change in interfacial properties of milk fat globules by homogenization and thermal processing plays a key role in their in vitro gastrointestinal digestion. Food Hydrocoll..

[237] Syed U.T., Leonardo I.s., Lahoz R., Gaspar F.d.r.B., Huertas R., Crespo M.T., Arruebo M., Crespo J., Sebastian V., Brazinha C. (2020). Microengineered Membranes for Sustainable Production of Hydrophobic Deep Eutectic Solvent-Based Nanoemulsions by Membrane Emulsification for Enhanced Antimicrobial Activity. ACS Sustain. Chem. Eng..

[238] Rodrigues S., Pradal C., Yu L., Steadman K., Stokes J., Yakubov G. (2021). Creating polysaccharide-protein complexes to control aqueous lubrication. Food Hydrocoll..

[239] Zhang Y., Wang Y., Zhang R., Yu J., Gao Y., Mao L. (2022). Tuning the rheological and tribological properties to simulate oral processing of novel high internal phase oleogel-in-water emulsions. Food Hydrocoll..

[240] Upadhyay R., Chen J. (2019). Smoothness as a tactile percept: Correlating ‘oral’tribology with sensory measurements. Food Hydrocoll..

[241] Yang N., Feng Y., Su C., Wang Q., Zhang Y., Wei Y., Zhao M., Nishinari K., Fang Y. (2020). Structure and tribology of κ-carrageenan gels filled with natural oil bodies. Food Hydrocoll..

[242] Semenova M., Antipova A., Martirosova E., Zelikina D., Palmina N., Chebotarev S. (2021). Essential contributions of food hydrocolloids and phospholipid liposomes to the formation of carriers for controlled delivery of biologically active substances via the gastrointestinal tract. Food Hydrocoll..

[243] Wang X., Wang X., Upadhyay R., Chen J. (2019). Topographic study of human tongue in relation to oral tribology. Food Hydrocoll..

[244] Soares S.n., Brandão E., Guerreiro C., Mateus N., de Freitas V., Soares S. (2019). Development of a new cell-based oral model to study the interaction of oral constituents with food polyphenols. J. Agric. Food Chem..

[245] Benoit D.S., Sims Jr K.R., Fraser D. (2019). Nanoparticles for oral biofilm treatments. ACS Nano.

[246] Ke L., Xu Y., Gao G., Wang H., Yu Z., Zhou J., Rao P., Wang Q., Yu J. (2020). Catalase to demulsify oil-in-water fish oil-polysorbate emulsion and affect lipid oxidation. Food Res. Int..

[247] Siemer S., Hahlbrock A., Vallet C., McClements D.J., Balszuweit J., Voskuhl J., Docter D., Wessler S., Knauer S.K., Westmeier D. (2018). Nanosized food additives impact beneficial and pathogenic bacteria in the human gut: A simulated gastrointestinal study. NPJ Sci. Food.

[248] Ye A., Wang X., Lin Q., Han J., Singh H. (2020). Dynamic gastric stability and in vitro lipid digestion of whey-protein-stabilised emulsions: Effect of heat treatment. Food Chem..

[249] Mat D.J., Souchon I., Michon C., Le Feunteun S. (2020). Gastro-intestinal in vitro digestions of protein emulsions monitored by pH-stat: Influence of structural properties and interplay between proteolysis and lipolysis. Food Chem..

[250] Ye A. (2021). Gastric colloidal behaviour of milk protein as a tool for manipulating nutrient digestion in dairy products and protein emulsions. Food Hydrocoll..

[251] Reker D., Shi Y., Kirtane A.R., Hess K., Zhong G.J., Crane E., Lin C.-H., Langer R., Traverso G. (2020). Machine learning uncovers food-and excipient-drug interactions. Cell Rep..

[252] Mackie A., Gourcy S., Rigby N., Moffat J., Capron I., Bajka B. (2019). The fate of cellulose nanocrystal stabilised emulsions after simulated gastrointestinal digestion and exposure to intestinal mucosa. Nanoscale.

[253] El Kadri H., Devanthi P.V.P., Overton T.W., Gkatzionis K. (2017). Do oil-in-water (O/W) nano-emulsions have an effect on survival and growth of bacteria?. Food Res. Int..

[254] Zhang N., Zhou Q., Fan D., Xiao J., Zhao Y., Cheng K.-W., Wang M. (2021). Novel roles of hydrocolloids in foods: Inhibition of toxic Maillard reaction products formation and attenuation of their harmful effects. Trends Food Sci. Technol..

[255] Zhang R., Wu W., Zhang Z., Lv S., Xing B., Mcclements D.J. (2019). Impact of Food Emulsions on the Bioaccessibility of Hydrophobic Pesticide Residues in Co-Ingested Natural Products: Influence of Emulsifier and Dietary Fiber Type. J. Agric. Food Chem..

[256] Ke L., Zhang P., Xiang L., Wang H., Rao P., Wang S. (2020). Interaction of acrylamide with micelles in French fry aqueous extracts. Food Control.

[257] Li S., Zhou C., He Y., Liu H., Zhou L., Yu C., Wei C., Wang C. (2020). Novel Nanocellulose/Polymer Composite Aerogel as Solid-State Fluorescence Probe by Pickering Emulsion Route. Macromol. Mater. Eng..

[258] Gómez-Mascaraque L.G., Perez-Masiá R., González-Barrio R., Periago M.J., López-Rubio A. (2017). Potential of microencapsulation through emulsion-electrospraying to improve the bioaccesibility of β-carotene. Food Hydrocoll..

[259] Zhang Z., Zhang Z., Chang T., Wang J., Wang X., Zhou G. (2022). Phase change material microcapsules with melamine resin shell via cellulose nanocrystal stabilized Pickering emulsion in-situ polymerization. Chem. Eng. J..

[260] Zhang B., Zhang Z., Kapar S., Ataeian P., da Silva Bernardes J., Berry R., Zhao W., Zhou G., Tam K.C. (2019). Microencapsulation of phase change materials with polystyrene/cellulose nanocrystal hybrid shell via Pickering emulsion polymerization. ACS Sustain. Chem. Eng..

[261] Xia Q., Akanbi T.O., Wang B., Li R., Liu S., Barrow C.J. (2020). Investigation of enhanced oxidation stability of microencapsulated enzymatically produced tuna oil concentrates using complex coacervation. Food Funct..

[262] Rehman A., Tong Q., Jafari S.M., Korma S.A., Khan I.M., Mohsin A., Manzoor M.F., Ashraf W., Mushtaq B.S., Zainab S. (2021). Spray dried nanoemulsions loaded with curcumin, resveratrol, and borage seed oil: The role of two different modified starches as encapsulating materials. Int. J. Biol. Macromol..

[263] Lai H., Liu Y., Huang G., Chen Y., Song Y., Ma Y., Yue P. (2021). Fabrication and antibacterial evaluation of peppermint oil-loaded composite microcapsules by chitosan-decorated silica nanoparticles stabilized Pickering emulsion templating. Int. J. Biol. Macromol..

[264] Dupont H., Héroguez V., Schmitt V. (2022). Elaboration of capsules from Pickering double emulsion polymerization stabilized solely by cellulose nanocrystals. Carbohydr. Polym..

[265] Qian B., Zheng Z., Liu C., Li M., D’Sa R.A., Li H., Graham M., Michailidis M., Kantserev P., Vinokurov V. (2020). Microcapsules prepared via pickering emulsion polymerization for multifunctional coatings. Prog. Org. Coat..

[266] Yan B., Wang X., Zhang X., Liu S., Lu H., Ran R. (2022). One-step preparation of hydroxyapatite-loaded magnetic Polycaprolactone hollow microspheres for malachite green adsorption by Pickering emulsion template method. Colloids Surf. A Physicochem. Eng. Asp..

[267] Dupont H., Laurichesse E., Héroguez V., Schmitt V. (2021). Green Hydrophilic Capsules from Cellulose Nanocrystal-Stabilized Pickering Emulsion Polymerization: Morphology Control and Spongelike Behavior. Biomacromolecules.

[268] Loyeau P.A., Spotti M.J., Vinderola G., Carrara C.R. (2021). Encapsulation of potential probiotic and canola oil through emulsification and ionotropic gelation, using protein/polysaccharides Maillard conjugates as emulsifiers. LWT.

[269] Yao X., Yao X., Xu K., Wu K., Jiang F., Nishinari K., Phillips G.O. (2020). Iron encapsulated microstructured gel beads using an emulsification–gelation technique for an alginate-caseinate matrix. Food Funct..

[270] Zhang K., Xu Y., Lu L., Shi C., Huang Y., Mao Z., Duan C., Guo Y., Huang C. (2021). Hydrodynamic cavitation: A feasible approach to intensify the emulsion cross-linking process for chitosan nanoparticle synthesis. Ultrason. Sonochem..

[271] Zhang Z., Liu F., Lin Y. (2020). ZnO@ PNIPAM nanospheres synthesis from inverse Pickering miniemulsion polymerization. Colloids Surf. A Physicochem. Eng. Asp..

[272] Zhang J., Zhang W., Hao J., Li X., Xu D., Cao Y. (2022). In vitro digestion of solid-in-oil-in-water emulsions for delivery of CaCO_3_. Food Hydrocoll..

[273] Zhang Y., Jiang Y., Han L., Wang B., Xu H., Zhong Y., Zhang L., Mao Z., Sui X. (2018). Biodegradable regenerated cellulose-dispersed composites with improved properties via a pickering emulsion process. Carbohydr. Polym..

[274] Dehghani P., Hosseini S.M.H., Golmakani M.-T., Majdinasab M., Esteghlal S. (2018). Shelf-life extension of refrigerated rainbow trout fillets using total Farsi gum-based coatings containing clove and thyme essential oils emulsions. Food Hydrocoll..

[275] Xu J., Li X., Xu Y., Wang A., Xu Z., Wu X., Li D., Mu C., Ge L. (2021). Dihydromyricetin-Loaded Pickering Emulsions Stabilized by Dialdehyde Cellulose Nanocrystals for Preparation of Antioxidant Gelatin–Based Edible Films. Food Bioprocess Technol..

[276] Liu J., Song F., Chen R., Deng G., Chao Y., Yang Z., Wu H., Bai M., Zhang P., Hu Y. (2022). Effect of cellulose nanocrystal-stabilized cinnamon essential oil Pickering emulsions on structure and properties of chitosan composite films. Carbohydr. Polym..

[277] Xie B., Zhang X., Luo X., Wang Y., Li Y., Li B., Liu S. (2020). Edible coating based on beeswax-in-water Pickering emulsion stabilized by cellulose nanofibrils and carboxymethyl chitosan. Food Chem..

[278] Wardana A.A., Koga A., Tanaka F., Tanaka F. (2021). Antifungal features and properties of chitosan/sandalwood oil Pickering emulsion coating stabilized by appropriate cellulose nanofiber dosage for fresh fruit application. Sci. Rep..

[279] Trinh B.M., Smith M., Mekonnen T.H. (2022). A nanomaterial-stabilized starch-beeswax Pickering emulsion coating to extend produce shelf-life. Chem. Eng. J..

[280] Córdoba L.J.P., Sobral P.J. (2017). Physical and antioxidant properties of films based on gelatin, gelatin-chitosan or gelatin-sodium caseinate blends loaded with nanoemulsified active compounds. J. Food Eng..

[281] Chu Y., Gao C., Liu X., Zhang N., Xu T., Feng X., Yang Y., Shen X., Tang X. (2020). Improvement of storage quality of strawberries by pullulan coatings incorporated with cinnamon essential oil nanoemulsion. LWT.

[282] Zhu J., Tang C., Yin S., Yang X. (2018). Development and characterization of novel antimicrobial bilayer films based on Polylactic acid (PLA)/Pickering emulsions. Carbohydr. Polym..

[283] Ji N., Qin Y., Li M., Xiong L., Qiu L., Bian X., Sun Q. (2018). Fabrication and Characterization of Starch Nanohydrogels via Reverse Emulsification and Internal Gelation. J. Agric. Food Chem..

[284] Zhang L., Zaky A.A., Zhou C., Chen Y., Su W., Wang H., Abd El-Aty A., Tan M. (2022). High internal phase Pickering emulsion stabilized by sea bass protein microgel particles: Food 3D printing application. Food Hydrocoll..

[285] Zhu Y., Huan S., Bai L., Ketola A., Shi X., Zhang X., Ketoja J.A., Rojas O.J. (2020). High internal phase oil-in-water pickering emulsions stabilized by chitin nanofibrils: 3D structuring and solid foam. ACS Appl. Mater. Interfaces.

[286] El-Hammadi M.M., Small-Howard A.L., Fernández-Arévalo M., Martín-Banderas L. (2021). Development of enhanced drug delivery vehicles for three cannabis-based terpenes using poly (lactic-co-glycolic acid) based nanoparticles. Ind. Crops Prod..

[287] Wei Y., Sun C., Dai L., Zhan X., Gao Y. (2018). Structure, physicochemical stability and in vitro simulated gastrointestinal digestion properties of β-carotene loaded zein-propylene glycol alginate composite nanoparticles fabricated by emulsification-evaporation method. Food Hydrocoll..

[288] Takezaki H., Otsubo T., Echigo Y., Kamiya H., Okada Y. (2022). Porous and spherical ethyl cellulose fine particles produced by ternary system-based emulsion castings. Powder Technol..

[289] Nabata R., Tsudome M., Deguchi S. (2021). Preparation of cellulose microparticles having hierarchical internal structures from multiple emulsion templates. Colloids Surf. A Physicochem. Eng. Asp..

[290] Sun G., Yang L., Liu R. (2021). Thermal insulation coatings based on microporous particles from Pickering emulsion polymerization. Prog. Org. Coat..

[291] Pascaud K., Mercé M., Roucher A., Destribats M., Backov R.n., Schmitt V., Sescousse R., Brouillet F., Sarda S., Ré M.-I. (2022). Pickering emulsion as template for porous bioceramics in the perspective of bone regeneration. Colloids Surf. A Physicochem. Eng. Asp..

[292] Li Q., Hatakeyama M., Kitaoka T. (2022). Bioadaptive Porous 3D Scaffolds Comprising Cellulose and Chitosan Nanofibers Constructed by Pickering Emulsion Templating. Adv. Funct. Mater..

[293] Gong X., Yang P., Rohm K., Zhong Y., Zhao B., Manas-Zloczower I., Baskaran H., Feke D.L. (2021). Porous hollow fibers with controllable structures templated from high internal phase emulsions. J. Appl. Polym. Sci..

[294] Gong X., Rohm K., Su Z., Zhao B., Renner J., Manas-Zloczower I., Feke D.L. (2020). Porous hydrogels templated from soy-protein-stabilized high internal phase emulsions. J. Mater. Sci..

[295] Wang J., Qin J., Zhu H., Li B.G., Zhu S. (2021). Hierarchically Porous Monolith with High MOF Accessibility and Strengthened Mechanical Properties using Water-in-Oil High Internal Phase Emulsion Template. Adv. Mater. Interfaces.

[296] Zhou F.-Z., Yu X.-H., Zeng T., Yin S.-W., Tang C.-H., Yang X.-Q. (2019). Fabrication and characterization of novel water-insoluble protein porous materials derived from Pickering high internal-phase emulsions stabilized by gliadin–chitosan-complex particles. J. Agric. Food Chem..

[297] Zhao J., Zhang W., Gao C., Yin D. (2021). Shape stabilization of phase change material by polymerized high internal phase emulsion for thermal energy storage. Int. J. Energy Res..

[298] Zhou X., Zong X., Wang S., Yin C., Gao X., Xiong G., Xu X., Qi J., Mei L. (2021). Emulsified blend film based on konjac glucomannan/carrageenan/camellia oil: Physical, structural, and water barrier properties. Carbohydr. Polym..

[299] Oun A.A., Shin G.H., Kim J.T. (2022). Multifunctional poly (vinyl alcohol) films using cellulose nanocrystals/oregano and cellulose nanocrystals/cinnamon Pickering emulsions: Effect of oil type and concentration. Int. J. Biol. Macromol..

[300] Roy S., Rhim J.-W. (2021). Carrageenan/agar-based functional film integrated with zinc sulfide nanoparticles and Pickering emulsion of tea tree essential oil for active packaging applications. Int. J. Biol. Macromol..

[301] Wu M., Zhou Z., Yang J., Zhang M., Cai F., Lu P. (2021). ZnO nanoparticles stabilized oregano essential oil Pickering emulsion for functional cellulose nanofibrils packaging films with antimicrobial and antioxidant activity. Int. J. Biol. Macromol..

[302] Wang D., Ge X., Nie H., Yao Z., Zhang J. (2019). Demulsification-induced fast solidification: A novel strategy for the preparation of polymer films. Chem. Commun..

[303] Jahromi M., Niakousari M., Golmakani M.T. (2022). Fabrication and characterization of pectin films incorporated with clove essential oil emulsions stabilized by modified sodium caseinate. Food Packag. Shelf Life.

[304] Huang M., Wang H., Xu X., Lu X., Song X., Zhou G. (2020). Effects of nanoemulsion-based edible coatings with composite mixture of rosemary extract and ε-poly-l-lysine on the shelf life of ready-to-eat carbonado chicken. Food Hydrocoll..

[305] Liu Q., Zhang M., Bhandari B., Xu J., Yang C. (2020). Effects of nanoemulsion-based active coatings with composite mixture of star anise essential oil, polylysine, and nisin on the quality and shelf life of ready-to-eat Yao meat products. Food Control.

[306] Gull A., Bhat N., Wani S.M., Masoodi F.A., Amin T., Ganai S.A. (2021). Shelf life extension of apricot fruit by application of nanochitosan emulsion coatings containing pomegranate peel extract. Food Chem..

[307] Yuan D., Hao X., Liu G., Yue Y., Duan J. (2022). A novel composite edible film fabricated by incorporating W/O/W emulsion into a chitosan film to improve the protection of fresh fish meat. Food Chem..

[308] Liu D., Dang S., Zhang L., Munsop K., Li X. (2021). Corn starch/polyvinyl alcohol based films incorporated with curcumin-loaded Pickering emulsion for application in intelligent packaging. Int. J. Biol. Macromol..

[309] Mohammadian E., Alizadeh-Sani M., Jafari S.M. (2020). Smart monitoring of gas/temperature changes within food packaging based on natural colorants. Compr. Rev. Food Sci. Food Saf..

[310] Zhou X., Yu X., Xie F., Fan Y., Xu X., Qi J., Xiong G., Gao X., Zhang F. (2021). pH-responsive double-layer indicator films based on konjac glucomannan/camellia oil and carrageenan/anthocyanin/curcumin for monitoring meat freshness. Food Hydrocoll..

[311] Shin D.-M., Kim Y.-J., Yune J.-H., Kim D.-H., Kwon H.-C., Sohn H., Han S.-G., Han J.-H., Lim S.-J., Han S.-G. (2022). Effects of Chitosan and Duck Fat-Based Emulsion Coatings on the Quality Characteristics of Chicken Meat during Storage. Foods.

[312] Zhao R., Zhang Y., Chen H., Song R., Li Y. (2022). Performance of eugenol emulsion/chitosan edible coating and application in fresh meat preservation. J. Food Process. Preserv..

[313] Mauricio R.A., Campos J.A.D.B., Nassu R.T. (2022). Meat with edible coating: Acceptance, purchase intention and neophobia. Food Res. Int..

[314] Lin D., Kelly A.L., Miao S. (2021). Alginate-based emulsion micro-gel particles produced by an external/internal O/W/O emulsion-gelation method: Formation, suspension rheology, digestion, and application to gel-in-gel beads. Food Hydrocoll..

[315] Hu B., Han L., Ma R., Phillips G.O., Nishinari K., Fang Y. (2019). All-natural food-grade hydrophilic–hydrophobic core–shell microparticles: Facile fabrication based on gel-network-restricted antisolvent method. ACS Appl. Mater. Interfaces.

[316] Guan T., Liu B., Wang R., Huang Y., Luo J., Li Y. (2021). The enhanced fatty acids flavor release for low-fat cheeses by carrier immobilized lipases on O/W Pickering emulsions. Food Hydrocoll..

[317] Zhuang F., Li X., Hu J., Liu X., Zhang S., Tang C., Zhou P. (2018). Effects of casein micellar structure on the stability of milk protein-based conjugated linoleic acid microcapsules. Food Chem..

[318] Chen Y., Ge H., Zheng Y., Zhang H., Li Y., Su X., Panpipat W., Lai O.-M., Tan C.-P., Cheong L.-Z. (2020). Phospholipid–protein structured membrane for microencapsulation of DHA oil and evaluation of its in vitro digestibility: Inspired by milk fat globule membrane. J. Agric. Food Chem..

[319] Pereira A.R.L., Cattelan M.G., Nicoletti V.R. (2019). Microencapsulation of pink pepper essential oil: Properties of spray-dried pectin/SPI double-layer versus SPI single-layer stabilized emulsions. Colloids Surf. A Physicochem. Eng. Asp..

[320] Chen J.-F., Chen X.-W., Guo J., Yang X.-Q. (2019). Zein-based core–shell microcapsules for the potential delivery of algae oil and lipophilic compounds. Food Funct..

[321] Leng X., Cheng S., Wu H., Nian Y., Zeng X., Hu B. (2022). High Internal Phase Emulsions Stabilized with Polyphenol-Amyloid Fibril Supramolecules for Encapsulation and Protection of Lutein. J. Agric. Food Chem..

[322] Gao Z., Wu B., Hu B., Cui S., Xu L., Zhang K., Nishinari K., Phillips G.O., Fang Y. (2020). Novel strategy for enhancing the color intensity of β-Carotene: Enriching onto the oil-water interface. J. Colloid Interface Sci..

[323] Dou H., Li M., Qiao Y., Harniman R., Li X., Boott C.E., Mann S., Manners I. (2017). Higher-order assembly of crystalline cylindrical micelles into membrane-extendable colloidosomes. Nat. Commun..

[324] Chakrabarty A., Miyagi K., Maiti M., Teramoto Y. (2018). Topological transition in spontaneously formed cellulosic liquid-crystalline microspheres in aw/o emulsion. Biomacromolecules.

[325] Bai Y., Zhang F., Xu K., Wang X., Wang C., Zhang H., Tan Y., Wang P. (2021). Pickering emulsion strategy to control surface wettability of polymer microspheres for oil–water separation. Appl. Surf. Sci..

[326] Kramer S., Cameron N.R., Krajnc P. (2021). Porous polymers from high internal phase emulsions as scaffolds for biological applications. Polymers.

[327] Mudassir M.A., Aslam H.Z., Ansari T.M., Zhang H., Hussain I. (2021). Fundamentals and Design-Led Synthesis of Emulsion-Templated Porous Materials for Environmental Applications. Adv. Sci..

[328] Zhou F.-Z., Yu X.-H., Zhu J.-J., Yin S.-W., Yu Y.-G., Tang C.-H., Yang X.-Q. (2020). Hofmeister effect-assistant fabrication of all-natural protein-based porous materials templated from Pickering emulsions. J. Agric. Food Chem..

[329] Du J., Dai H., Wang H., Yu Y., Zhu H., Fu Y., Ma L., Peng L., Li L., Wang Q. (2021). Preparation of high thermal stability gelatin emulsion and its application in 3D printing. Food Hydrocoll..

[330] Wang H., Hu L., Du J., Peng L., Ma L., Zhang Y. (2021). Development of rheologically stable high internal phase emulsions by gelatin/chitooligosaccharide mixtures and food application. Food Hydrocoll..

[331] Wan Y., Wang R., Feng W., Chen Z., Wang T. (2021). High internal phase Pickering emulsions stabilized by co-assembled rice proteins and carboxymethyl cellulose for food-grade 3D printing. Carbohydr. Polym..

[332] Wu C., Na X., Ma W., Ren C., Zhong Q., Wang T., Du M. (2021). Strong, elastic, and tough high internal phase emulsions stabilized solely by cod myofibers for multidisciplinary applications. Chem. Eng. J..

[333] Wen Y., Che Q.T., Kim H.W., Park H.J. (2021). Potato starch altered the rheological, printing, and melting properties of 3D-printable fat analogs based on inulin emulsion-filled gels. Carbohydr. Polym..

[334] Shahbazi M., Jäger H., Chen J., Ettelaie R. (2021). Construction of 3D printed reduced-fat meat analogue by emulsion gels. Part II: Printing performance, thermal, tribological, and dynamic sensory characterization of printed objects. Food Hydrocoll..

[335] Zong Y., Kuang Q., Liu G., Wang R., Feng W., Zhang H., Chen Z., Wang T. (2022). All-natural protein-polysaccharide conjugates with bead-on-a-string nanostructures as stabilizers of high internal phase emulsions for 3D printing. Food Chem..

[336] Feng T., Fan C., Wang X., Wang X., Xia S., Huang Q. (2022). Food-grade Pickering emulsions and high internal phase Pickering emulsions encapsulating cinnamaldehyde based on pea protein-pectin-EGCG complexes for extrusion 3D printing. Food Hydrocoll..

[337] Pant A., Lee A.Y., Karyappa R., Lee C.P., An J., Hashimoto M., Tan U.-X., Wong G., Chua C.K., Zhang Y. (2021). 3D food printing of fresh vegetables using food hydrocolloids for dysphagic patients. Food Hydrocoll..

[338] Xia Y., Wu J., Wei W., Du Y., Wan T., Ma X., An W., Guo A., Miao C., Yue H. (2018). Exploiting the pliability and lateral mobility of Pickering emulsion for enhanced vaccination. Nat. Mater..

[339] Zhang Y., Jiao L., Wu Z., Gu P., Feng Z., Xu S., Liu Z., Yang Y., Wang D. (2022). Fabrication and characterization of Chinese yam polysaccharides PLGA nanoparticles stabilized Pickering emulsion as an efficient adjuvant. Int. J. Biol. Macromol..

[340] Wusiman A., He J., Cai G., Zhu T., Bo R., Liu Z., Hu Y., Wang D. (2022). Alhagi honey polysaccharides encapsulated into PLGA nanoparticle-based pickering emulsion as a novel adjuvant to induce strong and long-lasting immune responses. Int. J. Biol. Macromol..

[341] Mosquera M.J., Kim S., Zhou H., Jing T.T., Luna M., Guss J.D., Reddy P., Lai K., Leifer C.A., Brito I.L. (2019). Immunomodulatory nanogels overcome restricted immunity in a murine model of gut microbiome–mediated metabolic syndrome. Sci. Adv..

[342] Cai L., Cao M., Regenstein J. (2020). Slow-release and nontoxic Pickering emulsion platform for antimicrobial peptide. J. Agric. Food Chem..

[343] Larsson J., Williams A.P., Wahlgren M., Porcar L., Ulvenlund S., Nylander T., Tabor R.F., Sanchez-Fernandez A. (2022). Shear-induced nanostructural changes in micelles formed by sugar-based surfactants with varied anomeric configuration. J. Colloid Interface Sci..

[344] Dos Santos E.C., Belluati A., Necula D., Scherrer D., Meyer C.E., Wehr R.P., Lörtscher E., Palivan C.G., Meier W. (2020). Combinatorial Strategy for Studying Biochemical Pathways in Double Emulsion Templated Cell-Sized Compartments. Adv. Mater..

[345] Ma Y., Gao Y., Zhao X., Zhu Y., Du F., Hu J. (2018). A Natural Triterpene Saponin-Based Pickering Emulsion. Chem.–A Eur. J..

[346] Shin J., Seo S.-M., Park I.-K., Hyun J. (2021). Larvicidal composite alginate hydrogel combined with a Pickering emulsion of essential oil. Carbohydr. Polym..

[347] He J., Ma Z., Yang Y., Hemar Y., Zhao T. (2020). Extraction of tetracycline in food samples using biochar microspheres prepared by a pickering emulsion method. Food Chem..

[348] Zaharia C., Diaconu M., Muresan E.I., Danila A., Popescu A., Rosu G. (2020). Bioactive emulsions with beneficial antimicrobial application in textile material production. Cellulose.

[349] Zheng Y., Oguzlu H., Baldelli A., Zhu Y., Song M., Pratap-Singh A., Jiang F. (2022). Sprayable cellulose nanofibrils stabilized phase change material Pickering emulsion for spray coating application. Carbohydr. Polym..

[350] He Y., Li S., Zhou L., Wei C., Yu C., Chen Y., Liu H. (2021). Cellulose nanofibrils-based hybrid foam generated from Pickering emulsion toward high-performance microwave absorption. Carbohydr. Polym..

[351] Li Y., Yu D., Wang X., Wang Q., Zhang Z., Liu W. (2022). Lauric arginate/cellulose nanocrystal nanorods-stabilized alkenyl succinic anhydride pickering emulsion: Enhancement of stabilization and paper sizing performance. Cellulose.

[352] Xu G., Nigmatullin R., Koev T.T., Khimyak Y.Z., Bond I.P., Eichhorn S.J. (2022). Octylamine-modified cellulose nanocrystal-enhanced stabilization of Pickering emulsions for self-healing composite coatings. ACS Appl. Mater. Interfaces.

[353] Seo H.M., Seo M., Shin K., Choi S., Kim J.W. (2021). Bacterial cellulose nanofibrils-armored Pickering emulsions with limited influx of metal ions. Carbohydr. Polym..

[354] Perrin L., Gillet G., Gressin L., Desobry S. (2020). Interest of pickering emulsions for sustainable micro/nanocellulose in food and cosmetic applications. Polymers.

[355] Bouhoute M., Nakajima M., Isoda H. (2021). Design of nanoemulgel using Argania spinosa microfibrillated cellulose and natural emulsifiers foreseeing melanogenesis enhancement. Carbohydr. Polym..

[356] Musazzi U.M., Franzè S., Minghetti P., Casiraghi A. (2018). Emulsion versus nanoemulsion: How much is the formulative shift critical for a cosmetic product?. Drug Deliv. Transl. Res..

